# A fragment-based approach leading to the discovery of a novel binding site and the selective CK2 inhibitor CAM4066

**DOI:** 10.1016/j.bmc.2017.04.037

**Published:** 2017-07-01

**Authors:** Claudia De Fusco, Paul Brear, Jessica Iegre, Kathy Hadje Georgiou, Hannah F. Sore, Marko Hyvönen, David R. Spring

**Affiliations:** a Department of Chemistry, University of Cambridge, Lensfield Road, Cambridge CB2 1EW, UK; b Department of Biochemistry, University of Cambridge, 80 Tennis Court Road, Cambridge CB2 1GA, UK

**Keywords:** CK2, Fragment-based drug discovery, Kinase inhibition, Fragment linking, Molecular modelling

## Abstract

Recently we reported the discovery of a potent and selective CK2α inhibitor **CAM4066**. This compound inhibits CK2 activity by exploiting a pocket located outside the ATP binding site (αD pocket). Here we describe in detail the journey that led to the discovery of **CAM4066** using the challenging fragment linking strategy. Specifically, we aimed to develop inhibitors by linking a high-affinity fragment anchored in the αD site to a weakly binding warhead fragment occupying the ATP site. Moreover, we describe the remarkable impact that molecular modelling had on the development of this novel chemical tool. The work described herein shows potential for the development of a novel class of CK2 inhibitors.

## Graphical abstract

**Figure:**
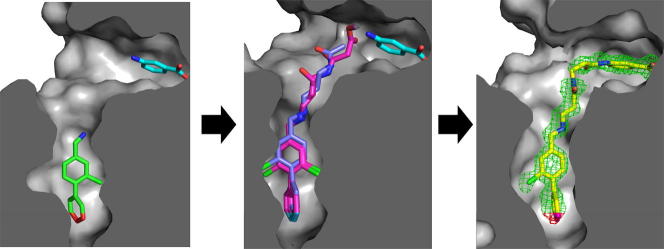

## 1 Introduction

### 1.1 Fragment-based drug discovery

Fragment-based drug discovery (FBDD) is a structure-based approach used to provide lead compounds to target biological systems. Although initial hits usually have lower potency than those derived from High-Throughput Screening campaigns (HTS), FBDD is considered to be more efficient in the optimization phases of drug discovery.^1^, ^2^ In a typical FBDD project, a fragment library is screened using sensitive biophysical techniques. Secondly, X-ray crystallography or NMR are used to investigate the binding modes of the fragment hits in the protein of interest and thereby characterise the fragments’ binding pose.^1^, ^2^, ^3^ The third step is fragment elaboration, typically occurring via growing, merging and/or linking strategies. In the growing strategy optimised fragment hit is grown in a step-wise fashion, engaging with the target through additional interactions.^3^ The merging strategy sees the best features of several overlapping fragments merged into single molecule with higher potency.^3^ In the linking strategy, fragments binding in different parts of the target site are linked together via rigid or flexible linkers.^4^ The linked compound should have a more favourable ΔG of binding than the sum of the ΔG values of the individual fragments according to the concept of ‘super-additivity’.^5^, ^6^, ^7^ However this last strategy has been considered to be the most difficult of the three methods applied in FBDD.^3^ Ideally, the linker should maintain the optimal binding configurations that have been adopted by the individual fragments and should establish additional interactions with the protein to prevent a loss in ligand efficacy (LE).^3^ In practise the linkers often constrain the molecule too much resulting in suboptimal interactions with the target.

### 1.2 CK2 as an anticancer target

CK2 is a hetero-tetrameric enzyme composed of two catalytic subunits (α and/or α′) and two regulatory subunits (β and/or β′) which confer stability, control selectivity and enhance enzyme activity.^6^, ^7^ It is an unusual kinase in that it is believed to be constitutively active, without needing external stimulus, such as phosphorylation of the activation loop.

CK2 is involved in multiple intracellular pathways including the regulation of cell proliferation and cell growth. It is also believed to be an apoptosis-suppressor in both healthy and cancer cells.^8^, ^9^

Among other features, cancer cells show dysregulated proliferation and apoptotic activity leading to uncontrolled cell growth.^10^ CK2 has been found to be overexpressed in a range of cancer cell lines including prostate, breast, colon cancer.^11^ As reported by Trembley and co-workers, the dysregulated expression of CK2 in cancer cells is an index of the pathological status of the tumour. Their studies have also shown that downregulation of CK2 decreases cell growth and cell proliferation and increases apoptotic activity.^12^ Therefore, CK2 is crucial for cell survival, and the absence of redundant pathways to compensate for its downregulation makes cancer cells more sensitive to CK2 inhibition.^11^, ^12^ Hence, CK2 inhibition represents an attractive anticancer target.

The most common strategy to inhibit CK2 is through small molecules that target the ATP-binding catalytic site.^6^, ^13^, ^14^ However, a drawback of ATP competitive inhibitors is that they suffer from selectivity issues, leading to off-target effects.^9^, ^15^

Therefore, as with other kinases, increased interest has arisen in the development of inhibitors that do not target the conserved ATP site and demonstrate better selectivity. CK2 inhibition exploiting allosteric binding sites has been achieved using a variety of approaches which target CK2 substrates,^16^ the regulatory β subunit^17^ or the α/β interface.^18^, ^19^, ^20^, ^21^

Recently, we reported the discovery of a new binding pocket, the αD site, within the catalytic CK2α subunit and described the development of a novel class of inhibitors of CK2α utilising this pocket (**CAM4066**). **CAM4066** has nanomolar affinity for CK2α and clearly increased selectivity relatively to other known CK2 inhibitors.^22^

Herein we present in detail the optimization of the initial fragment bound in the αD pocket into the selective inhibitor **CAM4066** by the successful combination of fragment growing and linking.

## 2 Material and methods

### 2.1 Chemistry

#### 2.1.1 Experimental procedures

**Scheme 1:**
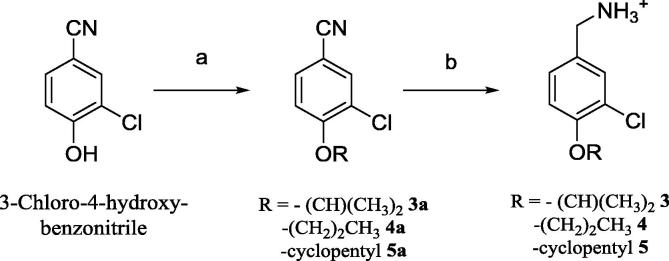
(a) DMF, RX, Na_2_CO_3_, (b) i) LiAlH_4_, Et_2_O, AlCl_3_, ii) Et_2_O, 2 M HCl in Et_2_O. General procedures are detailed in the Supporting information.

Compounds **3**–**5** were obtained from the commercially available 3-chloro-4-hydroxy-benzonitrile via the synthetic route reported in Scheme 1.

**Scheme 2:**
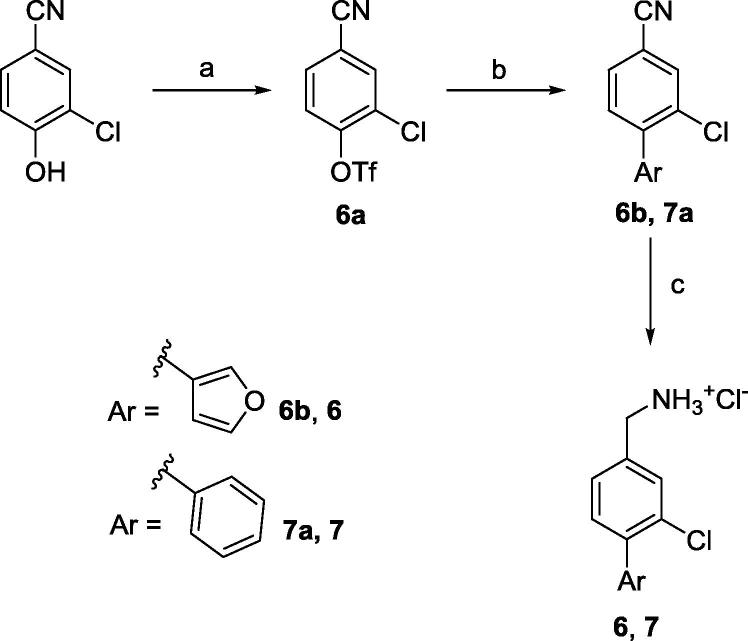
(a) CH_2_Cl_2_, Py, Tf_2_O, (b) ArB(OH)_2_, LiCl, DCE, Pd(PPh_3_)_4_, (c) i) LiAlH_4_, Et_2_O, AlCl_3_, ii) Et_2_O, 2 M HCl in Et_2_O.General procedures are detailed in the Supporting information.

Synthesis of **6** and **7** was obtained starting from aryl triflation of 3-chloro-4-hydroxy-benzonitrile to give compound **6a** which then underwent Suzuki-Miyaura cross-coupling to provide compounds **6b** and **7a** followed by nitrile reduction. Conversion of the amines into hydrochloride salts provided the biaryls **6** and **7** as shown in Scheme 2.

**Scheme 3:**
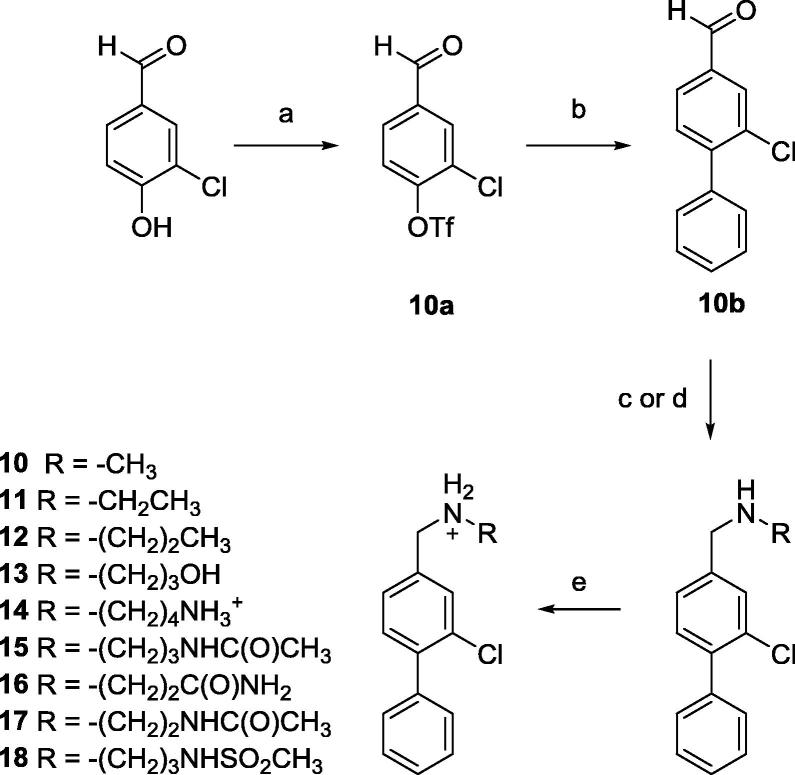
(a) CH_2_Cl_2_, Py, Tf_2_O, (b) ArB(OH)_2_, LiCl, DCE, Pd(PPh_3_)_4_, (c) compounds **10**, **12**–**15**: RNH_2_, DCE, NaBH(OAc)_3_, (d) compounds **11**, **16**–**18**: RNH_3_^+^, MeOH, Et_3_N, NaBH(OAc)_3_, (e) Et_2_O, 2 M HCl in Et_2_O. General procedures are detailed in the Supporting information.

Compounds **10**–**18** were prepared by the general method in Scheme 3. Compound **10b**, which was obtained via Suzuki coupling of **10a** and phenylboronic acid, underwent reductive amination with the appropriate amines and then treated with hydrochloric acid to provide the final compounds.

**Scheme 4:**
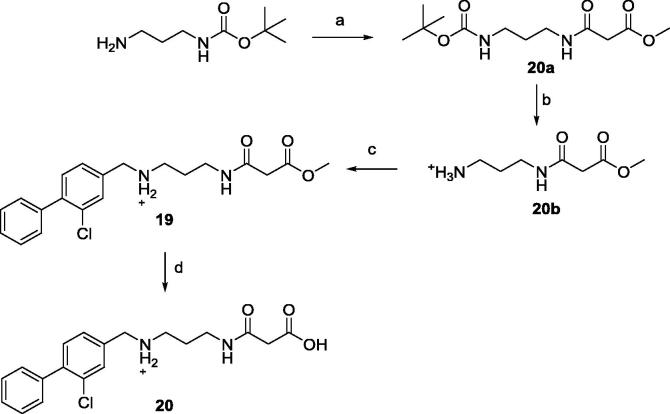
(a) CH_2_Cl_2_, 3-chloro-3-oxoproprionate, NaHCO_3_, (b) TFA/CH_2_Cl_2_, (c) MeOH, Et_3_N, NaBH(OAc)_3_, (d) LiOH,THF, 4 M HCl in dioxane, CH_2_Cl_2_. General procedures are detailed in the Supporting information.

Compound **20** was synthesized by methyl ester hydrolysis of its precursor **19**. The synthesis of **19** started from treatment of the commercially available *N-*Boc-1,3-propanediamine with methyl 3-chloro-3-oxopropionate to provide **20a** which was then converted to the TFA salt **20b**. Compound **20b** underwent reductive amination in the presence of **10b** to give **20c** (Scheme 4).

**Scheme 5:**
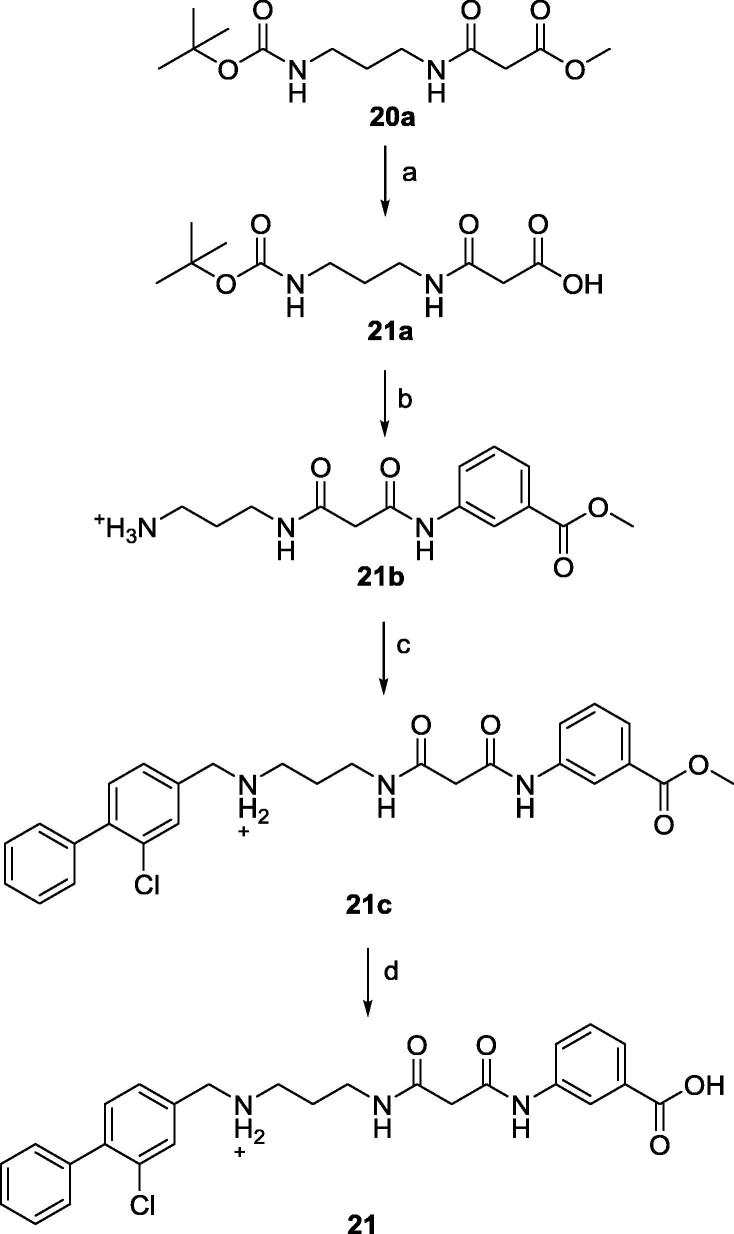
(a) LiOH,THF, 4 M HCl in dioxane, CH_2_Cl_2_, (b) EDC-HCl, NMM, **(**c) RNH_3_^+^, MeOH, Et_3_N, NaBH(OAc)_3_, (d) LiOH,THF, 4 M HCl in dioxane.

Synthesis of **21** commenced with ester hydrolysis of **20a** followed by coupling with commercially available *m*-amino benzoate to give **21c**. The latter intermediate underwent reductive amination to provide **21d** which was then hydrolysed to obtain **21** (Scheme 5).

#### 2.1.2 Detailed procedures and characterization

Detailed procedures and characterization of all the compounds reported in the paper and their precursors can be found in the Supporting information.

### 2.2 Biophysical assays

#### 2.2.1 Protein expression and purification, X-ray crystallography, phosphorylation assays, ITC

Protein expression and purification, X-ray crystallography, phosphorylation assays and ITC protocols can be found in the Supporting information of our recently published paper.^22^

### 2.3 Molecular modelling

Molecular modelling was performed using Glide from the Schroedinger package using default parameters.^23^, ^24^, ^25^ Details can be found in the Supporting information.

## 3 Results and discussion

**Fig. 1:**
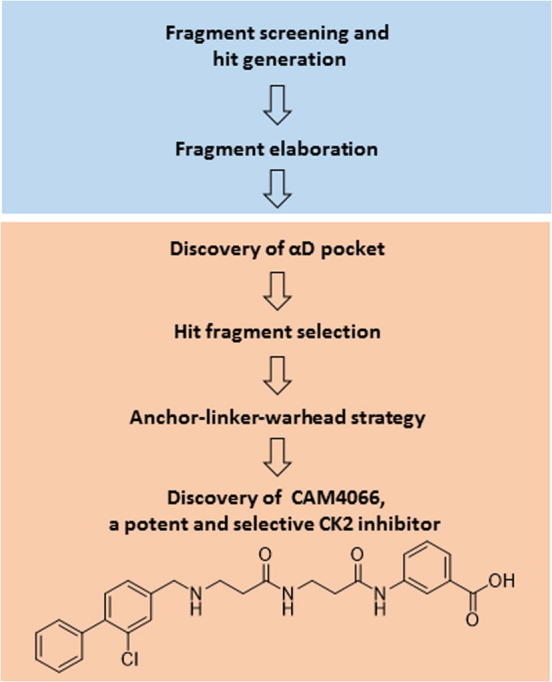
Overview of the workflow from fragment screening against the α-β interface of CK2 to the discovery of a novel binding site and development of **CAM4066**.

Since it had previously been reported that the α/β interface could be used for the allosteric inhibition of CK2α,^7^, ^20^, ^21^ we decided to target the α/β interface using FBDD. We ran a fragment screen targeting α/β interface on the catalytic CK2α subunit and identified a number of fragments binding to this site. While we were optimising the fragment hits on the α/β interface, one of the fragment analogues revealed a previously unseen pocket near the ATP-binding site, suggesting a novel and effective method of inhibiting CK2α (Fig. 1). The first inhibitor to utilise this newly identified pocket was **CAM4066**, a nanomolar inhibitor with good selectivity for CK2α.^22^

### 3.1 Fragment screening to find hits to target CK2

**Fig. 2:**
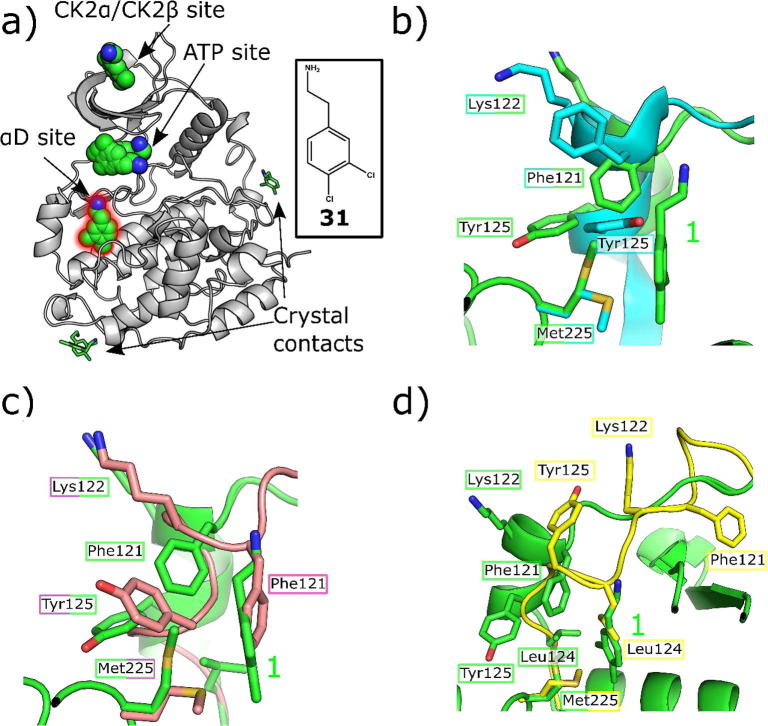
a) Crystallographic structure of CK2α (grey) and 6 molecules of **1** (green). The promiscuous fragment occupies various sites of the protein showing potential for allosteric inhibitors (PDB code 5CLP). The molecules occupying biologically relevant sites are highlighted by sphere representation. The molecules at crystal contacts and therefore not of interest are shown as sticks. b) The movement of the important residues in the αD site upon the binding of 1 (PDB: 5CLP) compared to the partly open apo structure (PDB: 5CVH). c) The movement of the important residues in the αD site upon the binding of 1 (PDB: 5CLP) compared to the closed apo structure (PDB: 3FWQ) d) The movement of the important residues in the αD site upon the binding of 1 (PDB: 5CLP) compared to the inactive structure (PDB: 5CVG).

As reported previously, **1** was observed in crystal structures bound to multiple sites on CK2α during a fragment-screening program against CK2 (Fig. 2).^22^ Three of these sites were at crystal contacts and therefore of little interest, but the other three sites were of biological significance. Electron density for the ligand was observed at the CK2α/β interface, in the ATP-binding active site and, most unexpectedly, at a previously unreported site located behind the αD helix, which we have named the αD site. This site is formed by the movement of the flexible αD helix which opens up a deep hydrophobic pocket adjacent to the ATP site. The αD helix is significantly more flexible in CK2α than in any other kinases and as such is seen to adopt multiple positions in different crystal structures. There are 3 reported apo structures; the closed conformation, where the αD pocket is filled by Phe121 (PDB: 3FWQ), the partially open conformation (5CVH) where Tyr125 partially fills the pocket and an inactive conformation where the αD pocket is filled by Leu124 (PDB: 5CVG) which distorts the hinge region and prevents ATP binding. To allow the binding of **1** the residue that fills the αD site in the different apo forms (Leu124, Tyr125 or Phe121) is displaced and Met225 rotates opening the bottom of the αD site. The amine of the fragment forms hydrogen bonds with the backbone carbonyl of Pro159, two waters at the top of the pocket and forms possible π-cation interactions with Phe121. The dichlorophenyl part of the fragment sits deep in the pocket interacting with hydrophobic core of the pocket, exposed when Met225 moves out of the pocket.

### 3.2 Lead generation

Prior to conducting any studies to assess the potential of the αD site, a chemical tool to probe the αD site had to be developed from **1**, given that it bound weakly to multiple sites and therefore was of no use to probe the function of one site. The initial plan was to pursue a fragment linking strategy in order to quickly develop inhibitors that would bind at the αD site but inhibit by linking to fragments in the ATP site. This strategy would have 4 phases;

1. *Fragment optimisation*: The aim was to develop fragments with a higher affinity and selectivity for the αD site than **1**, as **1** was found binding to a number of different sites and hence thought to be less than ideal as a starting point for the development of specific inhibitor.
2. *ATP site fragment identification*: Warhead fragments bound in the ATP site would be identified using X-ray fragment screening. Ideally, such fragment would interact with the ATP site only weakly so that the affinity for CK2α would be dominated by the affinity for the non-conserved αD site and thus drive selectivity and ATP site warhead would provide only steric hindrance to displace the nucleotide from the kinase. The chosen fragment would also contain a suitable chemical functionality in a correct position for linker attachment.
3. *Linker optimisation*: Fragment linking is seen as the more challenging route of fragment optimisation as introducing a linker that does not interfere with the binding mode of the original fragments is difficult. Therefore, the optimised αD site fragment would be iteratively grown towards the ATP site in an attempt to generate an efficient linker.
4. *Fragment linking*: Finally, the optimised αD site fragment would be linked to the weakly binding ATP site fragment using the knowledge gained from the linker optimisation to give the final chemical tool which will be used to probe the potential of the αD site to develop selective CK2α inhibitors.

#### 3.2.1 Fragment optimisation

Promiscuity was a particularly acute problem with the original fragment **1** as it was observed to bind to the ATP site, the interface and the αD site. Therefore, the initial aim was to identify more selective αD binding fragment, at the expense of the ATP and α-β interface sites.

**Fig. 3:**
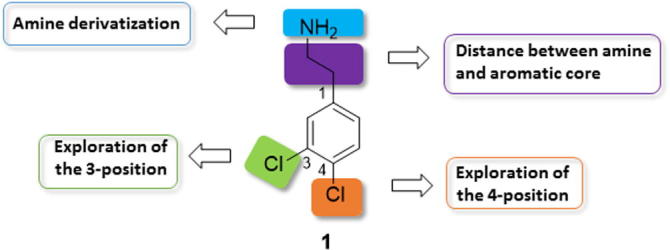
Schematic representation of the fragment elaboration carried out around **1** to develop a lead fragment to inhibit CK2.

Firstly, commercially available analogues of **1**, identified from the Zinc database,^26^ were screened *in silico* against the αD site. From this screen, a number of commercially available compounds were purchased and co-crystal structures were obtained to determine the binding mode. These compounds explored a range of structures around the initial fragment and included variations in the distance from the hydrophobic core to the amine group as well as changes in the substitution pattern at the 3 and 4 positions (Fig. 3).

**Fig. 4:**
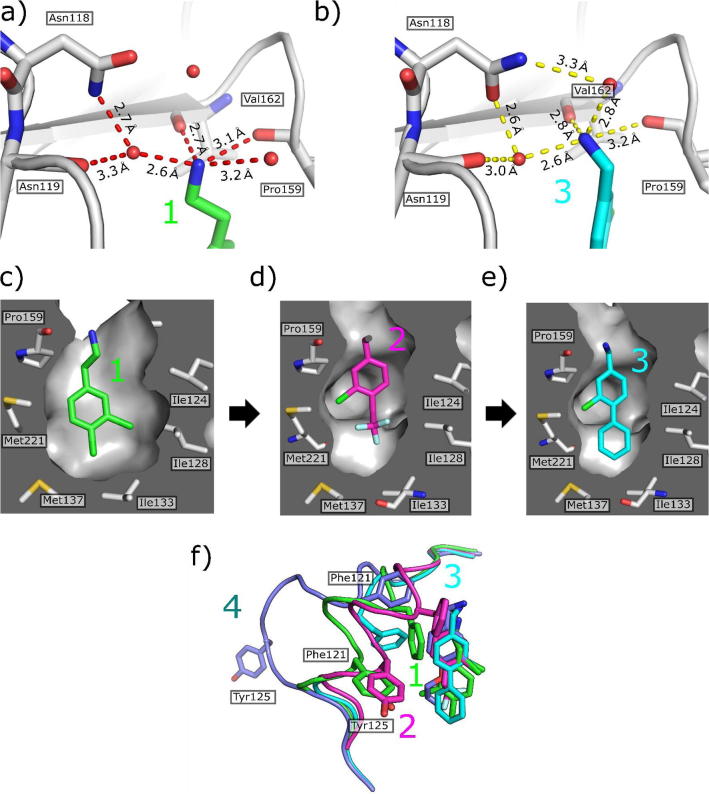
The optimisation of the αD site fragment. a) The interactions of the amine of **1** with the backbone carbonyls of Val162 and Pro159 along with the interaction with Asn118 and Asn119 via a water bridge (PDB: 5CLP). b) The interactions of the amine of **7** with the backbone carbonyls of Val162 and Pro159 along with the interaction with Asn118 and Asn119 via a water bridge (PDB: 5CHS). Since the amine of **7** sits higher up in the pocket, it pulls down the top water into hydrogen bonding distance, thereby forming another water bridge to Asn118. c) The hydrophobic core of **1** sits in the hydrophobic pocket of the αD site (PDB: 5CLP), however there is still potential to optimise the interactions with this pocket. d) From the crystal structure it appears that **2** is more selective for the αD site over the ATP site, however, the OCF_3_ group does not fill the hydrophobic pocket of the αD site (PDB: 5CVF). e) The crystal structure of **7** bound in the αD site shows that the molecule fills the hydrophobic core of the αD pocket more efficiently (PDB: 5CHS). f) Movement of the αD loop upon binding of compounds **1** (green), **2** (magenta), **3** (cyan) and **4** (light blue).

Analysis of the structure of **1** bound to CK2α indicated that the amine provided vital hydrogen bonds with the backbone carbonyls of Val162 and Pro159 at the mouth of the pocket (Fig.4a and c). The crystal structure of **2** (Fig.4d), bearing a trifluoromethoxy group in the 4-position and a shorter linker to the amine, showed that the compound bound selectively in the αD site and appears to bind neither to the ATP nor the interface sites. As predicted, the amine of **2** retained the interactions with the backbone carbonyls of Pro159 and Val162. The crystal structures indicated that there was space for optimization around the OCF_3_ group of **2** (Fig.4d). Therefore, the subsequent optimization of **2** focused upon the modification of the 4-position of the benzyl ring in order to increase affinity for the bottom of the αD site.

Based on the crystal structure of **2**, a series of fragments with modifications in the 4 position were designed *in silico* and synthesized (**3**–**7**, Table 1)). All 5 of these fragments were soaked into CK2α crystals and their complex structures determined. These structures showed that all new fragments bound as predicted, in the αD site, with **6** and **7** showing some weak density at the α/β interface site. The R-groups in the 4 position all filled the pocket formed by the movement of Met225. However, the electron density for the groups in the 4 position was poorly defined for all groups apart from those in **6** and **7** in which the phenyl group or furan group stacks against Met225. The structures of all of these compounds showed that the binding of the fragments caused a significant movement of the αD loop but by different amounts in each structure (Fig.4f). In the co-crystal structure of **1** and CK2α_FP10 (Fig.4f, blue), a small movement of 3 Å brings Tyr125 out from being buried underneath the αD loop and allows the fragment to bind. However, when **4** bound a greater displacement of the loop by 24 Å occurred, which led to a subsequent increase in the size of the αD pocket (Fig.4f, dark blue). It was unclear as to why the loop moved significantly more in the structure of **4**, however, it is likely that in solution the αD loop is flexible and free to move upon the binding of the fragments but the crystal structures only capture one of a range a of possible conformations. The affinities of these fragments towards the αD pocket was then determined by ITC (Table 1) (Fragment_aD_site_optimisation.pse).

**Table 1:** Structures and K_d_ values of the fragments showing selective binding in the αD pocket over the ATP site and the interface.

**Figure:**
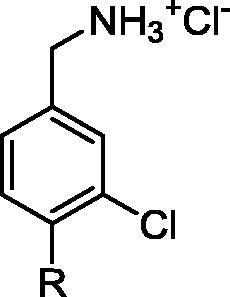

Compound	Structure R =	Affinity (ITC) K_d_ (μM)	PDB
**2**	–OCF_3_	NA	5CVF
**3**		300	5MOD
**4**		629	5CS6
**5**		500	
**6**		520	5MOE
**7**		270	5CSH

The crystal structures and binding affinities of the fragments showed that **7** was the most interesting of these fragments. It binds with the phenyl group deep in the hydrophobic αD site and had the highest affinity of 270 μM (co-crystal structure shown in Fig.4b and e). However, these compounds did not show any inhibition of the kinase activity of CK2α as they were not binding in the ATP site. Indeed, no electron density corresponding to any of the compounds from this stage was observed in the ATP site and good electron density for ADP or ATP was observed in the ATP sites with **6** or **7** bound. This data led to **7** being selected as the optimised αD site fragment for further elaboration.

#### 3.2.2 ATP site fragment identification

**Fig. 5:**
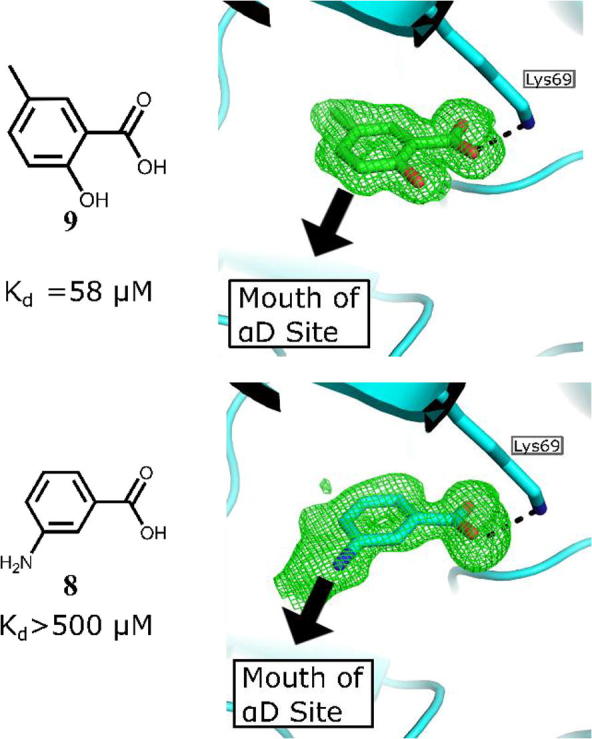
Electron density of fragments **8** (PDB code 5CSP) and **9** (PDB code 5CSV) in the ATP binding site. The protein is represented in cyan and the H-bond between the fragments and the protein is shown as a black dashed line.

In order to obtain a suitable fragment to use as the warhead in the ATP site, a 352 member fragment library, optimised for X-ray screening (purchased from Zenobia), was screened against CK2α. The screening was performed by soaking the fragments into crystals as cocktails of four fragments. Of these, 23 fragments showed weak electron density in the ATP site. These compounds could be divided into 3 groups, based on their binding mode: hinge binders (2 fragments, PDB: 5MOT and 5MOW), Lys69 binders (11 fragments, for example PDB: 5MOH and 5MOV) and fragments that interact with both the hinge region and Lys69 (10 fragments, example PDB: 5CSV and 5CSP). The hinge region is very conserved amongst kinases^27^ (Fig. SI_1, Supporting information) and as such fragments binding to the hinge would interact with many other kinases. We therefore focused our efforts on the Lys69 binding fragments. Fragments interacting with Lys69 were dominated by structures with a benzoic acid core with the carboxylic acid interacting with terminal amine of Lys69, similarly to what is seen with well characterised CK2α inhibitor CX-4945. These compounds were ranked using a thermal shift assay (results can be found in the Supporting information), as we had previously observed that the thermal shift assay was effective for detecting ATP site binding ligands (unpublished data). These compounds gave shifts of between 0 and +4.1 °C and the data confirmed that the benzoic acid core was a promising fragment to proceed with. One of the compounds (**9**) that gave the highest shift was then further investigated in a phosphorylation inhibition assay and its K_d_ determined via ITC. This showed Compound **9** has a K_d_ of 58 μM (Fig. 5) and that its inhibition in the phosphorylation assay was approximately 100 µM.

However, although **9** had for a fragment a very high affinity, it was not chosen as the ATP site fragment for a number of reasons. Firstly, the aim was to validate the concept of using the αD site to develop a selective CK2α inhibitor and therefore the affinity for CK2α must be dominated by binding in the αD site rather than the ATP site. Secondly, **9** did not contain a synthetically tractable handle in the position we envisioned linking to.

We therefore re-examined the remaining benzoic acid fragments that interacted only with Lys69. Inspection of the crystal structure and investigation of the affinity of Fragment **8** for CK2α showed it fulfilled all the criteria set out for a suitable fragment: it did not interact with the hinge region, it had very low affinity (>500 µM) and it contained a suitable synthetic handle for linking into αD binding compounds (Fig. 5). Therefore **8** was chosen as the fragment to link to in the ATP site, however, this choice would be evaluated again once the linker was developed further.

#### 3.2.3 Linker optimisation

**Fig. 6:**
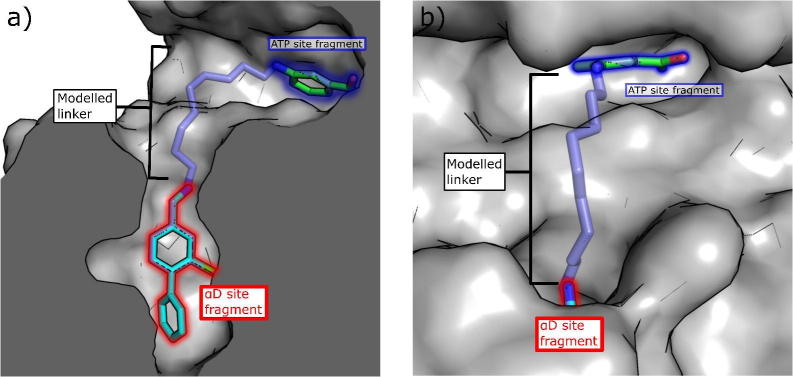
Modelling studies of potential linkers connecting fragments **7** and **8**: a) A 9-atom linker needed to link the ATP site and the αD site. b) The proposed linker seen from above. Original fragments, as observed in crystal structures are highlighted with red and blue and the modelled linker if shows as semitransparent sticks.

With the optimised anchor fragment **7** and the ATP site-binding warhead **8** in hand, the challenge was to develop a suitable linker to unite the two fragments. Fragment linking is seen as a promising technique for fragment optimisation due the possibility of super-additivity, but it is rarely fully successful due to the many challenges in the design of the linker. Whittaker and co-workers suggested that for the highest chance of success, the binding of one of the starting fragments should be dominated by polar interactions and the other by hydrophobic interactions as this allows greater flexibility in the binding mode of one of the partners.^28^ This was promising in our case since the binding of the benzoic acid **8** is dominated by polar interactions in the ATP site and **7** binds to the largely hydrophobic αD pocket. The amine of **7** was the best point to grow out of the αD pocket towards the ATP site with the aim of linking to the amine of **8**. The initial modelling studies indicated that the minimum length required to link **7** and **8** would be nine atoms (Fig. 6). However, the length of this linker was envisioned to be a problem as high flexibility is a well-established unwanted characteristic in inhibitor development.^29^ Therefore, rather than simply linking the two fragments it was decided to iteratively grow **7** out of the αD site in an attempt to generate a linker that interacted with the target while extending towards the ATP site. To this end, a series of analogues of **7** were synthesized with variations at the amine, thereby growing out of the αD pocket towards the ATP site (Table 2). This process was guided by continuous structural analysis of the binding mode followed by analysis of the compounds ability to inhibit in the phosphorylation assay and determination of its affinity by ITC (Linker_optimisation.pse).

**Table 2:** Structure and % inhibition of kinase activity at 500 μM of all the compounds with variable length linkers.

**Figure:**
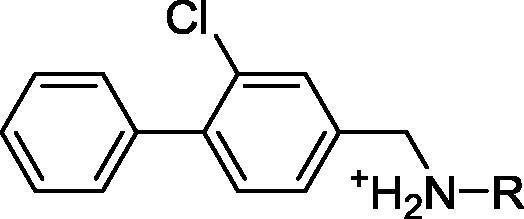

Compound	R	PDB	% Inhibition at 500 μM
**10**	–CH_3_	5MP8	44 ± 12
**11**	–CH_2_CH_3_	5MPJ	23 ± 6
**12**	–CH_2_CH_2_CH_3_	5MMF	33 ± 5
**13**		5CTO	47 ± 5
**14**		5MMR	18 ± 2
**15**		5CTP	39 ± 3
**16**		5MO7	16 ± 6
**17**		a	12 ± 4
**18**		5MO5	51 ± 10
**19**		5CU2	46 ± 2
**20**		5MO6	20 ± 4

a Poor or no electron density observed for the ligand.

Initially, aliphatic chains of different length were investigated to establish the tolerance of the linker channel to different substituents. The methyl and ethyl groups of compound **10** and **11** occupied a small lateral pocket, however, **11** exhibits two conformations one of which occupied the channel that links the αD site and ATP site. Therefore, the chain was extended further as in **12**. In this compound the three-carbon atom linker no longer occupied the lateral pocket but bound only in the channel formed by Met163. Interestingly, in the crystal structures with either **10** or **11** bound in the αD site, ADP was visible in the ATP site, whereas when **12** bound, the nucleotide was no longer visible. However, this was not reflected in the inhibition observed as all three compounds showed only weak inhibition of phosphorylation by CK2α.

**Fig. 7:**
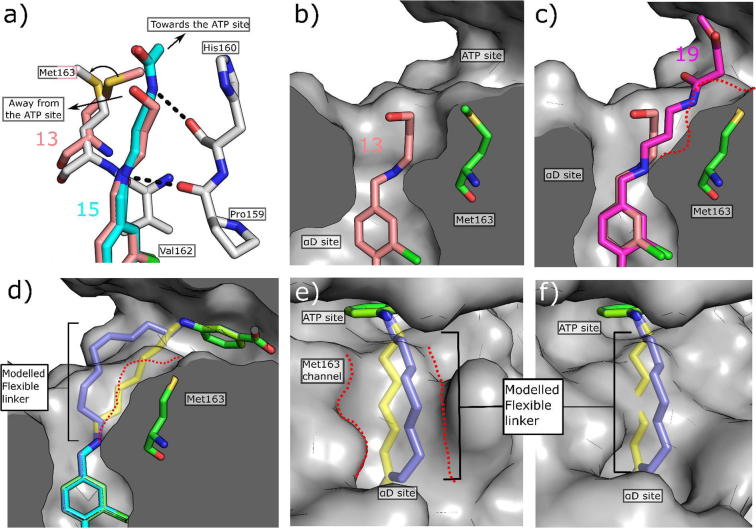
Conformational changes of linker channel. (a) The crystal structure of **13** (PDB: 5CT0, pink) with **15** (PDB: 5CTP, light blue) superimposed. When **13** binds, Met163 does not move therefore the channel to the ATP site does not open and the surface of CK2α is clearly seen to block the binding of **15**. The linker of **15** also forms a hydrogen bonding network with back bone carbonyls within the channel. b) The surface representation of the crystal structure of **13** (PDB: 5CT0) bound to CK2α. c) The surface representation of the crystal structure of **19** (PDB: CX9, purple) bound to CK2α with the structure of **13** (pink) superimposed. The outline of the surface when Met163 blocks the channel is represented by a red dotted line. d) Overlay of the modelled 7 atom (yellow) and 9 atom linkers on to the crystal structures of the fragments **7** (5CSH, light blue) and **8** (green) when Met163 has flipped to open the channel. The outline of the surface when Met163 blocks the channel is shown as a red dashed line; e) Overlay of the modelled 7 atom linker (yellow) and 9 atom linker (dark blue) when Met163 is flipped and forms the linker channel. The crystal structure of the ATP site binding fragment **8** is shown (green, 5CSP) The sides of the channel are highlighted by a red dashed line; f) Overlay of the modelled 7 atom linker (yellow) and 9 atom linker (dark blue) when Met163 has not flipped and blocks the linker channel. The crystal structure of the ATP site binding fragment **8** is shown (green, 5CSP).

The linker was then further expanded to incorporate various hydrogen bonding groups including hydroxyl (**13**), amine (**14**), amides (**15**–**17**) and sulfonyl amide (**18**) moieties. The X-ray structure of compound **13** showed the trajectory of the linker was still away from the target region of the ATP site (Fig.7a and b). However, when an amide or sulfonyl amide were incorporated in this position or an amine with a four carbon linker (compounds **14**–**17**), the side chain of Met163 changed its conformation, turning into the ATP site (Fig.7a). This movement opened a small channel between the αD site and the ATP site for the linker, with the amide nitrogen forming a hydrogen bond to the backbone carbonyl of His160 and the amide stacking against His160 (Fig.7a). More importantly, the linker was now extending towards the desired region of the ATP site. This new channel effectively cut the corner between the αD site and the ATP site and would allow the use of shorter and more efficient linkers. Indeed, modelling of linkers now indicated that only a seven atom linker would be required to link the two fragments together which is an important improvement on the initially proposed 9 atom linker (Fig.7d, e and f).

Further elaboration of the linker led to molecules **19** and **20** that were suitable for linking with **8** (Fig.7c). The affinity of these extended compounds was determined by ITC, showing very similar K_d_ values of approximately 250 µM for both **19** and **20**. Therefore, despite making extensive contact with CK2α, the linker was not increasing the affinity for the protein, most likely due to its inherent flexibility leading to a greater entropic penalty upon binding. The ligand efficiency therefore decreased significantly to 0.23 and 0.19 for **19** and **20**, respectively.

#### 3.2.4 Linking strategy

**Fig. 8:**
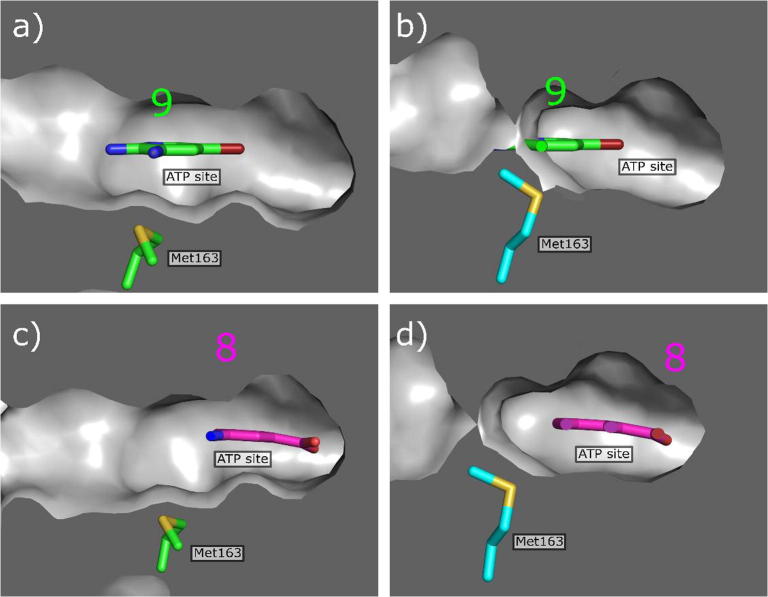
a) The crystal structure of **9**, a hinge binding fragment, bound to CK2α. Met163 (green) is in the down position where it blocks the channel between the αD site and the ATP site. b) The structure of **9** superimposed onto the structure of **19** where Met163 (blue) is flipped up and opens the channel between the αD site and the ATP site. Met163 would in this position clash with ZT0432. c) The crystal structure of **8** bound to CK2α. Met163 (green) is in the down position where it blocks the channel between the αD site and the ATP site. d) The structure of **8** superimposed onto the structure of **19** where Met163 (blue) is flipped up and opens the channel between the αD site and the ATP site. Met163 does not clash with **8**.

The information gained from the linker growth was then utilized in the linking of the optimised anchor **19** and the warhead **8** at the ATP site. Analysis of the molecules growing from the aD pocket also confirmed our selection of the ATP site fragment. Change in the conformation of the side chain of Met163, as induced by the optimal linker, changes the ATP pocket in the hinge region and would affect the interaction of fragments in this region. As the linkers seemed not to induce conformational changes in the other side of the ATP pocket, fragments interacting with Lys69 should be able to bind freely. Indeed, the superimposition of the crystal structure of **21**, a fragment from the Zenobia screen that interacts just with the hinge region of CK2α (Fig.8a), onto the structure of the ATP site when **19** binds to the αD site indicates that **21** would not bind simultaneously with the linker molecule **19** (Fig.8b). Whereas, when **8**, which interacts with only Lys69 (Fig.8c), is superimposed onto the structure of **19**, Met163 does not appear to prevent its binding (Fig.8d) (ATP_site_fragments_Met163_movement.pse).

**Fig. 9:**
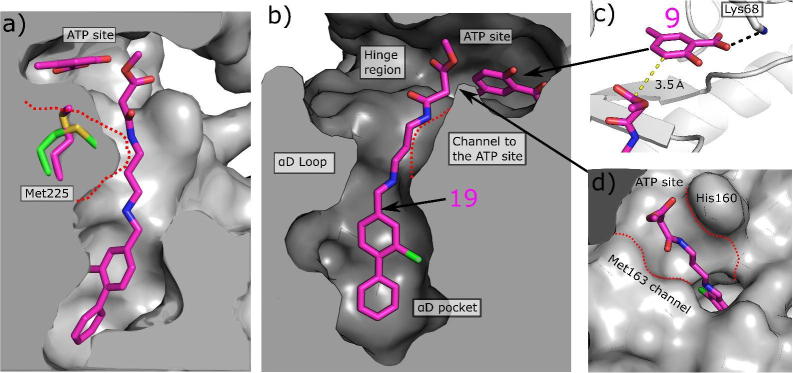
Optimisation of the linker between the αD pocket and the ATP site. a) and b) The structure of **19** and **9** (PDB: 5CU2), co-soaked into CK2α. The surface of the channel when fragment **7** was bound in the αD pocket (PDB: 5CSH) is indicated by the red dashed line. The position of Met163 in the apo form is shown (green). c) A more detailed view of the structure of **9** co-soaked with **19**, showing the location of the putative sites of linking (yellow dashed line); (d) The structure of **19** bound to CK2α showing the surface of the channel between the αD site and the ATP site formed by the movement of Met163.

To confirm this hypothesis, the ATP site fragments **8** and **9** were both co-soaked into CK2α with compound **19**. Unfortunately, the crystals containing **8** did not give data with high enough resolution to be able to fit the ligand. On the other hand, crystal structures of **9** with **19** showed that the benzoic acid core fragment at the opposite end of the ATP site did not prevent the movement of Met163 and thus the opening of the channel. The ester of the linker in **19** was slightly displaced compared to the structure without the fragment **9** as the end of the linker bound in close proximity to the fragment (Fig. 9) (Compound19.pse).

In addition, these structures confirmed that fragments that interacted with the hinge region would prevent the movement of Met163 and would therefore not be optimal for linking from the αD pocket. Moreover, the co-soaked structures also confirmed the results from the modelling studies that the best position to link to the benzoic acid core is the 3-position of the aromatic ring (Fig.8b). With these results in hand, fragment **8** was confirmed to be the best warhead to be connected to **19** as a molecule carrying the anchor and the linker.

### 3.3 CAM4066

**Fig. 10:**
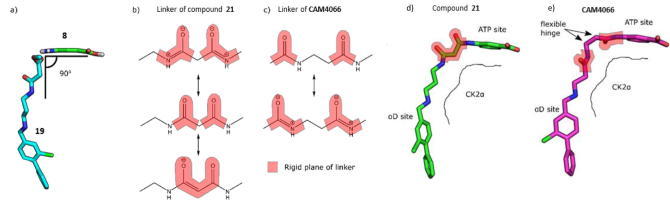
The flexibility of the 2 linked inhibitors. a) The ATP site fragment **8** and the linker **19** are out of plane with each other by approximately 90° which makes successful linking more challenging. b) The various resonance forms of **21**. The resonance forms impart rigidity to the linker in the position where flexibility is required. This may account for the reduced affinity. c) The various resonance forms of **CAM4066**. The resonance forms allow flexibility to the linker in the needed position. d) The binding conformation of **21** in CK2α. The highlighted areas are the rigid sections of the linker. e) The binding conformation of **CAM4066** in CK2α. The highlighted areas are the rigid sections of the linker.

The information gained from the linker optimisation process was then utilized in the final linking of **19** and **8**. Initially, two linked compounds were designed and synthesized (compound **21** and **CAM4066**). Both compounds contained a seven atom linker as this was the required length established through the optimisation and design process, however, the position of the amide groups within the linkers was varied. In order for compounds **19** and **8** to be linked together to provide the optimal inhibitor, the linker would have to bend by 90° as **8** and **19** are in different planes (Fig.10a). It was also predicted that the position of the amide groups in compound **21** conferred undesired rigidity to the linker (Fig.10b). Modelling suggested that **CAM4066**, an amide isomer of **21**, would be a valid alternative as it would be able to retain a hydrogen bond network with the channel and, more importantly, it would not contain rigid amides in the region of the linker where the greatest flexibility was required (Fig.10c). Both compounds were synthesized and co-crystal structures of both compounds bound to CK2α showed that they successfully linked the two sites (Fig.10d and e).

The affinity of **CAM4066** for CK2α was established to be significantly higher than that of **21**, therefore, the validation focused upon **CAM4066** (CAM4066.pse). The lower activity of **21** was attributed to the rigidity of the amide groups where the linker was required to be flexible (Fig. 10). As previously reported, although **CAM4066** was a potent CK2α inhibitor it had to be administered as a pro-drug, pre-**CAM4066**, in order to gain activity in cell based assays. This was probably due to the zwitterionic nature of **CAM4066**, which is often associated with poor cell permeability (Table 3).

**Table 3:** Structures and IC_50_ of the final linked compounds.

**Figure:**
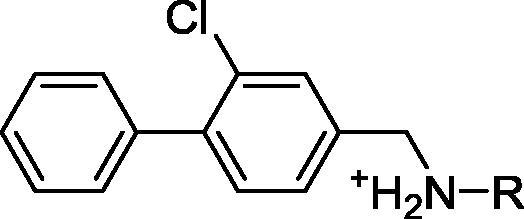

Compound	Structure	K_d_	IC_50_	PDB
**21**		1.64	n/a	5MO8
**CAM4066**		0.320	0.370	5CU4
**Pre-CAM4066**		n/a	n/a	N/D

**Fig. 11:**
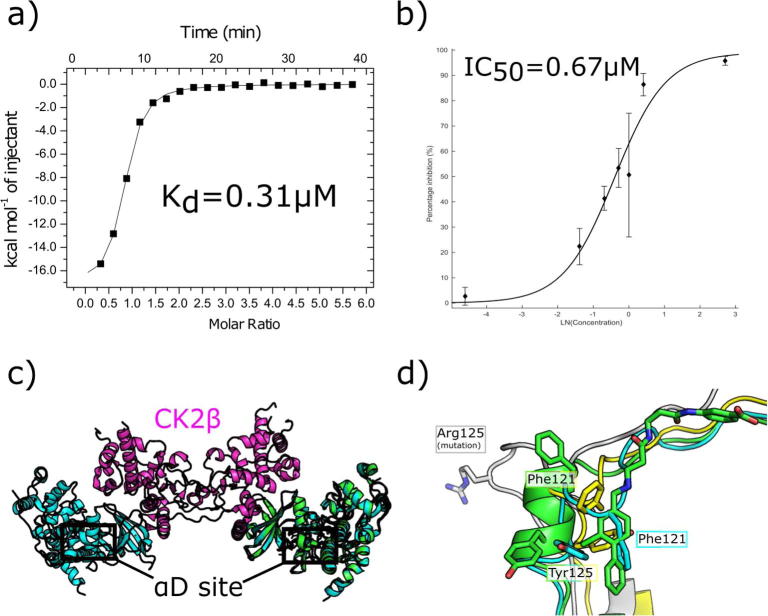
The inhibition of the CK2 holoenzyme by **CAM4066**. a) The isotherm of **CAM4066** binding to the CK2 holoenzyme. b) The inhibition of kinase activity of the CK2 holoenzyme by **CAM4066.** c) The structure of the CK2 holoenzyme, CK2α is shown in blue and CK2β is shown in purple (PDB: 4MD7)^30^ with the structure of **CAM4066** binding to CK2α, shown in green, (PDB: 5CU4)^22^ superimposed onto it. The position of the αD site is highlighted. d) A close up view of several conformations of the αD loop observed in the CK2 holoenzyme crystal structures with the structure of **CAM4066** bound to CK2α, shown in green, (PDB: 5CU4)^22^ superimposed on them. The closed conformation is shown in light blue (PDB: 4MD7)^30^ the partly open form in yellow (PDB: 1 JWH)^31^ and the open conformation, in which Tyr125 is mutated to Arg125 is shown in grey (4DGL)^32^.

**CAM4066** was then shown to be the most selective CK2α inhibitor discovered to date which validated the concept of developing inhibitors that bound in the novel αD site. The pro-drug form, pre-**CAM4066**, was shown to exhibit good activity against various cell lines and to inhibit the phosphorylation of various CK2α substrates confirming target engagement within the cell. The validation process has been reported in detail in our previous work which focused on the inhibition of the catalytic subunit CK2α.^22^ Further to this we have now confirmed that **CAM4066** also inhibits and binds to CK2α when it is in a complex with its regulatory domain CK2β. The CK2 holoenzyme was expressed and purified as described in the Supplementary materials and the affinity of CK2α for CK2β was found to be 14 nM, which is comparable with the previously reported value of 12.6 nM.^33^ The affinity of **CAM4066** for the complex was determined to be 0.31 µM by ITC (Fig.11a) and this value is similar to that reported for **CAM4066** and the isolated catalytic subunit of 0.32 µM. It was then confirmed that **CAM4066** also inhibits the kinase activity of the CK2 holoenzyme using the previously reported phosphorylation assay.^22^ This showed that **CAM4066** inhibited phosphorylation by the complex with an IC_50_ similar to that of CK2α (Fig.11b). The IC_50_ of **CAM4066** against the complex is 0.67 ± 0.29 µM whereas the IC_50_ against CK2α was 0.37 ± 0.06 µM. Although the IC_50_ against the complex was higher than the one of CK2α, the difference is not significant considering the error of the assay. This data confirms that the formation of the complex does not prevent the opening of the αD pocket. This conclusion was predicted from analysis of the various crystal structures of the CK2 complex superimposed on the structures of **CAM4066** bound to CK2α (Fig.11c and d). It can be clearly seen that the formation of the holoenzyme does not result in the formation of a protein–protein interface that would block the opening of the αD pocket (Fig.11c). Likewise, comparison of the various holoenzyme structures published indicates that formation of the holoenzyme complex does not result in a reduction in the flexibility of the αD loop which would prevent the opening of the αD pocket. As with the structures of CK2α both the closed (PDB: 4MD7)^30^ and partly open (PDB: 1JWH)^31^ conformations of the αD loop are observed in the wildtype holoenzyme complex structures indicating the loop retains its flexibility in the complex. It is also worthy of note that when Tyr125 is mutated to Arg (PDB: 4DGL)^32^ that the loop is observed in the open conformation, presumably because it is unfavourable for the Arginine to fill the αD pocket. This strengthens the conclusion that it is possible for the pocket to open in the holoenzyme as it indicates that the holoenzyme formation does not block the opening of the pocket or increase the rigidity of the loop.

## 4 Conclusions

In the present work we give a detailed description of fragment-based inhibitor development that utilized the unforeseen discovery of a novel binding pocket on CK2α. The αD site was discovered by the serendipitous binding of fragments in this new site. These fragments were first optimised to give a selective anchor in αD site that was then linked to a weakly binding fragment in the ATP site which acted as the warhead to provide the inhibition. In order to achieve the super additivity possible from linking fragments an extensive linker optimisation program was pursued to identify the length and composition of the linker. The resulting compound vindicated this strategy of carefully optimising the linker as the affinity of the final compound (320nM) was greater than could be expected from the combination of two fragments with 250 µM and >500 µM affinities. Although further work is required to improve this chemical tool into a lead candidate, the discovery of **CAM4066** has validated the concept of using the newly discovered αD site to achieve selectivity. Furthermore, it showed a case of successfully applying a fragment linking strategy which is known to be particularly challenging. *In-silico* molecular modelling was a remarkably beneficial tool as, together with X-ray, it provided a means to development a suitable linker to attach the high-affinity molecule lying in the αD pocket to the low-affinity fragment binding in the ATP site. Moreover, this work provided knowledge regarding the new cryptic pocket and the linker channel, showing potential for the development of a new class of CK2 inhibitors.

## A Supplementary data

Supplementary data 1 Data accessibility.
