# Supplementary material for: A fragment-based approach leading to the discovery of a novel binding site and the selective CK2 inhibitor CAM4066
Source: Bioorg Med Chem. 2017 Jul 1;25(13):3471–82. doi: 10.1016/j.bmc.2017.04.037 (PMC5587527; doi:10.1016/j.bmc.2017.04.037)
Supplement: Supplementary data 1 — Data accessibility. [file mmc1.docx]

**Supporting Information**

**A fragment-based approach leading to the discovery of a novel binding site and of the selective CK2 inhibitor CAM4066**

Claudia De Fusco, Paul Brear, Jessica Iegre, Kathy Hadje Georgiou, Hannah F. Sore, Marko Hyvӧnen*, David R. Spring*

**Contents**

[**SI_1 General experimental details** 3](#_Toc475886295)

[**SI_2 General Methods** 4](#_Toc475886296)

[**SI_3 Detailed experimental procedures and compound characterisation** 6](#_Toc475886297)

[**SI_4 Selected NMR spectra** 16](#_Toc475886298)

[**SI_5 Thermal shift results for 25 fragments** 40](#_Toc475886299)

[**SI_6 Expression and purification of the CK2 holoenzyme** 41](#_Toc475886300)

[**SI_7 ITC** 42](#_Toc475886301)

[**SI_8 Kinase assays** 42](#_Toc475886302)

[**SI_9 Crystallography** 43](#_Toc475886303)

# **SI_1 General experimental details**

All the docking studies herein reported were performed using Glide of the suite Maestro, produced by Schrodinger. The protein was prepared starting from the pdb file of the X-ray structures and using the PrepWinz feature of Maestro. To keep the conformation of the protein as close as possible to the X-ray structures, no modifications such as ‘cap termini’, ‘filling loops’ or ‘side chain’, ‘original hydrogen deletion’ were performed. Only the orientation of the water molecules was sampled and the ionization state of the protein was calculated at pH 7 ± 2. Ligands were prepared using the LigPrep functionality, using OPLS_2005 as the force field. All other parameters were left as default. A conformational search of the ligands was performed after ligand preparation. For the grid generation, the parent ligand of the crystal structure was used as the template and positional constraints were given to the benzylamine nitrogen of the ligand. All other parameters were left as default. Ligand input partial charges were used.

All reagents and solvents were used as received unless otherwise stated.

Compounds **1**, **2**, **8** and **9** were purchased from commercially available sources and used without further purification.

CH_2_Cl_2_, EtOAc, MeOH, MeCN and toluene was distilled from CaH_2_. Tetrahydrofuran (THF) was dried over Na wire and distilled from a mixture of LiAlH_4_ and CaH_2_ with triphenylmethane as the indicator. Et_2_O was distilled from a mixture of LiAlH_4_ and CaH_2_. Petroleum ether was distilled before use and refers to the fraction between 40-60 ˚C. All reactions were monitored by thin layer chromatography (TLC) using glass plates precoated with Merck silica gel 60 F254. Flash column chromatography was carried out using slurry-packed Merck 9385 Kieselgel 60 silica gel under a positive pressure of nitrogen. Semi-HPLC purification was performed on an Agilent 1260 Infinity system fitted with a Supelcosil ABZ+Plus column (250 mm x 21.2 mm, 5 μm) using linear gradient systems (solvent A: 0.1% (v/v) TFA in water, solvent B: 0.05% (v/v) TFA in acetonitrile) at a flow rate of 20 mL·min^-1^.

Nuclear magnetic resonance spectra (NMR) were recorded using an internal deuterium lock on Bruker DPX 400 (400MHz), Bruker Avance 400 QNP Ultrashield (400 MHz), Bruker Advance 500 BB ATM (500 MHz) and Bruker Avance 500 Cryo Ultrashield (500 MHz) spectrometers. Chemical shifts (δ) are referenced to the solvent residual peak and are quoted in ppm to the nearest 0.01 ppm for δ_H_ and to the nearest 0.1 ppm for δ_C_. Coupling constants (*J*) are reported in Hertz to the nearest 0.1 Hz. High resolution mass spectrometry (HRMS) was carried out with a Micromass Q-TOF or a Waters LCT Premier Mass Spectrometer using electrospray ionisation [ESI]. Melting points data were collected on a BÜCHI B-545 and are uncorrected. Infrared (IR) spectra were recorded on a Perkin-Elmer Spectrum One FT-IR spectrometer using a Diamant/KRS5 ATR. Selected absorption maxima (νmax) are reported in wavenumbers (cm^-1^).

# **SI_2 General Methods**

**General method a: Williamson ether synthesis**

To a solution of the phenol (1.0 equiv) in anhydrous *N,N*-dimethylformamide (0.22 M), under nitrogen, was added the alkyl halide (2.0 equiv) and potassium carbonate (2.0 equiv) and the reaction was heated to 140 °C for 18 hours. The reaction was allowed to cool to room temperature and partitioned between aqueous ammonium chloride and ethyl acetate. The organic layer was separated and washed with water (2×) and brine, then dried (MgSO_4_) and concentrated under reduced pressure. The crude residue was purified by column chromatography to yield the product.

**General method b: Benzonitrile reduction**

To a suspension of lithium aluminium hydride (2.0 equiv) in anhydrous diethyl ether (0.27 M), under nitrogen, was added aluminium chloride (2.0 equiv). The reaction mixture was cooled to 0 °C and stirred for 10 minutes. The reaction was allowed to warm to room temperature and the nitrile (1.0 equiv) was added portionwise. The reaction was stirred at room temperature for 30 minutes and then heated to 40 °C for 18 hours. The resultant suspension was allowed to cool to room temperature and ethyl acetate and saturated aqueous potassium sodium tartrate tetrahydrate were added and the mixture stirred for 1 hour. The reaction mixture was poured into 2M aqueous sodium carbonate and extracted with ethyl acetate (3×). The combined organic extracts were dried (MgSO_4_) and concentrated under reduced pressure to yield the desired product.

**General method c: Synthesis of hydrochloride salts**

To a solution of the amine (1.0 equiv) in anhydrous dichloromethane, under nitrogen, was added hydrochloric acid in diethyl ether (2 M, 10 equiv). The reaction was stirred for 1 hour and the precipitate was filtered and washed with cold diethyl ether. Purification by recrystallisation from ethanol or methanol yielded the hydrochloride salt as a crystalline solid.

**General method d: Phenol triflation**

To a solution of the corresponding phenol (1.0 equiv) in anhydrous dichloromethane (~ 0.3 M), under nitrogen, was added pyridine (3.0 equiv). The solution was cooled to 0 °C and trifluoromethanesulfonic anhydride (1.1 equiv) was added dropwise over 30 minutes. The reaction was allowed to warm to room temperature and stirred for 18 hours. Dichloromethane and excess trifluoromethansulfonic acid were removed under reduced pressure and the residue was diluted with water and extracted with ethyl acetate. The organic layer was washed with 10% aqueous hydrochloric acid, 5% aqueous sodium bicarbonate, brine and water, then dried (MgSO_4_) and concentrated under reduced pressure. The crude residue was purified by column chromatography to yield the desired product.

**General method e: Suzuki coupling**

The aryl triflate or bromide (1.5 equiv), the appropriate boronic acid (1.0 equiv), lithium chloride (1.8 equiv), anhydrous 1,2-dimethoxyethane (0.18 M) and 2 M aqueous sodium carbonate (1.6 equiv) were placed in a microwave tube. The reaction mixture was degassed by bubbling nitrogen through the solution for 5 minutes before the addition of tetrakis(triphenylphosphine)palladium(0) (6.25-25 mol%). The solution was degassed for a further 5 minutes and then heated to 135 °C under microwave irradiation for 1-2 hours. The reaction was allowed to cool to room temperature and then poured into water and extracted with ethyl acetate (3×). The combined organic extracts were washed with brine, dried (MgSO_4_), concentrated under reduced pressure and purified by column chromatography to yield the desired product.

**General method f: Reductive amination with free amine**

The benzaldehyde (1.0 equiv) and the amine (1.5 equiv) were combined in anhydrous 1,2 dichloroethane (0.05-0.29 M) under an atmosphere of nitrogen and stirred for 3 hours. Sodium triacetoxyborohydride (1.4 equiv) was added and the reaction was stirred at room temperature for 18 hours. The reaction mixture was poured into 2 M aqueous sodium carbonate and extracted with ethyl acetate (3×). The combined organic extracts were dried (MgSO_4_), concentrated under reduced pressure and purified by column chromatography to yield the desired product.

**General method g: Reductive amination with ammonium salts**

A solution of the ammonium salt (1.5 equiv) in methanol (0.28 M) was treated with Et_3_N (2.0 equiv) and aldehyde (1 equiv) and the mixture stirred at rt for 2 h. Sodium triacetoxyborohydride (1.4 equiv) was added in two portions with a 30 min interval and the reaction was stirred at rt for 18 h. The reaction mixture was poured into 2 M aqueous sodium carbonate and extracted with dichloromethane (3x). The combined organic extracts were dried (MgSO_4_), concentrated under reduced pressure and purified by column chromatography to yield the desired product.

**General method h: Hydrolysis of methyl ester**

To a solution of the methyl ester (1.0 equiv) in tetrahydrofuran (0.05 M) at 0 °C was added a 0.3 M aqueous lithium hydroxyde solution (3 equiv) and the mixture was stirred at rt until consumption of the starting material (~2 h). A 4 N hydrochloric acid solution in dioxane (3 equiv) was added and after 10 min the solvent was removed under reduced pressure.

# **S****I_3 Detailed experimental procedures and compound characterisation**

**3-Chloro-4-isopropoxybenzonitrile (3a)**: Prepared by general method **a** using 3-chloro-4-hydroxybenzonitrile (500 mg, 3.26 mmol), *N*,*N*-dimethylformamide (15 mL), 2-bromopropane (612 μL, 6.52 mmol) and potassium carbonate (901 mg, 6.52 mmol). The crude residue was purified by column chromatography (ethyl acetate: hexane 10:90) to provide the title compound as clear oil (538 mg, 84%). R*_f_* = 0.33 [ethyl acetate: hexane (20:80)]. δ_H/_ppm (400 MHz, CDCl_3_): 7.65 (1H, d, *J* = 2.1 Hz), 7.51 (1H, dd, *J* = 8.6 and 2.1 Hz), 6.95 (1H, d, *J* = 8.6 Hz), 4.66 (1H, sept, *J* = 6.1 Hz), 1.42 (6H, d, *J* = 6.1 Hz). δ_C_/ppm (101 MHz, CDCl_3_): 157.5, 134.0, 132.3, 124.7, 118.2, 114.4, 104.3, 72.4, 21.9. ν_max_/cm^-1^: 2983, 2228, 1595, 1493. HRMS (ESI+) *m/z* found [M+Na]^+^ 218.0341, C_10_H_10_NOClNa^+^ required 218.0343.

(**3-Chloro-4-isopropoxyphenyl)methanaminium chloride (3)**: Prepared by general method **b** using lithium aluminium hydride (92.0 mg, 2.42 mmol), diethyl ether (5 mL), aluminium chloride (320 mg, 2.40 mmol) and **3a** (235 mg, 1.20 mmol). The crude amine (182 mg, 0.911 mmol) was converted to the hydrochloride salt using hydrochloric acid (4.5 mL, 9.1 mmol, 2 M in diethyl ether) according to general method **c** to provide the title compound as an off-white crystalline solid (149 mg, 52%). M.p. = 225-226 °C. δ_H_/ppm (400 MHz, DMSO-d_6_): 8.39 (3H, br s), 7.60 (1H, d, *J* = 2.1 Hz), 7.40 (1H, dd, *J* = 8.6 and 2.1 Hz), 7.20 (1H, d, *J* = 8.6 Hz), 4.69 (1H, sept, *J* = 6.0 Hz), 3.94 (2H, s), 1.28 (6H, d, *J* = 6.0 Hz). δ_C/_ppm (126 MHz, DMSO-d_6_): 153.0, 130.9, 129.2, 127.2, 122.2, 115.5, 71.2, 41.1, 21.7. ν_max_/cm^-1^: 3640, 2915, 1601, 1504. HRMS (ESI+) *m/z* found [M+H]^+^ 200.0831, C_10_H_15_NOCl^+^ required 200.0837.

**3-Chloro-4-propoxybenzonitrile (4a)**: Prepared by general method **a** using 3-chloro-4-hydroxybenzonitrile (500 mg, 3.26 mmol), *N*,*N*-dimethylformamide (15 mL), 1-bromopropane (590 μl, 6.52 mmol) and potassium carbonate (901 mg, 6.52 mmol). The crude residue was purified by column chromatography (ethyl acetate: hexane 10:90) to provide the title compound as clear oil (545 mg, 85%). R_f_ = 0.50 [ethyl acetate: hexane (20:80)]. δ_H_/ppm (400 MHz, CDCl_3_): 7.65 (1H, d, *J* = 2.1 Hz), 7.52 (1H, dd, *J* = 8.6 and 2.1 Hz), 6.95 (1H, d, *J* = 8.6 Hz), 4.05 (2H, t, *J* = 6.4 Hz), 2.00-1.82 (2H, m), 1.08 (3H, t, *J* = 7.4 Hz). δ_C_/ppm (101 MHz, CDCl_3_): 158.4, 133.7, 132.5, 124.0, 118.2, 113.1, 104.5, 71.1, 22.4, 10.5. ν_max_/cm^-1^: 2969, 2228, 1597, 1498. HRMS (ESI+) *m/z* found [M+H]^+^ 196.0517, C_10_H_11_NOCl^+^ required 196.0524.

**(3-Chloro-4-propoxyphenyl)methanaminium chloride (4)**: Prepared by general method **b** using lithium aluminium hydride (117 mg, 3.07 mmol), diethyl ether (6 mL), aluminium chloride (408 mg, 3.07 mmol) and **4a** (300 mg, 1.53 mmol). The crude amine (285 mg, 1.42 mmol) was converted to the hydrochloride salt using hydrochloric acid (7.1 mL, 14 mmol, 2 M in diethyl ether) according to general method **c** to provide the title compound as an off-white crystalline solid (222 mg, 61%). M.p. = 246-249 °C. δ_H_/ppm (500 MHz, DMSO-d_6_): 8.37 (3H, br s), 7.60 (1H, d, J = 2.2 Hz), 7.41 (1H, dd, *J* = 8.5 and 2.2 Hz), 7.17 (1H, d, *J* = 8.5 Hz), 4.03 (2H, t, *J* = 6.4 Hz), 3.95 (2H, s), 1.81-1.68 (2H, m), 0.99 (3H, t, *J* = 7.4 Hz). δ_C_/ppm (126 MHz, DMSO-d_6_): 154.4, 131.2, 129.8, 127.5, 121.6, 114.1, 70.5, 41.6, 22.3, 10.8. ν_max_/cm^-1^: 2963, 1610, 1506. HRMS (ESI+) *m/z* found [M+H]^+^ 200.0832, C_10_H_15_NOCl^+^ required 200.0837.

**3-Chloro-4-(cyclopentyloxy)benzonitrile (5a)**: Prepared by general method **a** using 3-chloro-4-hydroxybenzonitrile (500 mg, 3.26 mmol), *N*,*N*-dimethylformamide (15 mL), bromocyclopentane (700 μL, 6.52 mmol) and potassium carbonate (901 mg, 6.52 mmol). The crude residue was purified by column chromatography (eluting gradient ethyl acetate: hexane 1:9 to 2:8). The title compound was obtained as white viscous oil (642 mg, 89%). R*_f_* = 0.60 [ethyl acetate: hexane (20:80)]. δ_H_/ppm (500 MHz, CDCl_3_): 7.63 (1H, d, *J* = 2.1 Hz), 7.50 (1H, dd, *J* = 8.6 and 2.1 Hz), 6.95 (1H, d, *J* = 8.6 Hz), 4.89-4.84 (1H, m), 2.00-1.78 (6H, m), 1.73-1.60 (2H, m). δ_C_/ppm (126 MHz, CDCl_3_): 157.6, 133.8, 132.3, 124.5, 118.3, 114.3, 104.1, 81.5, 32.9, 24.1. ν_max_/cm^-1^: 2963, 2227, 1595, 1493. HRMS (ESI+) *m/z* found [M+Na]^+^ 244.0496, C_12_H_12_NOClNa^+^ required 244.0500.

**(3-Chloro-4-(cyclopentyloxy)phenyl)methanaminium chloride (5)**: Prepared by general method **b** using lithium aluminium hydride (103 mg, 2.71 mmol), diethyl ether (5 mL), aluminium chloride (361 mg, 2.71 mmol) and **5a** (300 mg, 1.35 mmol). The crude amine (230 mg, 1.02 mmol) was converted to the hydrochloride salt using hydrochloric acid (5.1 mL, 10 mmol, 2 M in diethyl ether) according to general method **c**. The precipitate was recrystallised from ethanol to yield the title compound as an off-white crystalline solid (38.8 mg, 11%). M.p. = 240-243 °C. δ_H_/ppm (500 MHz, d_6_-DMSO): 8.32 (3H, br s), 7.58 (1H, d, J = 2.2 Hz), 7.39 (1H, dd, *J* = 8.6 and 2.2 Hz), 7.18 (1H, d, *J* = 8.6 Hz), 4.96-4.90 (1H, m), 3.94 (2H, s), 1.99-1.82 (2H, m), 1.80-1.66 (4H, m) 1.66-1.52 (2H, m). δ_C/_ppm (126 MHz, DMSO-d_6_): 153.1, 130.8, 129.2, 126.9, 121.9, 115.1, 80.2, 41.2, 32.2, 23.5. ν_max_/cm^-1^: 2913, 1601, 1504. HRMS (ESI+) *m/z* found [M+H]^+^ 226.0984, C_12_H_17_NOCl^+^ required 226.0993.

**2-Chloro-4-cyanophenyl trifluoromethanesulfonate^[[1]](#footnote-1)^ (6a):** Prepared following general method **d** using 3-chloro-4-hydroxybenzonitrile (2.00 g, 13.0 mmol), anhydrous dichloromethane (40 mL), pyridine (3.20 mL, 39.6 mmol), trifluoromethanesulfonic anhydride (2.40 mL, 14.3 mmol). The crude residue was purified by column chromatography (ethyl acetate: hexane 5:95) to provide the title compound as a white solid (3.37 g, 91%). R*_f_* = 0.68 [ethyl acetate: hexane (20:80)]. M.p. = 61-62 °C, δ_H/ppm_ (500 MHz, CDCl_3_): 7.86 (1H, d, *J* = 2.0 Hz), 7.68 (1H, dd, *J* = 8.6 and 2.0 Hz), 7.51 (1H, d, *J* = 8.6 Hz). δ_F_/ppm (376 MHz, CDCl_3_): -73.08 (3F, s). δ_C_/ppm (126 MHz, CDCl_3_): 148.7, 135.0, 132.4, 129.0, 124.3, 118.7 (q, *J* = 320.8 Hz), 116.1, 113.9. ν_max_/cm^-1^: 3076, 2245, 1575, 1478. LCMS R*_t_* = 1.56 min, [M-H]^-^ 284.0.

**3-Chloro-4-(furan-3-yl)benzonitrile (6b):** Prepared by general method **e** using **6a** (154 mg, 0.539 mmol), 3-furanylboronic acid (90.0 mg, 0.804 mmol), lithium chloride (28 mg, 0.66 mmol), 1,2-dimethoxyethane (2.9 mL), 2 M aqueous sodium carbonate (61 mg, 0.58 mmol) and tetrakis(triphenylphosphine)palladium(0) (104 mg, 0.0900 mmol). The crude residue was purified by column chromatography (ethyl acetate: hexane 10:90) to provide the title compound as a white solid (77.1 mg, 70%). R*_f_* = 0.86 [ethyl acetate: hexane (50:50)]. M.p. = 103-104 °C. δ_H_/ppm (500 MHz, CDCl_3_): 7.98 (1H, dd, *J* = 1.5 and 0.9 Hz), 7.75 (1H, dd, *J* = 1.4 and 0.6 Hz), 7.77-7.57 (2H, m), 7.54-7.53 (1H, m), 6.75 (1H, dd, *J* = 1.9 and 0.9 Hz). δ_C_/ppm (126 MHz, CDCl_3_): 143.4, 142.5, 136.5, 134.0, 133.1, 130.6, 130.5, 122.4, 117.6, 111.7, 110.6. ν_max_/cm^-1^: 3121, 2233, 1601, 1519. HRMS no molecular ion found.

**(3-Chloro-4-(furan-3-yl)phenyl)methanaminium chloride (6):** Prepared by general method **b** using lithium aluminium hydride (25 mg, 0.65 mmol), diethyl ether (2.5 mL), aluminium chloride (87 mg, 0.65 mmol) and **6b** (66.4 mg, 0.326 mmol). The crude amine (63.8 mg, 0.307 mmol) was converted to the hydrochloride salt using hydrochloric acid (1.50 mL, 3.07 mmol, 2 M in diethyl ether) according to general method **c** to yield the title compound as an off-white crystalline solid (48.5 mg, 62%). M.p. = 255-257 °C. δ_H_/ppm (500 MHz, DMSO-d_6_): 8.50 (3H, br s), 8.17 (1H, dd, *J* = 1.5 and 0.9 Hz), 7.81 (1H, t, *J* = 1.7 Hz), 7.72 (1H, d, *J* = 1.8 Hz), 7.65 (1H, d, *J* = 8.0 Hz), 7.51 (1H, dd*, J* = 8.0 and 1.8 Hz), 6.94 (1H, dd*, J* = 1.9 and 0.9 Hz), 4.05 (2H, s). δ_C_/ppm (126 MHz, DMSO-d_6_): 143.5, 141.7, 134.7, 130.9, 130.7, 130.6, 130.4, 128.1, 122.4, 110.9, 41.2. ν_max_/cm^-1^: 3678, 2965, 1598, 1523. HRMS (ESI+) *m/z* found [M+H]^+^ 208.0522, C_11_H_11_NOCl^+^ required 208.0524.

**2-Chloro-[1,1'-biphenyl]-4-carbonitrile (7a):** Prepared by general method **e** using **6a** (75 mg, 0.26 mmol), phenylboronic acid (21.3 mg, 0.175 mmol), lithium chloride (13 mg, 0.31 mmol), 1,2-dimethoxyethane (1.5 mL), 2M aqueous sodium carbonate (26 mg, 0.25 mmol) and tetrakis(triphenylphosphine)-palladium(0) (13 mg, 0.011 mmol). The crude residue was purified by column chromatography (ethyl acetate: hexane 5:95) to provide the title compound as a white solid (27.3 mg, 73%). R*_f_* = 0.63 [ethyl acetate: hexane (20:80)]. M.p. = 78-79 °C. δ_H_/ppm (500 MHz, DMSO-d_6_): 8.18 (1H, d, *J* = 1.6 Hz), 7.91 (1H, dd, *J* = 8.0 and 1.6 Hz), 7.62 (1H, d, *J* = 8.0 Hz), 7.54-7.44 (5H, m). δ_C_/ppm (126 MHz, DMSO-d_6_): 144.6, 137.2, 133.4, 132.4, 132.3, 131.3, 129.0, 128.6, 128.4, 117.5, 111.9. ν_max_/cm^-1^: 3065, 2230, 1595, 1501, 1474. HRMS (ESI+) *m/z* found [M+H]^+^ 214.0412, C_13_H_9_NCl^+^ required 214.0418.

**(2-Chloro-[1,1'-biphenyl]-4-yl)methanaminium chloride (7):** Prepared by general method **b** using lithium aluminium hydride (11 mg, 0.28 mmol), diethyl ether (1 mL), aluminium chloride (37 mg, 0.28 mmol) and **7a** (30 mg, 0.14 mmol). The crude amine (24 mg, 0.11 mmol) was converted to the hydrochloride salt using hydrochloric acid (0.60 mL, 1.2 mmol, 2 M in diethyl ether) according to general method **c** to provide the title compound as a white crystalline solid (17.0 mg, 48%). M.p. = 270-273 °C. δ_H_/ppm (500 MHz, DMSO-d_6_): 8.45 (3H, br s), 7.75 (1H, d, *J* = 1.7 Hz), 7.54 (1H, dd, *J* = 7.9 and 1.7 Hz), 7.51-7.40 (6H, m), 4.09 (2H, s). δ_C_/ppm (126 MHz, DMSO-d_6_): 139.8, 138.2, 135.3, 131.6, 131.2, 130.3, 129.1, 128.3, 128.1, 128.0, 41.3. ν_max_/cm^-1^: 3420, 2913, 1607, 1514. HRMS (ESI+) *m/z* found [M+H]^+^ 218.0729, C_13_H_13_NCl^+^ required 218.0737.

**2-chloro-4-formylphenyl trifluoromethanesulfonate^[[2]](#footnote-2)^ (10a):** Prepared by general method **d** using 3-chloro-4-hydroxybenzaldehyde (2.00 g, 12.7 mmol) in anhydrous dichloromethane (120 mL), pyridine (1.65 mL, 20.4 mmol) and trifluoromethanesulfonic anhydride (2.80 mL, 16.6 mmol).The crude residue was purified by column chromatography (eluent ethyl acetate: hexane 5:95) to yield the desired product as a white solid (3.61 g, 98%). R*_f_* = 0.43 [ethyl acetate: hexane (20:80)]. M.p. = 102-104 °C. δ_H_ /ppm (500 MHz, DMSO-d_6_): 10.02 (1H, s), 8.29 (1H, d, *J* = 1.9 Hz), 8.06 (1H, dd, *J* = 8.5 and 2.0 Hz), 7.90 (1H, d, *J* = 8.5 Hz). δ_F_ /ppm (376 MHz, CDCl3): -73.23 (3F, s). δ_C_ /ppm (101 MHz, DMSO-d_6_): 190.9, 148.2, 137.1, 132.1, 130.1, 127.0, 124.5, 118.1 (q, *J* = 320.6 Hz). ν_max_ /cm^-1^: 2851, 1692, 1594. LCMS R*_t_* = 2.50 min, [M]^-^ 287.9.

**2-Chloro-[1,1'-biphenyl]-4-carbaldehyde (10b):** Prepared by general method **e** using **10a** (341 mg, 1.18 mmol), phenylboronic acid (158 mg, 1.30 mmol), lithium chloride (61 mg, 1.4 mmol), 1,2-dimethoxyethane (6.3 mL), 2M aqueous sodium carbonate (133 mg, 1.25 mmol) and tetrakis(triphenylphosphine)palladium(0) (227 mg, 0.196 mmol). The crude residue was purified by column chromatography (eluting gradient ethyl acetate: hexane 2:98 to 20:80) to provide the title compound as a white solid (139 mg, 49%). R*_f_* = 0.73 [ethyl acetate: hexane (20:80)]. M.p. = 96-98 °C. δ_H_/ppm (400 MHz, CDCl_3_): 10.02 (1H, s), 7.99 (1H, d, *J* = 1.6 Hz), 7.83 (1H, dd, *J* = 7.8 and 1.6 Hz), 7.53 (1H, d, *J* = 7.8 Hz), 7.50-7.40 (5H, m). δ_C/_ppm (101 MHz, CDCl_3_): 190.8, 146.5, 138.3, 136.6, 133.9, 132.2, 131.2, 129.3, 128.6, 128.4, 128.0. ν_max_/cm^-1^: 2835, 1697, 1597. HRMS (ESI+) *m/z* found [M+H]^+^ 217.0414, C_13_H_10_OCl^+^ required 217.0420.

**1-(2-Chloro-[1,1'-biphenyl]-4-yl)-*N*-methylmethanaminium chloride (10):** Prepared by general method **f** using aldehyde **10b** (100 mg, 0.460 mmol), methylamine (8 M in ethanol, 86.5 μL, 0.692 mmol), 1,2-dichloroethane (1.65 mL) and sodium triacetoxyborohydride (137 mg, 0.646 mmol). The crude residue was purified by column chromatography (eluting gradient CH_2_Cl_2_:MeOH 100:0 to 95:5, R*_f_* = 0.23, CH_2_Cl_2_:MeOH 90:10) to give the free amine (84.3 mg, 78%). The amine (43.0 mg, 0.186 mmol) was then converted to the hydrochloride salt according general method **c** using hydrochloric acid (0.928 mL, 2 M in diethyl ether) to provide the title compound as a white solid (41.3 mg, 83%). M.p. = 225-227 °C. δ_H_/ppm (500 MHz, DMSO-d_6_): 9.23 (2H, br s), 7.78 (1H, d, *J* = 1.5 Hz), 7.57 (1H, dd, *J* = 7.9 and 1.7 Hz), 7.51 – 7.46 (3H, m), 7.43 (3H, tdt, *J* = 4.6, 3.0 and 1.4 Hz), 4.17 (2H, s), 2.57 (3H, s). δ_C_/ppm (126 MHz, DMSO-d_6_): 140.3, 138.1, 133.3, 131.7, 131.4, 131.2, 129.2, 129.1, 128.4, 128.0, 50.2, 32.2. ν_max_/cm^-1^: 2925, 2769, 2709, 1442, 1029, 764, 699. HRMS (ESI+) m/z found [M+H]^+^ 232.0867, C_14_H_15_ClN^+^ required 232.0893.

***N*-((2-chloro-[1,1'-biphenyl]-4-yl)methyl)ethanaminium chloride (11):** Prepared by general method **g** using aldehyde **10b** (100 mg, 0.460 mmol), ethylamine hydrochloride (56.4 mg, 0.692 mmol), methanol (1.65 mL), Et_3_N (96.5 μL, 0.692 mmol) and sodium triacetoxyborohydride (137 mg, 0.646 mmol). The crude residue was purified by column chromatography (eluent CH_2_Cl_2_:MeOH 98:2, R*_f_* = 0.37, CH_2_Cl_2_:MeOH 90:10) to give the free amine (92.0 mg, 81%). The amine (90.0 mg, 0.366 mmol) was then converted to the hydrochloride salt according general method **c** using hydrochloric acid (1.83 mL, 2 M in diethyl ether) to provide the title compound as a white solid (90.0 mg, 86%). M.p. =222-223 °C. δ_H_/ppm (500 MHz, DMSO-d_6_): 9.16 (2H, br s), 7.80 (1H, d, *J* = 1.6 Hz), 7.58 (1H, dd, *J* = 7.9 and 1.7 Hz), 7.52 – 7.47 (3H, m), 7.46 – 7.38 (3H, m), 4.18 (2H, s), 2.98 (2H, d, *J* = 6.6 Hz), 1.24 (3H, t, *J* = 7.3 Hz). δ_C_/ppm (126 MHz, DMSO-d_6_): 140.2, 138.1, 133.4, 131.7, 131.3, 131.3, 129.2, 129.1, 128.4, 128.0, 48.5, 41.9, 11.0. ν_max_/cm^-1^: 2945, 2785, 1445, 764, 699. HRMS (ESI+) m/z found [M+H]^+^ 246.1030, C_15_H_17_ClN^+^ required 246.1049.

***N*-((2-chloro-[1,1'-biphenyl]-4-yl)methyl)propan-1-aminium chloride (12):** Prepared by general method **f** using aldehyde **10b** (100 mg, 0.460 mmol), propylamine (56.9 μL, 0.692 mmol), 1,2-dichloroethane (1.65 mL) and sodium triacetoxyborohydride (137 mg, 0.646 mmol). The crude residue was purified by column chromatography (eluent CH_2_Cl_2_:MeOH 98:2, R*_f_* = 0.23, CH_2_Cl_2_:MeOH 90:10) to give the free amine (54.0 mg, 45%). The amine (54.0 mg, 0.208 mmol) was then converted to the hydrochloride salt according general method **c** using hydrochloric acid (1.04 mL, 2 M in diethyl ether) to provide the title compound as a white solid (52.1 mg, 85%). M.p. = 223-225 °C. δ_H_/ppm (500 MHz, DMSO-d_6_): 9.22 (2H, br s), 7.82 (1H, d, *J* = 1.5 Hz), 7.60 (1H, dd, *J* = 7.9 and 1.6 Hz), 7.51 – 7.46 (3H, m), 7.45 – 7.40 (3H, m), 4.18 (2H, t, *J* = 5.6 Hz), 2.88 (2H, q, *J* = 11.7 Hz), 1.68 (2H, dq, *J* = 15.0 and 7.5 Hz), 0.92 (3H, t, *J* = 7.5 Hz). δ_C_/ppm (126 MHz, DMSO-d_6_): 140.2, 138.1, 133.4, 131.7, 131.4, 131.3, 129.2, 129.2, 128.4, 128.0, 48.9, 48.3, 19.0, 11.0. ν_max_/cm^-1^: 2934, 2766, 1442, 762, 690. HRMS (ESI+) m/z found [M+H]^+^ 260.1189, C_16_H_19_ClN^+^ required 260.1206.

***N*-((2-chloro-[1,1'-biphenyl]-4-yl)methyl)-3-hydroxypropan-1-aminium chloride (13):** Prepared by general method **f** using aldehyde **10b** (26.0 mg, 0.120 mmol), 3-amino-1-propanol (13.8 μL, 0.180 mmol), 1,2-dichloroethane (0.430 mL) and sodium triacetoxyborohydride (35.7 mg, 0.168 mmol). The crude residue was purified by column chromatography (eluting gradient CH_2_Cl_2_:MeOH 95:5 to 90:10, R*_f_* = 0.12, CH_2_Cl_2_:MeOH 90:10) to give the free amine (28.9 mg, 87%). The amine (25.0 mg, 0.0906 mmol) was then converted to the hydrochloride salt according general method **c** using hydrochloric acid (0.453 mL, 2 M in diethyl ether) to provide the title compound as a white solid (24.9 mg, 89%). M.p. = 175-177 °C. δ_H_/ppm (400 MHz, DMSO-d_6_): 9.19 (2H, br s), 7.82 (1H, d, *J* = 1.6 Hz), 7.60 (1H, dd, *J* = 7.9 and 1.7 Hz), 7.56 – 7.46 (3H, m), 7.46 – 7.37 (3H, m), 4.77 (1H, br s), 4.20 (2H, br s), 3.49 (2H, t, *J* = 5.7 Hz), 3.13 – 2.87 (2H, m), 1.91 – 1.74 (2H, m). δ_C_/ppm (126 MHz, DMSO-d_6_): 140.2, 138.1, 133.3, 131.7, 131.4, 131.3, 129.2, 129.2, 128.3, 128.0, 57.9, 49.0, 44.6, 28.7. ν_max_/cm^-1^: 3310, 2950, 2756, 1673, 1444, 1059, 698. HRMS (ESI+) *m/z* found [M+H]^+^ 276.1144, C_16_H_19_NOCl^+^ required 276.1155.

***N*^1^-((2-chloro-[1,1'-biphenyl]-4-yl)methyl)butane-1,4-diaminium chloride (14):** Prepared by general method **f** using aldehyde **10b** (21.7 mg, 0.100 mmol), *N*-Boc-1,4- butanediamine (28.2 mg, 0.150 mmol), 1,2-dichloroethane (0.360 mL) and sodium triacetoxyborohydride (29.7 mg, 0.140 mmol). The crude residue was purified by column chromatography eluting with 98:2 CH_2_Cl_2_/MeOH (R*_f_* = 0.41, CH_2_Cl_2_/MeOH 90:10) to give the Boc-protected intermediate (22.3 mg, 57%). This material (20.0 mg, 0.0514 mmol) was then converted to the hydrochloride salt according general method **c** using hydrochloric acid (0.257 mL, 2 M in diethyl ether) to yield the product as a white solid (19.1 mg, 92%). M.p. = 276-278 °C. δ_H_ /ppm (500 MHz, DMSO-d_6_): 9.44 (2H, s), 7.98 (3H, s), 7.84 (1H, d, *J* = 1.7 Hz), 7.62 (1H, dd, *J* = 7.9 and 1.7 Hz), 7.56 – 7.46 (3H, m), 7.46 – 7.40 (3H, m), 4.18 (2H, s), 2.95 (2H, br s), 2.80 (2H, br s), 1.74 (2H, dt, *J* = 15.1 and 7.5 Hz), 1.63 (2H, dt, *J* = 15.1 and 7.5 Hz). δ_C_ /ppm (126 MHz, DMSO-d_6_): 140.2, 138.1, 133.3, 131.6, 131.4, 131.3, 129.2, 129.1, 128.3, 128.0, 48.9, 45.8, 38.1, 24.1, 22.4. ν_max_ /cm^-1^: 2918, 1529, 1446, 764, 698. HRMS (ESI+) m/z found [M+H]^+^ 289.1468, C_17_H_22_ClN_2_ required 289.1472.

**3-Acetamido-*N*-((2-chloro-[1,1'-biphenyl]-4-yl)methyl)propan-1-aminium chloride (15):** Prepared by general method **f** using aldehyde **10b** (65.0 mg, 0.300 mmol), *N*-(3-aminopropyl)acetamide (52.3 mg, 0.450 mmol), 1,2-dichloroethane (1.07 mL) and sodium triacetoxyborohydride (89.0 mg, 0.420 mmol). The crude residue was purified by column chromatography (eluting gradient CH_2_Cl_2_:MeOH 95:5 to 90:10, R*_f_* = 0.23, CH_2_Cl_2_/MeOH 9:1) to give the free amine (64.3 mg, 68% yield). The amine (54.0 mg, 0.170 mmol) was then converted to the hydrochloric salt according general method **c** using hydrochloride acid (0.852 mL, 2 M in diethyl ether) to provide the title compound as a white solid (52.4 mg, 87%). M.p. = 188-190 °C. δ_H_/ppm (400 MHz, DMSO-d_6_): δ 9.53 (2H, s), 8.15 (1H, t, *J* = 5.3 Hz), 7.85 (1H, d, *J* = 1.6 Hz), 7.63 (1H, dd, *J* = 7.9 and 1.6 Hz), 7.51 – 7.45 (3H, m), 7.44 – 7.40 (3H, m), 4.17 (2H, t, *J* = 5.8 Hz), 3.11 (2H, q, *J* = 6.4 Hz), 2.91 (2H, t, *J* = 13.4 Hz), 1.94 – 1.76 (5H, m). δ_C_/ppm (101 MHz, DMSO-d_6_): 169.6, 140.2, 138.2, 133.4, 131.6, 131.4, 131.2, 129.3, 129.2, 128.3, 128.0, 48.9, 44.6, 35.7, 25.9, 22.6. ν_max/_cm^-1^: 3290, 2942, 276, 1642, 1551, 1443, 763, 698. HRMS (ESI+) *m/z* found [M+H]^+^ 317.1408, C_18_H_22_N_2_OCl^+^ required 317.1421.

**3-Amino-*N*-((2-chloro-[1,1'-biphenyl]-4-yl)methyl)-3-oxopropan-1-aminium chloride (16):** Prepared by general method **g** using aldehyde **10b** (21.7 mg, 0.100 mmol), 3-aminopropanamide hydrochloride (18.7 mg, 0.150 mmol), MeOH (0.360 mL), Et_3_N (27.8 μL, 0.200 mmol) and sodium triacetoxyborohydride (29.7 mg, 0.140 mmol). The crude residue was purified by column chromatography (eluent CH_2_Cl_2_/MeOH 98:2, R*_f_* = 0.34, CH_2_Cl_2_/MeOH 9:1) to give the free amine (26.2 mg, 90%). The amine (20.0 mg, 0.0693 mmol) was then converted to the hydrochloric salt according general method **c** using hydrochloric acid (0.346 mL, 2 M in diethyl ether) to provide the title compound as a white solid (22.8 mg, 78%). M.p. = 240-242 °C. δ_H_/ppm (500 MHz, DMSO-d_6_): 9.30 (2H, br s), 7.82 (1H, d, *J* = 1.6 Hz), 7.62 (1H, br s), 7.60 (1H, dd, *J* = 7.9 and 1.7 Hz), 7.55 – 7.45 (3H, m), 7.45 – 7.24 (3H, m), 7.09 (1H, s), 4.20 (2H, t, *J* = 5.0 Hz), 3.14 – 3.03 (2H, m), 2.59 (2H, t, *J* = 7.3 Hz). δ_C/ppm_ (126 MHz, DMSO-d_6_): 171.2, 140.2, 138.1, 133.2, 131.7, 131.4, 131.3, 129.3, 129.2, 128.4, 128.0, 49.0, 42.7, 30.7. ν_max_/cm^-1^: 2903, 2743, 1344, 1672, 1445, 1077, 764, 698. HRMS (ESI+) m/z found [M+H]^+^ 289.1082, C_16_H_18_ClN_2_O^+^ required 289.1108.

**2-Acetamido-*N*-((2-chloro-[1,1'-biphenyl]-4-yl)methyl)ethan-1-aminium chloride (17):** Prepared by general method **f** using aldehyde **10b** (43.3 mg, 0.200 mmol), *N*-(2-aminoethyl)acetamide (28.7 μL, 0.300 mmol), 1,2-dichloroethane (0.710 mL) and sodium triacetoxyborohydride (59.3 mg, 0.280 mmol). The crude residue was purified by column chromatography (eluting gradient CH_2_Cl_2_/MeOH 98:2 to 90:10, R*_f_* = 0.24, CH_2_Cl_2_/MeOH 90:10) to give the free amine (42.0 mg, 69%). The amine (40.0 mg, 0.155 mmol) was then converted to the hydrochloride salt according general method **c** using hydrochloric acid (0.776 mL, 2 M in diethyl ether) to yield the product as a white solid (40.2 mg, 85%). M.p. = 171-172 °C. δ_H_ /ppm (400 MHz, DMSO-d_6_): 9.59 (2H, s), 8.31 (1H, t, *J* = 5.4 Hz), 7.86 (1H, d, *J* = 1.4 Hz), 7.63 (1H, dd, *J* = 7.9 and 1.5 Hz), 7.56 – 7.45 (3H, m), 7.42 (3H, dd, *J* = 7.2 and 4.2 Hz), 4.21 (2H, t, *J* = 5.7 Hz), 3.42 (2H, app q, *J* = 6.0 Hz), 3.19 – 2.82 (2H, m), 1.84 (3H, s) . δ_C_ /ppm (101 MHz, DMSO-d_6_): 170.0, 140.2, 138.1, 133.2, 131.6, 131.5, 131.3, 129.3, 129.2, 128.3, 128.0, 48.8, 46.2, 35.1, 22.7. ν_max_ /cm^-1^: 2943, 2770, 1658, 1534, 1444, 763, 697. HRMS (ESI+) m/z found [M+H]^+^ 303.1234, C_17_H_20_ClN_2_O^+^ 303.1264.

***N*-((2-chloro-[1,1'-biphenyl]-4-yl)methyl)-3-(methylsulfonamido)propan-1-aminium chloride (18):** Prepared by general method **g** using aldehyde **10b** (26.0 mg, 0.120 mmol), 3-(methylsulfonamido)propan-1-aminium chloride^[[3]](#footnote-3)^ (24.9 mg, 0.132 mmol), methanol (0.430 mL), Et_3_N (33.4 μL, 0.240 mmol) and sodium triacetoxyborohydride (35.6 mg, 0.168 mmol). The crude residue was purified by column chromatography (eluting gradient CH_2_Cl_2_:MeOH 100:0 to 95:5, R*_f_* = 0.30, CH_2_Cl_2_:MeOH 90:10) to give the free amine (19.0 mg, 45%). The amine (19.0 mg, 0.0538 mmol) was then converted to the hydrochloride salt according general method **c** using hydrochloric acid (0.269 mL, 2 M in diethyl ether) to provide the title compound as a white solid (5.10 mg, 24%). M.p. = 218-220 °C. δ_H_/ppm (500 MHz, DMSO-d_6_): 9.04 (2H, br s), 7.78 (1H, d, *J* = 1.7 Hz), 7.57 (1H, dd, *J* = 7.9 and 1.7 Hz), 7.52 – 7.47 (3H, m), 7.46 – 7.41 (3H, m), 7.15 (1H, t, *J* = 6.0 Hz), 4.21 (2H, s), 3.04 (2H, app dd, *J* = 13.0 and 6.7 Hz), 3.00 (2H, br s), 2.91 (3H, s), 1.90 – 1.81 (2H, m). δ_C_/ppm (126 MHz, DMSO-d_6_): 140.3, 138.1, 133.3, 131.7, 131.3, 129.2, 129.2, 128.4, 128.1, 49.2, 44.6, 40.1 (under solvent peak), 39.9 (under solvent peak), 26.3. ν_max_/cm^-1^: 2901, 2764, 1443, 1304, 1142, 1082, 763, 698. HRMS (ESI+) m/z found [M+H]^+^ 353.1074, C_17_H_22_ClN_2_O_2_S required 353.1090.

**Methyl 3-((3-((*tert*-butoxycarbonyl)amino)propyl)amino)-3-oxopropanoate (20a):** To a solution of *N*-Boc-1,3-propanediamine (1.74 mL, 10.0 mmol) in CH_2_Cl_2_ (100 mL) was added methyl 3-chloro-3-oxopropionate (1.18 mL, 11.0 mmol), followed by saturated aqueous solution of sodium bicarbonate (excess). After vigorous stirring for 2 h, the layers were separated, the organic phase was washed with 2 M HCl, dried (MgSO_4_) and the solvent was removed under reduced pressure. The crude product was purified by flash column chromatography (eluting gradient CH_2_Cl_2_:MeOH, 98:2 to 97:3) to provide the desired compound as an off-white solid (2.44 g, 89%). R*_f_* = 0.16 (CH_2_Cl_2_:MeOH 98:2). M.p. = 49-50 °C. δ_H/_ppm (400 MHz, CDCl_3_): 7.27 (1H, br s (under solvent peak)), 4.95 (1H, br s), 3.73 (3H, s), 3.38 – 3.27 (4H, m), 3.14 (2H, t, *J* = 5.5 Hz), 1.71 – 1.59 (2H, m), 1.42 (9H, s). δ_C_/ppm (101 MHz, CDCl_3_): 169.7, 165.5, 156.6, 79.4, 52.6, 41.6, 37.3, 36.4, 30.1, 28.5. ν_max_/cm^-1^: 3309, 2973, 1755, 1669, 1630, 1533, 1274, 1159, 681. HRMS (ESI+) *m/z* found [M+H]^+^ 275.1591, C_12_H_23_N_2_O_5_^+^ required 275.1607.

**3-(3-Methoxy-3-oxopropanamido)propan-1-aminium 2,2,2-trifluoroacetate (20b):** Compound **20a** (485 mg, 1.77 mmol) was treated with a 1:1 mixture of CH_2_Cl_2_:TFA (3.54 mL) at 0 ˚C and stirred at rt until the starting material was consumed (30 min). The volatiles were evaporated under reduced pressure to give the product as a yellow amorphous solid (505 mg, 99%). δ_H/_ppm (400 MHz, DMSO-d_6_): 8.26 (1H, t, *J* = 5.5 Hz), 7.77 (3H, br s), 3.61 (3H, s), 3.24 (2H, s), 3.13 (2H, app q, *J* = 6.7 Hz), 2.89 – 2.71 (2H, m), 1.76 – 1.63 (2H, m). δ_C_/ppm (101 MHz, DMSO-d_6_): 168.4, 165.6, 158.4 (q, *J* = 35.6 Hz), 51.8, 42.3, 36.8, 35.8, 27.3. ν_max_/cm^-1^: 2957, 1654, 1175, 1128, 721. HRMS (ESI+) *m/z* found [M+H]^+^ 175.1090, C_7_H_15_N_2_O_3_^+^ required 175.1083.

***N*-((2-chloro-[1,1'-biphenyl]-4-yl)methyl)-3-(3-methoxy-3-oxopropanamido)propan-1-aminium chloride (19):** Prepared by general method **g** using aldehyde **10b** (65.0 mg, 0.300 mmol), ammonium salt **20b** (130 mg, 0.450 mmol), MeOH (1.07 mL), Et_3_N (83.4 μL, 0.600 mmol) and sodium triacetoxyborohydride (89.0 mg, 0.420 mmol). The crude residue was purified by column chromatography (eluting gradient CH_2_Cl_2_:MeOH 98:2 to 95:5, R*_f_* = 0.14, CH_2_Cl_2_:MeOH 9:1) to provide the free amine (52.7 mg, 47%). The amine (11.0 mg, 0.0293 mmol) was then converted to the hydrochloric salt according general method **c** using hydrochloric acid (0.147 mL, 2 M in diethyl ether) to provide the title compound as a white solid (12.3 mg, 99%). M.p. = 176-179 °C. δ_H_/ppm (500 MHz, DMSO-d_6_): 9.11 (2H, br s), 8.32 (1H, t, *J* = 5.7 Hz), 7.79 (1H, d, *J* = 1.7 Hz), 7.57 (1H, dd, *J* = 7.9 and 1.7 Hz), 7.52 – 7.46 (3H, m), 7.46 – 7.39 (3H, m), 4.24 – 4.14 (2H, m), 3.61 (3H, s), 3.26 (2H, s), 3.21 – 3.11 (2H, m), 2.96 (2H, dt, *J* = 12.3 and 7.6 Hz), 1.86 – 1.76 (2H, m). δ_C_/ppm (126 MHz, DMSO-d_6_): 168.4, 165.6, 140.3, 138.1, 133.2, 131.7, 131.3 (x2), 129.2, 129.2, 128.4, 128.0, 51.9, 49.1, 44.6, 42.3, 35.9, 25.8. ν_max_/cm^-1^: 3294, 2945, 2753, 1750, 1636, 1545, 1439, 1259, 1154, 1021, 702. HRMS (ESI+) *m/z* found [M+H]^+^ 375.1479, C_20_H_24_N_2_O_3_Cl^+^ required 375.1475.

**3-((3-(((2-Chloro-[1,1'-biphenyl]-4-yl)methyl)ammonio)propyl)amino)-3-oxopropanoate (20):** Prepared by general method **h** using methyl ester **19** (36.0 mg, 0.0960 mmol), 2.40 mL of 0.3 M LiOH solution, THF (4.80 mL) and 72.0 μL of 4N HCl solution in dioxane. The compound was obtained as the lithium chloride salt in quantitative yield. M.p. = 122-124 °C. δ_H_/ppm (400 MHz, DMSO-d_6_): 8.33 (1H, t, *J* = 5.8 Hz), 7.83 (1H, d, *J* = 1.5 Hz), 7.61 (1H, dd, *J* = 7.9 and 1.6 Hz), 7.52 – 7.46 (3H, m), 7.45 – 7.37 (2H, m), 3.20 – 3.12 (4H, m), 2.94 (2H, t, *J* = 7.4 Hz), 1.91 – 1.78 (2H, m). δ_C_/ppm (126 MHz, MeOD-d_4_): 171.5, 170.4, 143.1, 139.9, 134.0, 133.5, 133.2, 132.5, 130.3, 129.9, 129.3, 129.1, 51.6, 46.1, 36.9, 30.8, 27.4. ν_max_/cm^-1^: 3402, 2902, 2766, 1622, 1444, 763, 697. HRMS (ESI+) *m/z* found [M+H]^+^ 361.1292, C_19_H_22_ClN_2_O_3_^+^ required 361.1319.

**3-((3-((*Tert*-butoxycarbonyl)amino)propyl)amino)-3-oxopropanoic acid (21a):** Prepared by general method **h** using methyl ester **20a** (1.51g, 5.50 mmol), 55 mL of 0.3 M LiOH solution, THF (110 mL) and 4.10 mL of 4N HCl solution in dioxane. The crude product was purified by column chromatography (eluting gradient CH_2_Cl_2_:MeOH 90:10 to 80:20) to provide the desired compound as a white solid (1.39 g, 97%). R*_f_* = 0.23 (CH_2_Cl_2_:MeOH 85:15). M.p. = 157-163 °C. δ_H_/ppm (400 MHz, DMSO-d_6_): 8.59 (1H, br s), 6.81 (1H, t, *J* = 5.5 Hz), 3.06-2.97 (4H, m), 2.92-2.86 (2H, m), 1.48 (2H, app quint, *J* = 6.8 Hz), 1.35 (9H, s). δ_C_/ppm (101 MHz, MeOD-d_4_): 179.5, 178.2, 165.09, 87.0, 58.0, 53.6, 47.1, 45.7, 39.0, 37.8. ν_max_/cm^-1^: 3333, 2978, 1637, 1394, 1020. HRMS (ESI+) *m/z* found [M+Na]^+^ 283.1278, C_11_H_20_N_2_O_5_Na^+^ required 283.1270.

**3-(3-((3-(Methoxycarbonyl)phenyl)amino)-3-oxopropanamido)propan-1-aminium chloride (21b):** To a solution of compound **21a** (937 mg, 3.60 mmol) in DMF (18.0 mL) was added *m*-amino methylbenzoate (653 mg, 4.32 mmol), followed by EDC·HCl (690 mg, 3.60 mmol) and NMM (396 µL, 3.60 mmol). The reaction mixture was stirred at rt overnight and then diluted with CH_2_Cl_2_ and washed with 2 M aqueous solution of sodium carbonate and brine (x 5). The crude product was purified by column chromatography (eluting gradient CH_2_Cl_2_:MeOH 98:2 to 97:3, R*_f_* = 0.37, CH_2_Cl_2_:MeOH 92:8) to provide the desired amine (822 mg, 58% yield). This material was treated with 517 μL of 4N HCl in dioxane. After 2 h the volatiles were evaporated to afford the product as a white solid (630 mg, 92% yield). M.p. = 201-204 °C. δ_H_/ppm (400 MHz, DMSO-d_6_): 10.58 (1H, s), 8.39 (1H, t, *J* = 5.6 Hz), 8.31 (1H, t, *J*= 1.5 Hz), 7.96 (br 3H, s), 7.85 – 7.80 (1H, m), 7.66 – 7.61 (1H, m), 7.45 (1H, t, *J* = 7.9 Hz), 3.85 (1H, s), 3.31 (2H, s), 3.21 – 3.11 (2H, m), 2.88 – 2.74 (2H, m), 1.78 – 1.69 (2H, m). δ_C_/ppm (101 MHz, DMSO-d_6_): 166.8, 166.1, 166.1, 139.4, 130.1, 129.2, 124.0, 123.6, 119.6, 52.2, 44.8, 36.7, 35.8, 27.3. ν_max_/cm^-1^: 2946, 2768, 1714, 1444, 1261, 1059, 698. HRMS (ESI+) *m/z* found [M+H]^+^ 294.1436, C_14_H_20_N_3_O_4_^+^ required 294.1454.

**Methyl 3-(3-((3-(((2-chloro-[1,1'-biphenyl]-4-yl)methyl)amino)propyl)amino)-3-oxopropanamido) benzoate (21c):** Prepared by general method **g** using aldehyde **10b** (134 mg, 0.618 mmol), ammonium salt **21b** (306 mg, 0.927 mmol), MeOH (2.20 mL), Et_3_N (172 μL, 1.24 mmol) and sodium triacetoxyborohydride (183 mg, 0.865 mmol). The crude residue was purified by column chromatography (eluting gradient CH_2_Cl_2_:MeOH 95:5 to 90:10) to give the free amine as an amorphous white solid (163 mg, 53% yield). R*_f_* = 0.14 (CH_2_Cl_2_:MeOH 9:1). δ_H_/ppm (400 MHz, CDCl_3_): 9.92 (1H, s), 8.15 (1H, s), 7.88 (1H, dd, *J* = 8.1 and 1.3 Hz), 7.77 (1H, d, *J* = 7.8 Hz), 7.71 (1H, t, *J* = 4.8 Hz), 7.45 – 7.34 (7H, m), 7.31 (1H, d, *J* = 7.8 Hz), 7.28 – 7.23 (2H, m), 3.89 (3H, s), 3.81 (2H, s), 3.49 – 3.40 (2H, m), 3.35 (2H, s), 2.80 (2H, t, *J* = 6.1 Hz), 1.80 – 1.67 (2H, m). δ_C_/ppm (101 MHz, CDCl_3_): 167.9, 166.8, 165.1, 140.6, 139.5, 139.2, 138.1, 132.7, 131.7, 131.0, 129.6, 129.6, 129.2, 128.2, 127.8, 126.7, 125.6, 124.6, 121.1, 53.2, 52.3, 47.8, 43.8, 39.5, 28.4. ν_max_/cm^-1^: 3286, 1644, 1544, 1288, 754. HRMS (ESI+) *m/z* found [M+H]^+^ 494.1830, C_27_H_29_N_3_O_4_Cl required 494.1847.

**3-(3-((3-Carboxyphenyl)amino)-3-oxopropanamido)-*N*-((2-chloro-[1,1'-biphenyl]-4-yl)methyl)propan-1-aminium 2,2,2-trifluoroacetate (21):** Prepared by general method **h** using ester **21c** (59.3 mg, 0.120 mmol), 1.20 mL of 0.3 M LiOH solution, THF (2.40 mL) and 90.0 μL of 4N HCl solution in dioxane. The crude product was purified by HPLC *t_r_* 5.28 min (method: 40-70% B, 20 min) (41.4 mg, 72% yield). M.p. = 225-227 °C. δ_H_/ppm (500 MHz, DMSO-d_6_): 12.98 (1H, br s), 10.35 (1H, s), 8.87 (2H, br s), 8.31 (1H, t, *J* = 5.9 Hz), 8.26 (1H, t, *J* = 1.8 Hz), 7.79 (1H, ddd, *J* = 8.1, 2.2 and 1.1 Hz), 7.74 (1H, d, *J* = 1.6 Hz), 7.65 – 7.60 (1H, m), 7.53 (1H, dd, *J* = 7.9 and 1.7 Hz), 7.51 – 7.46 (3H, m), 7.46 – 7.40 (4H, m), 4.21 (2H, br s), 3.30 (2H, s), 3.24 – 3.18 (2H, m), 3.02 (2H, br s), 1.87 – 1.76 (2H, m). δ_C_/ppm (126 MHz, DMSO-d_6_): 167.1, 167.0, 166.1, 157.9 (q, *J* = 31.1 Hz), 140.3, 139.1, 138.0, 133.2, 131.7, 131.3, 131.2, 129.1, 129.1, 128.3, 128.0, 124.2, 123.2, 119.8, 49.2, 44.7 (x2), 35.8, 26.0. ν_max_/cm^-1^: 3295, 2795, 1668, 1641, 1538, 1188, 1134, 755, 698. HRMS (ESI+) *m/z* found [M+H]^+^ 480.1697, C_26_H_27_N_3_O_4_Cl^+^ required 480.1690.

Experimental procedures and characterizations of **CAM4066** and **pre-CAM4066** have been published elsewhere.^[[4]](#footnote-4)^

# **SI_4 Selected NMR spectra**


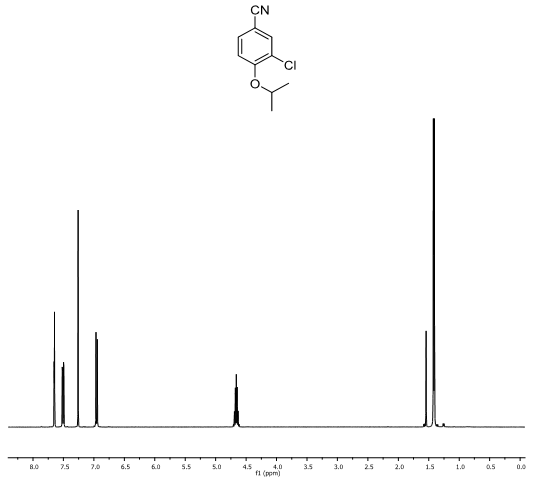

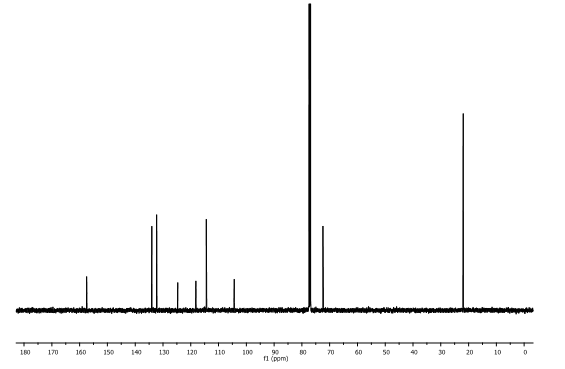


**3a**


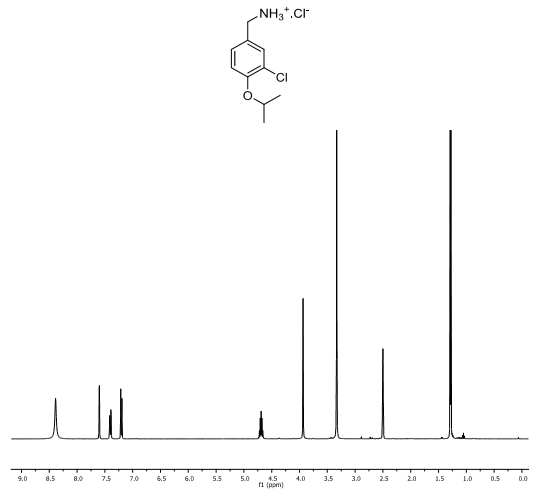

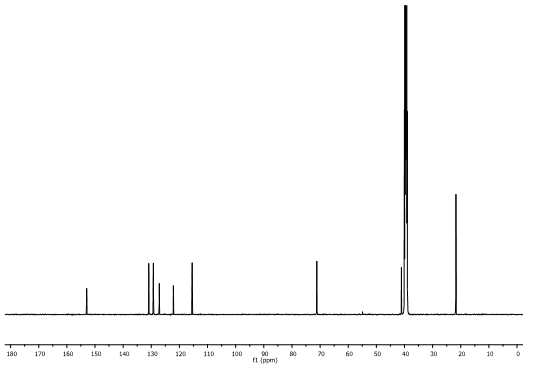


**3**


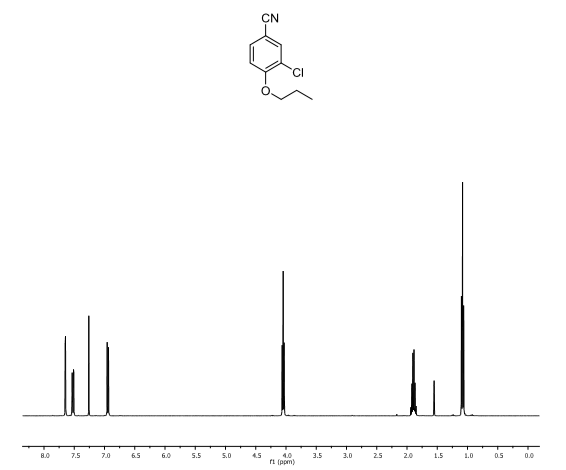


**4a**


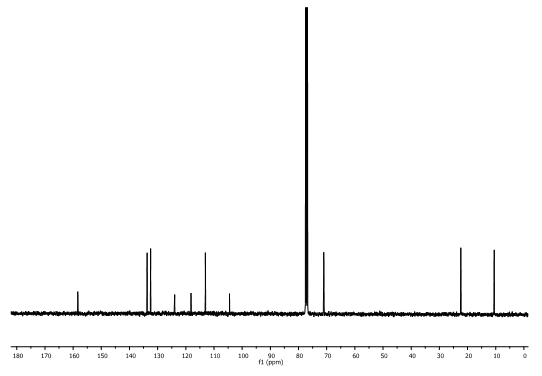


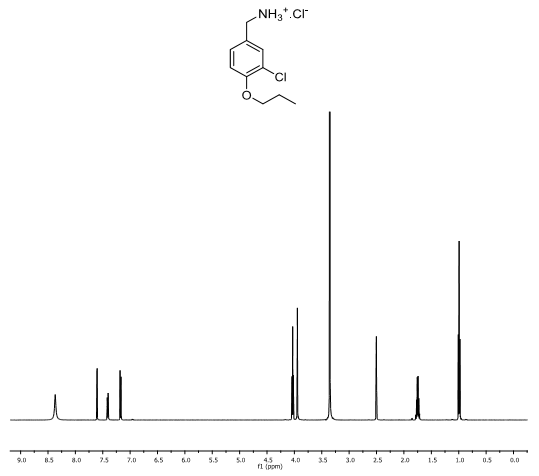


**4**


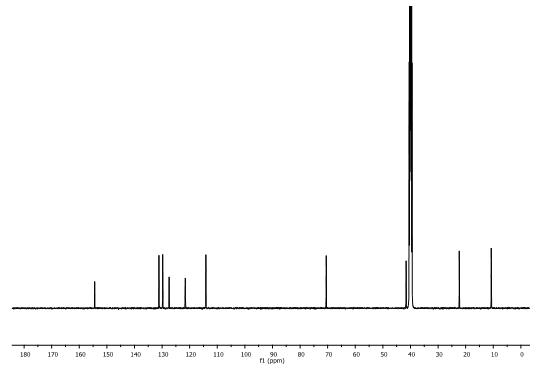


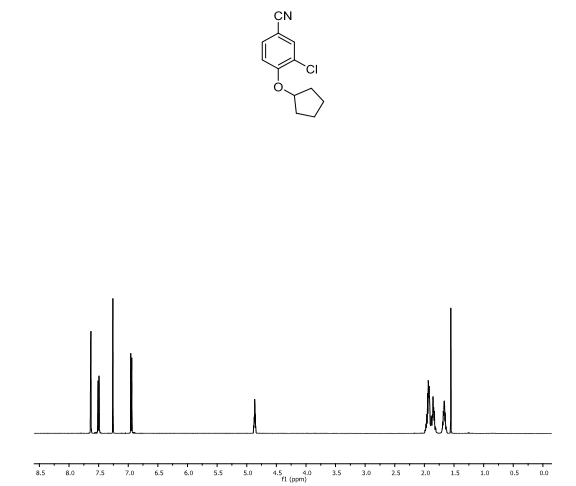


**5a**


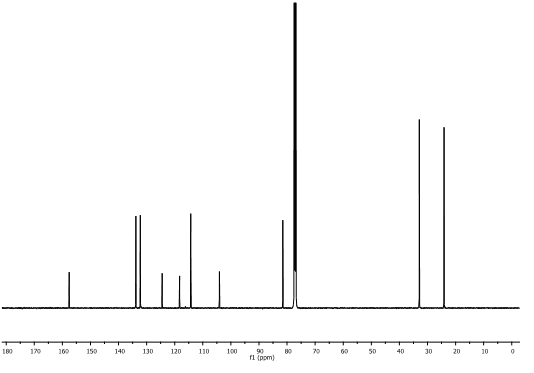


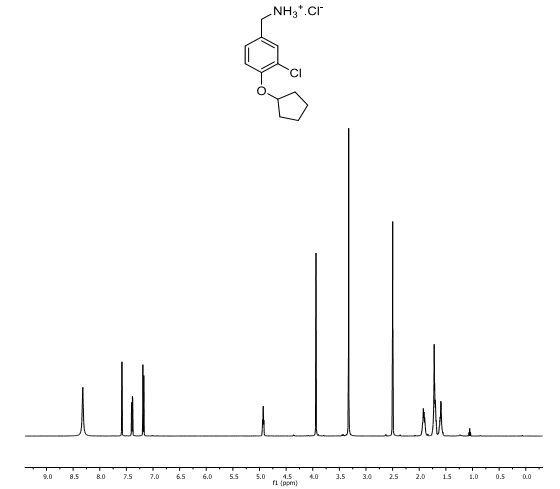

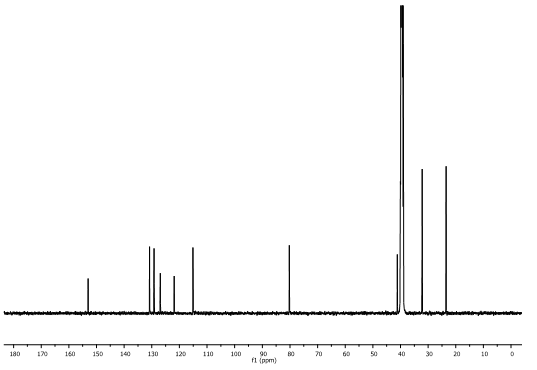


**5**


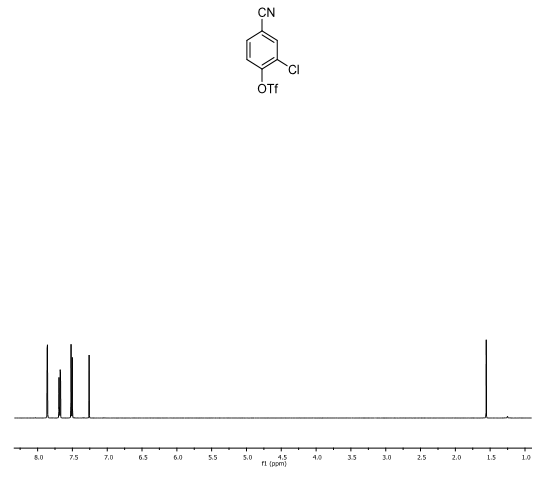

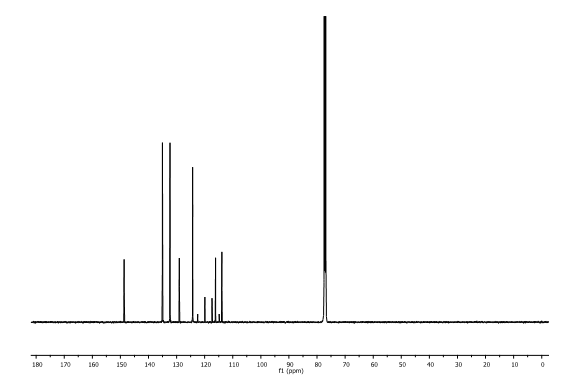


**6a**


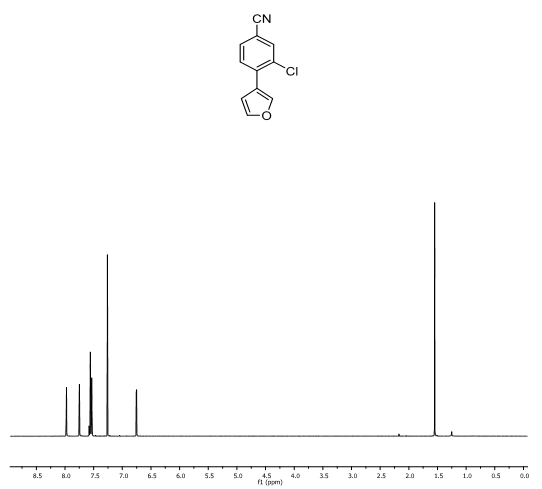

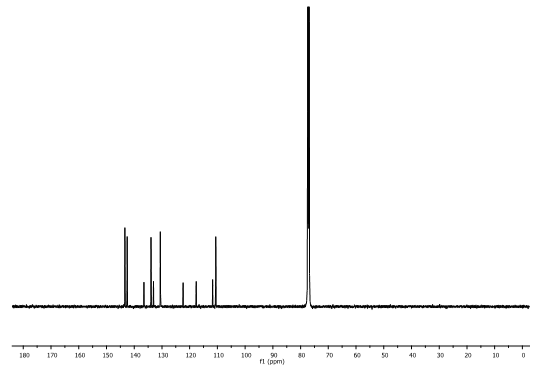

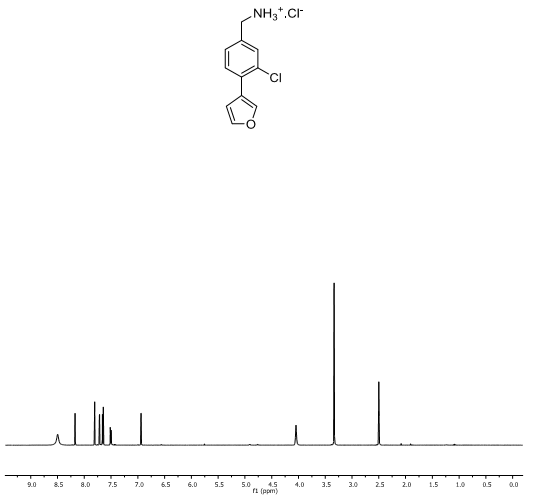

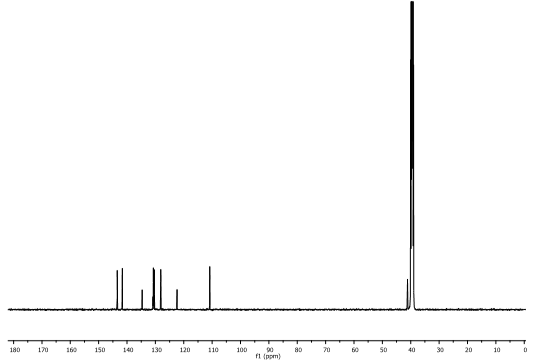


**6b**

**6**


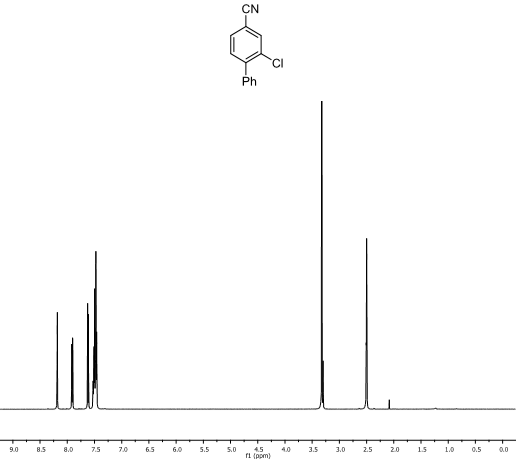

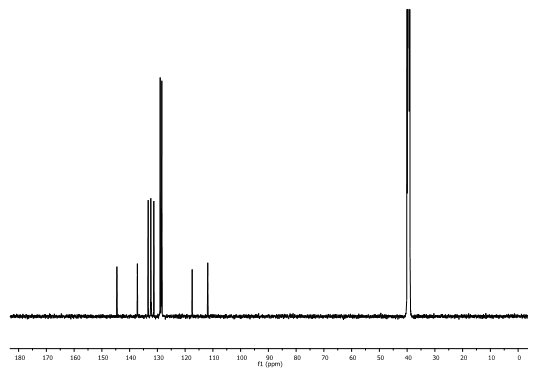

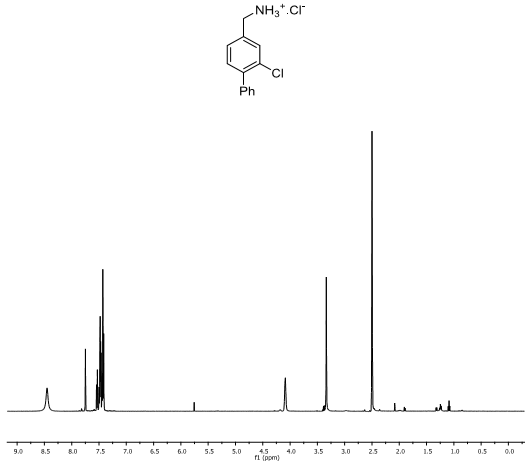

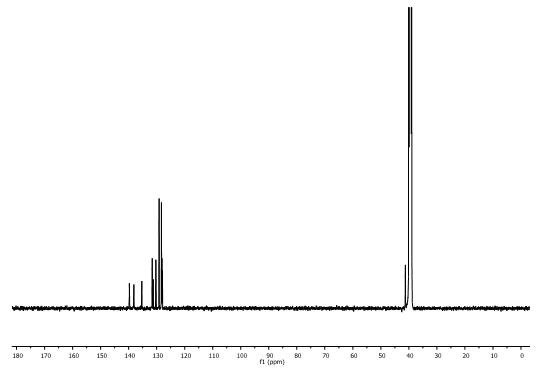


**7a**

**7**


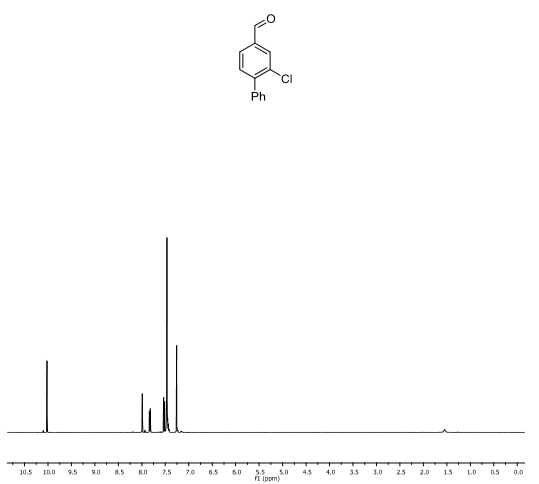

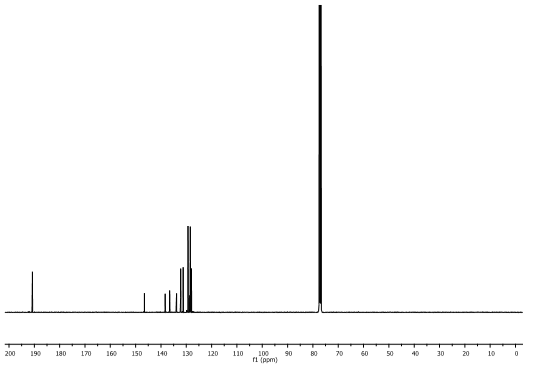


**10b**


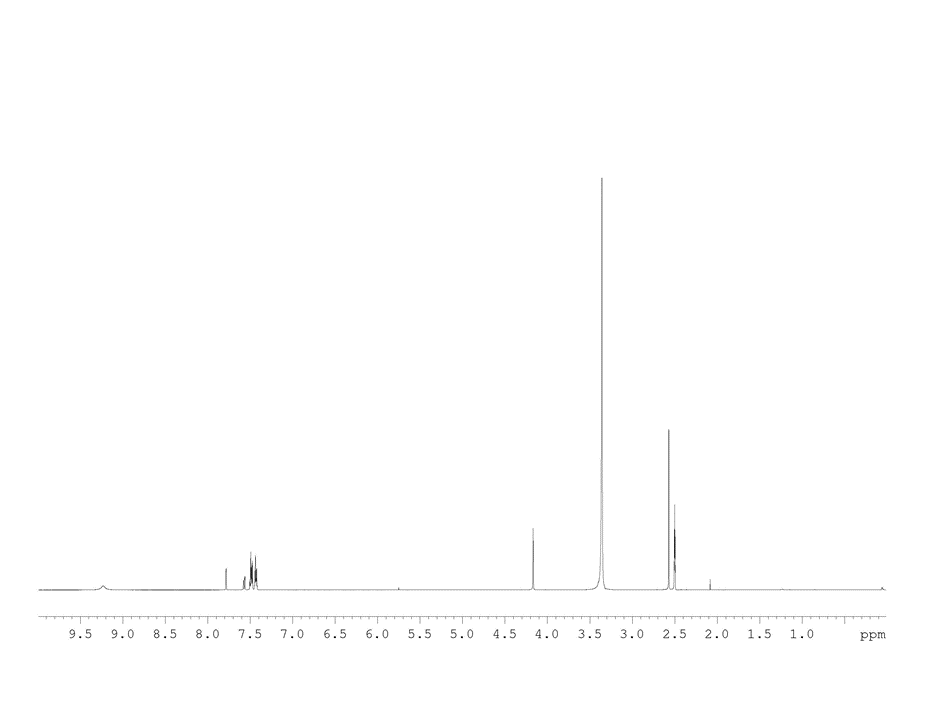

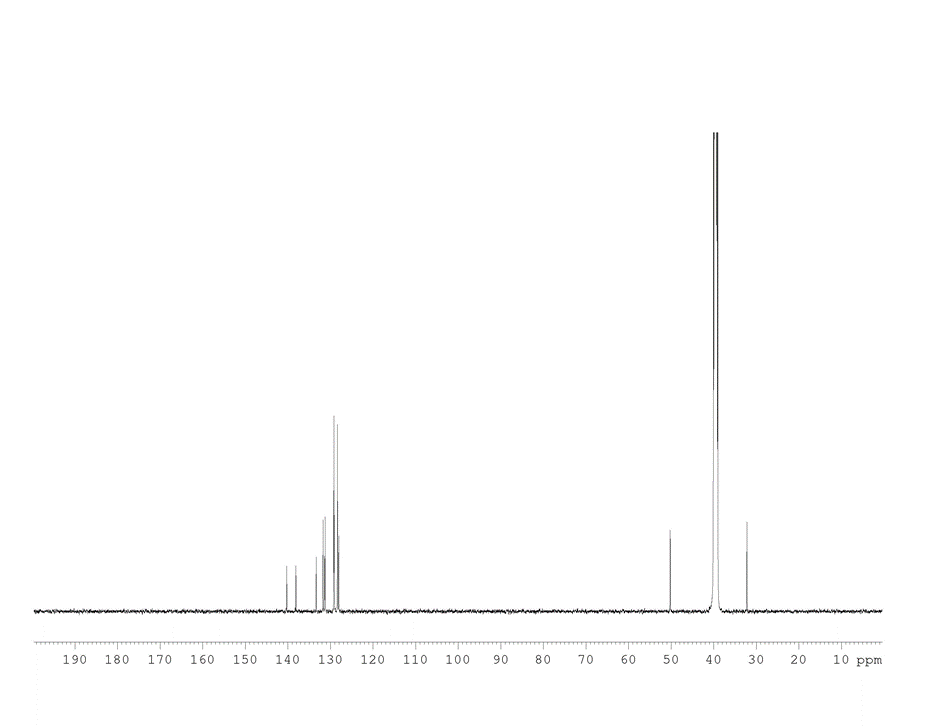


**10**


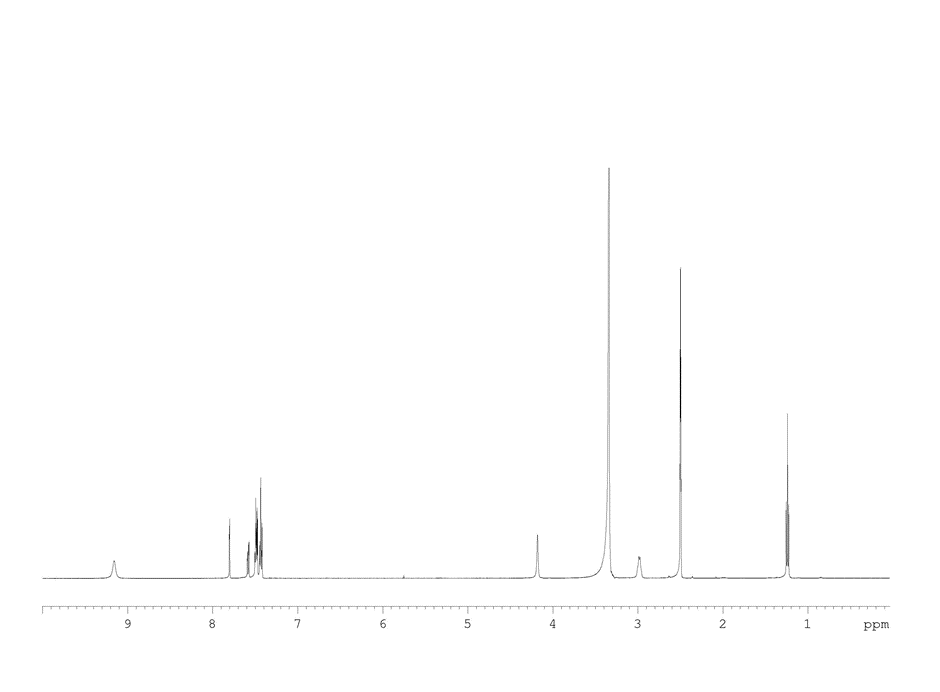

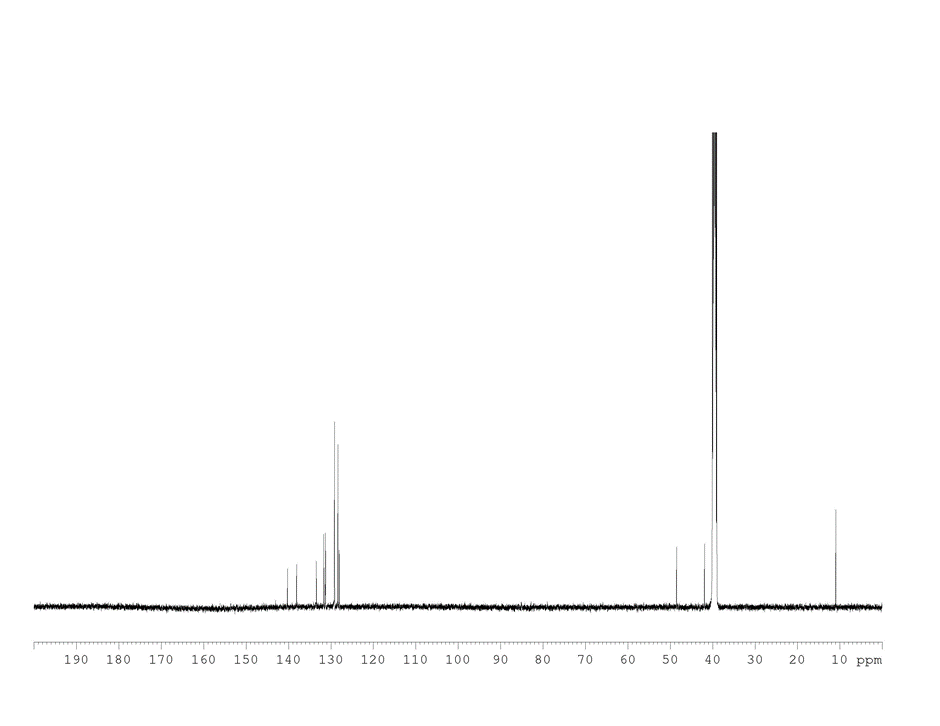


**11**


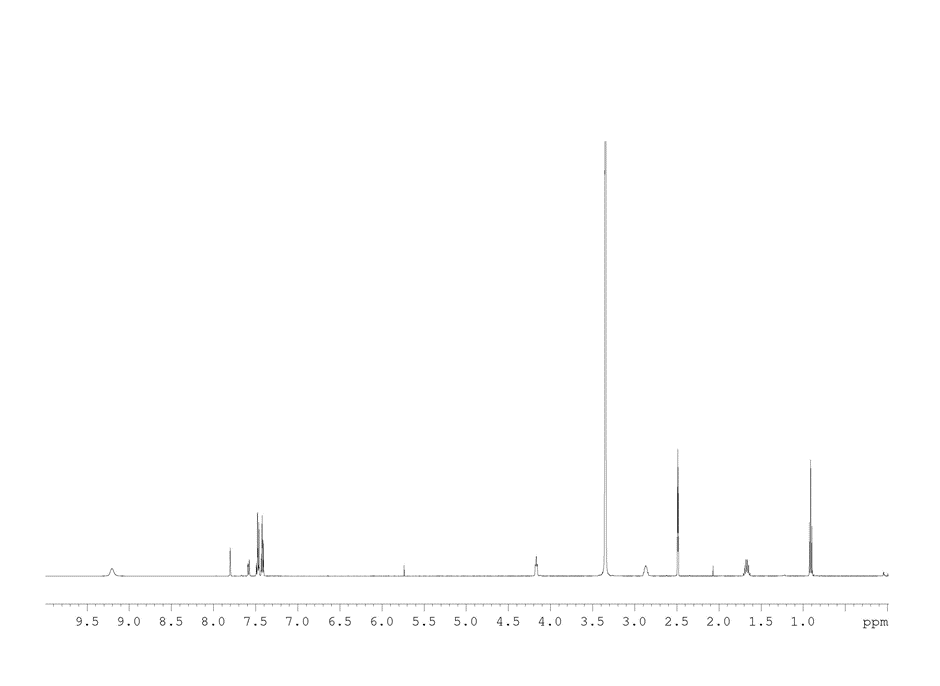

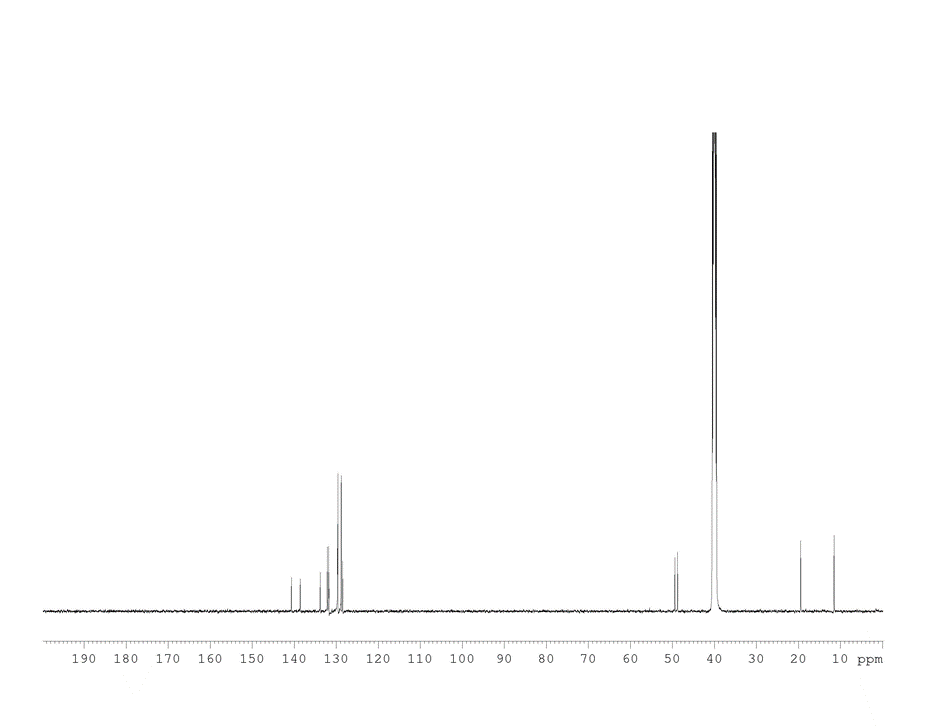


**12**


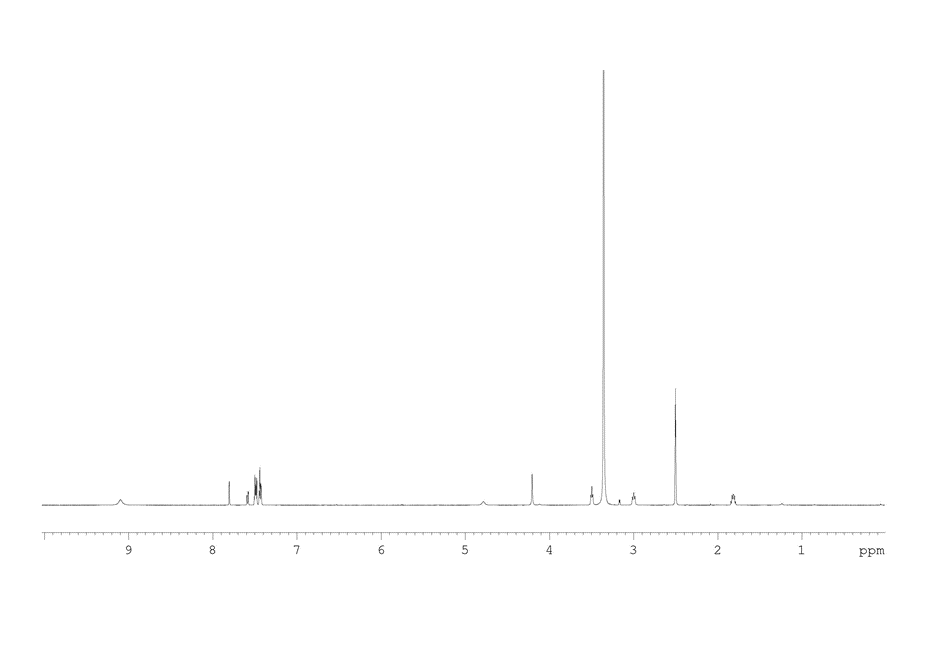


**13**


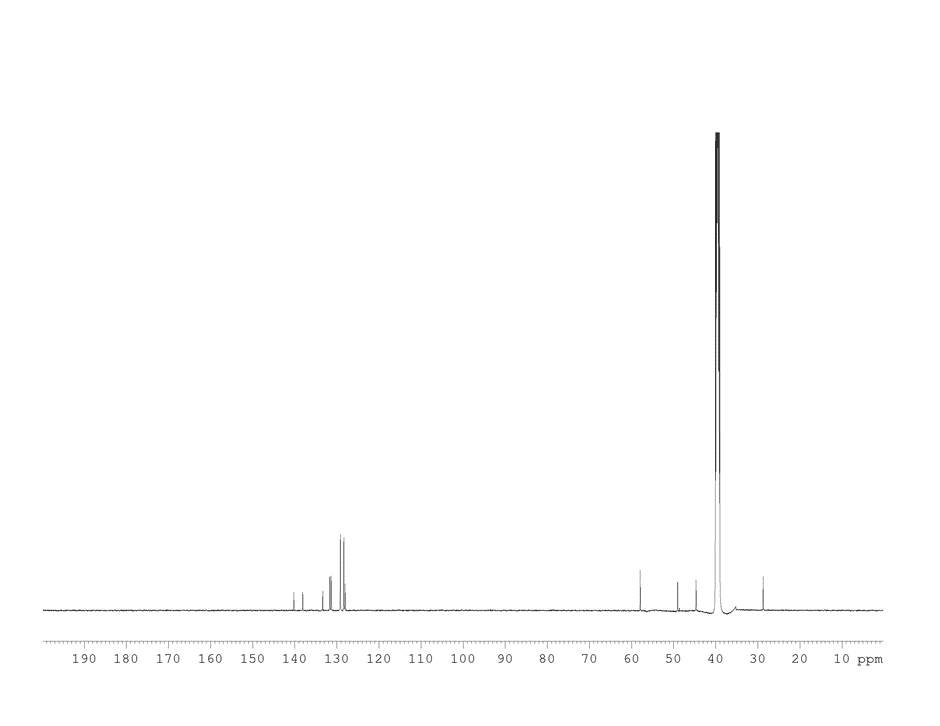


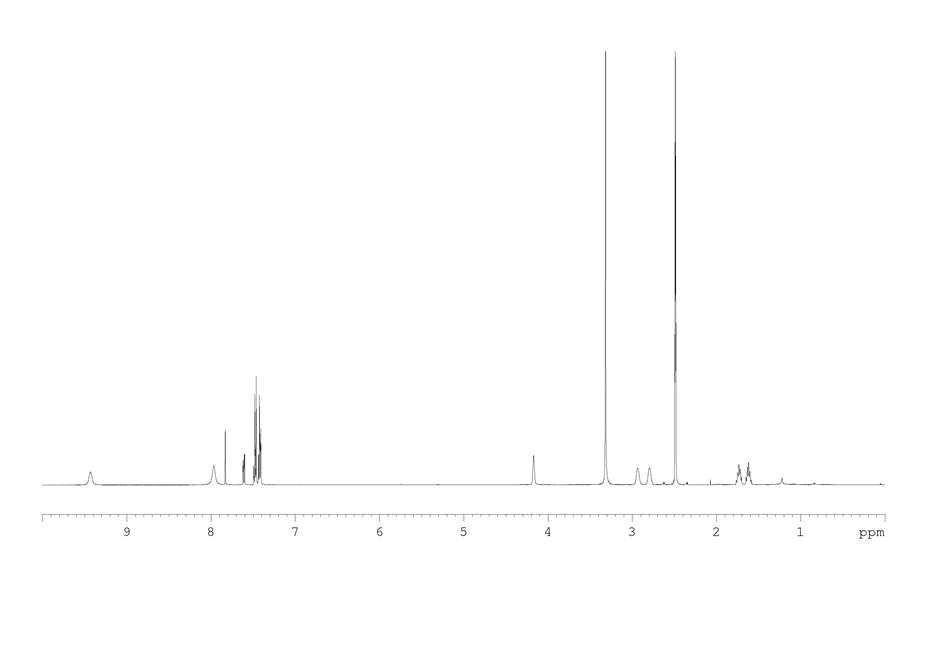

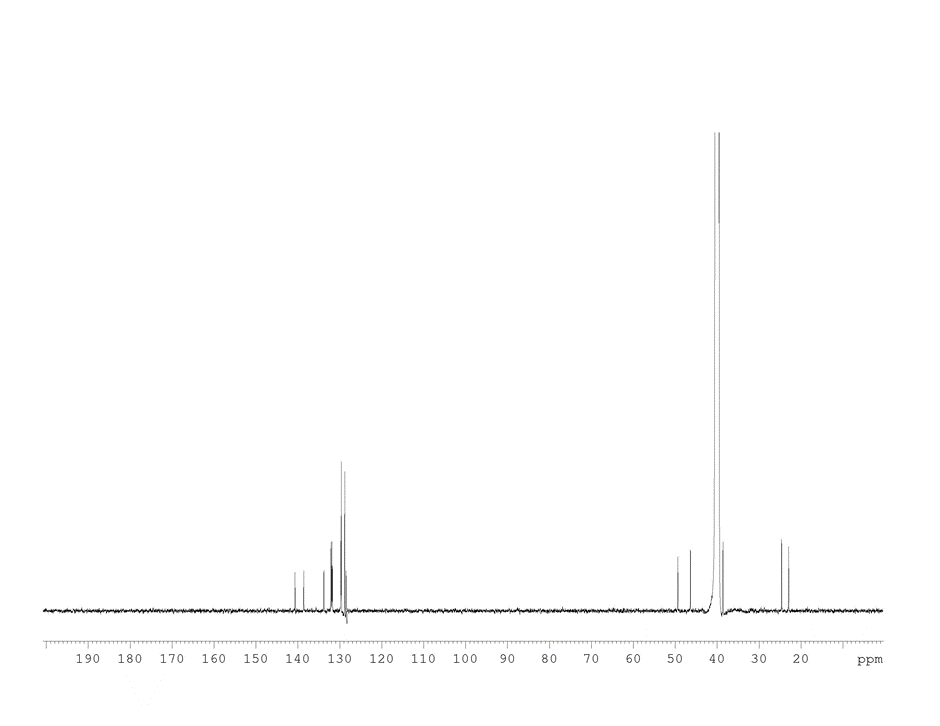

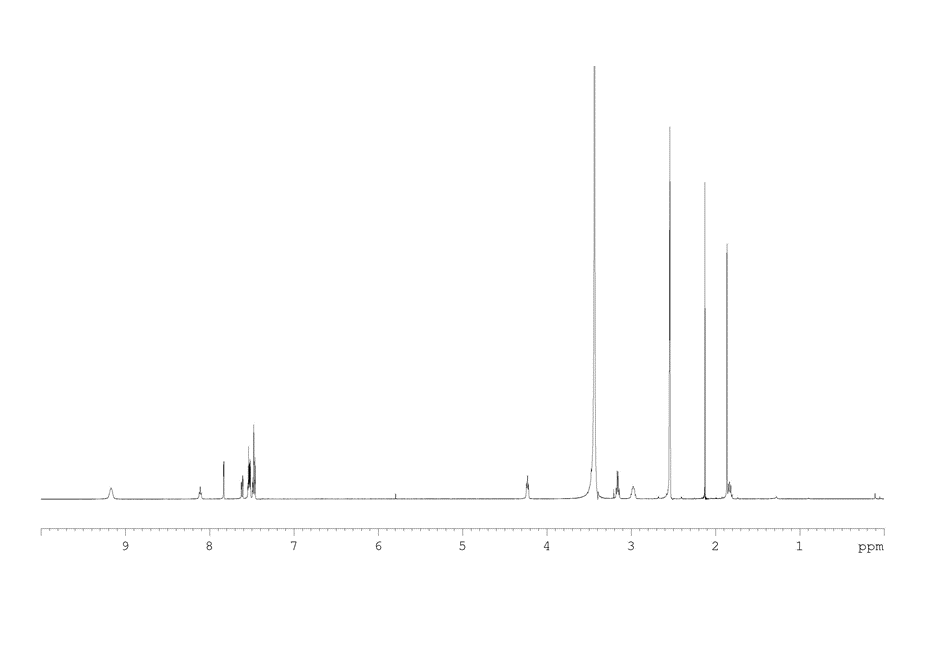

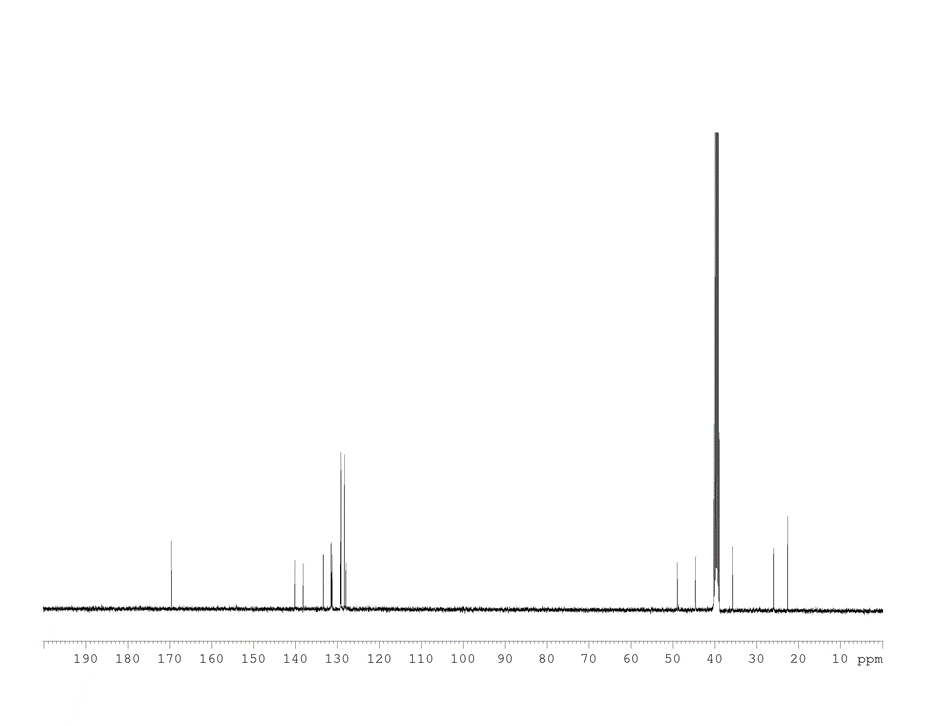

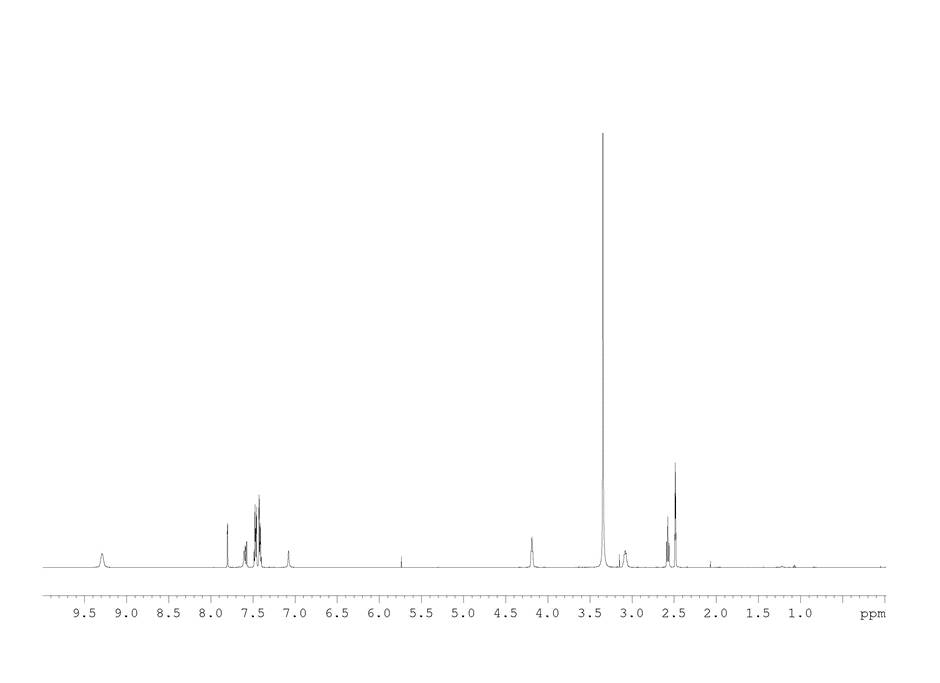

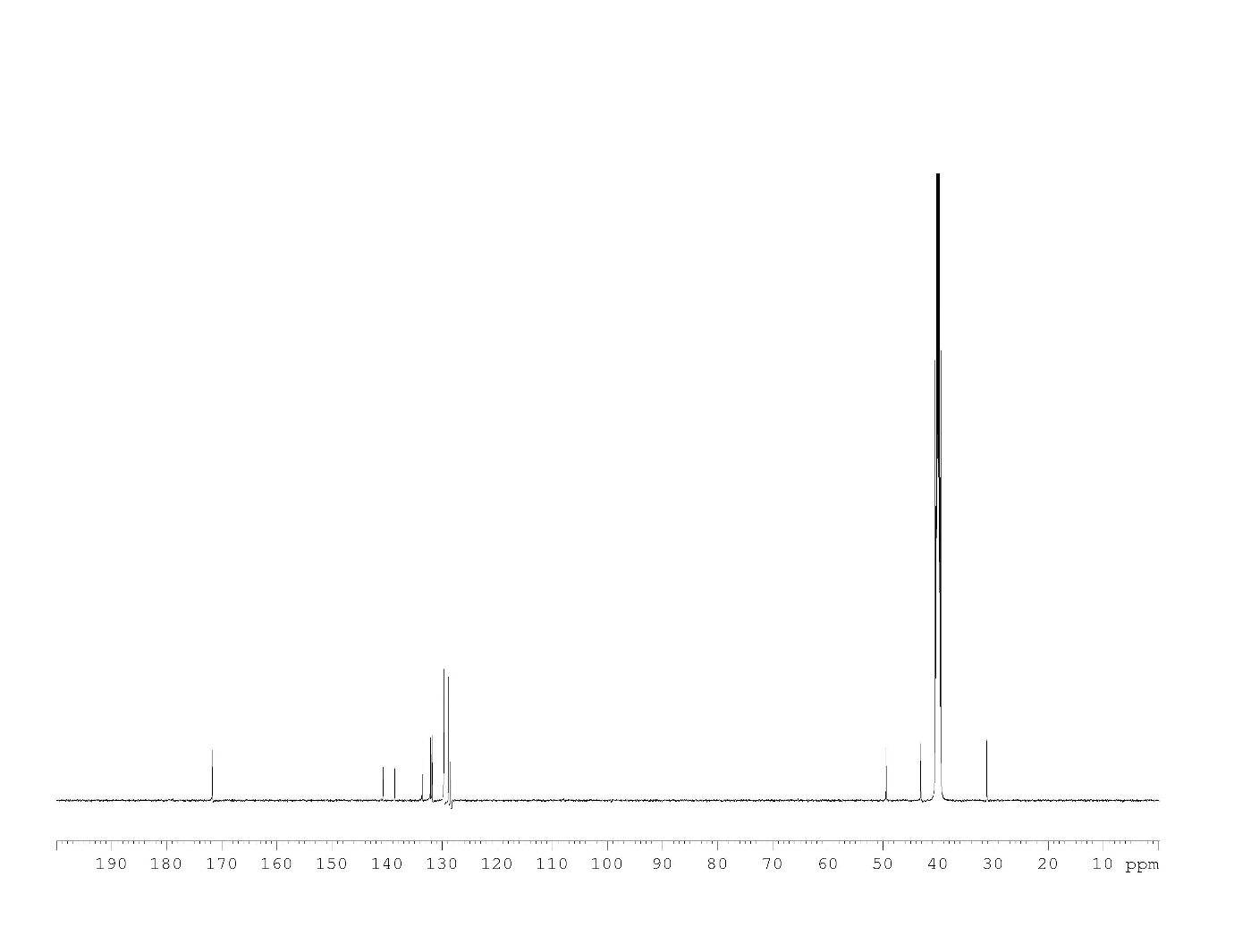


**14**

**15**

**16**


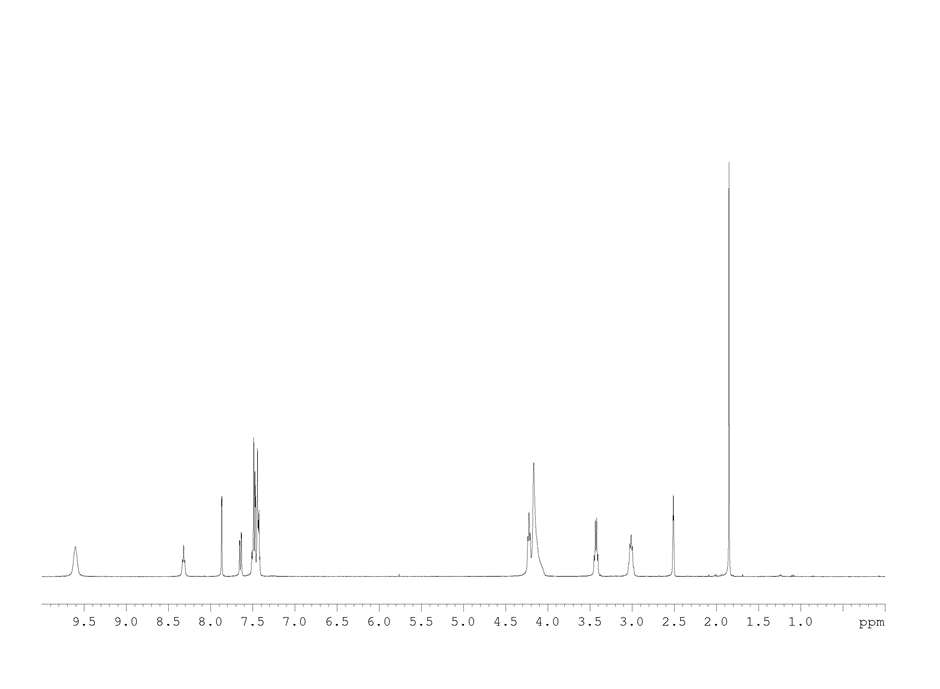

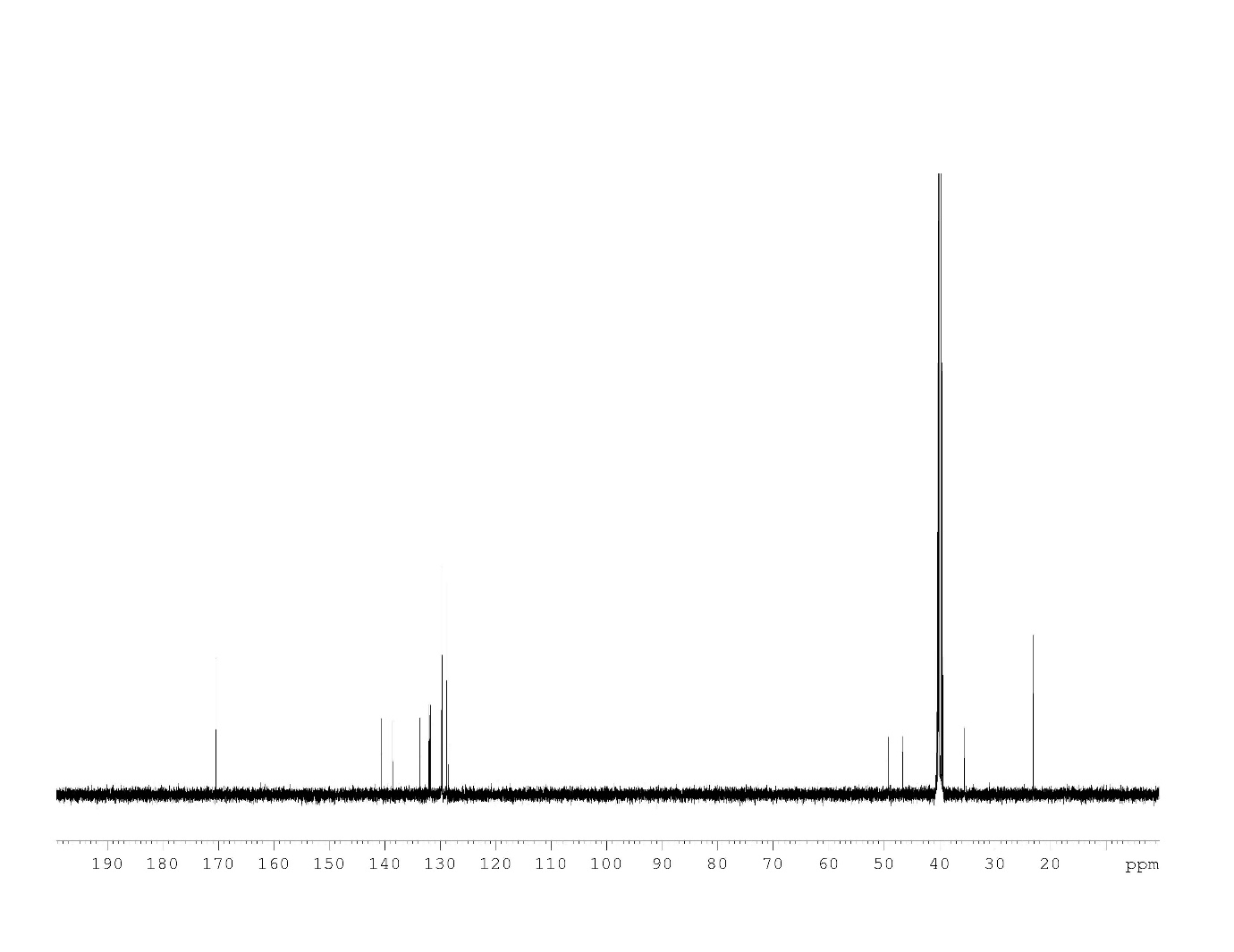


**17**


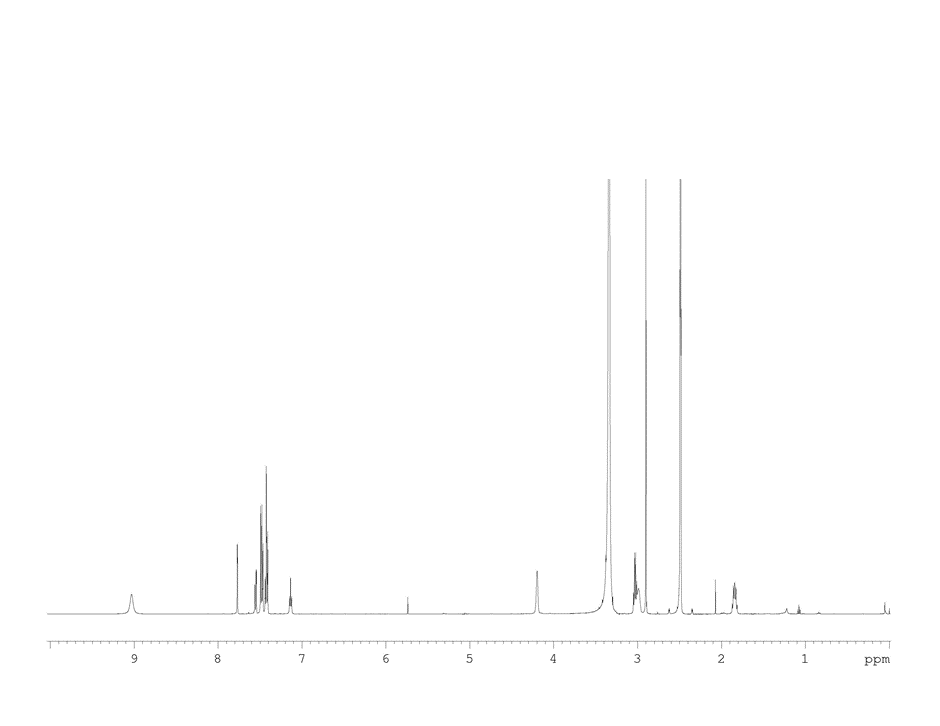

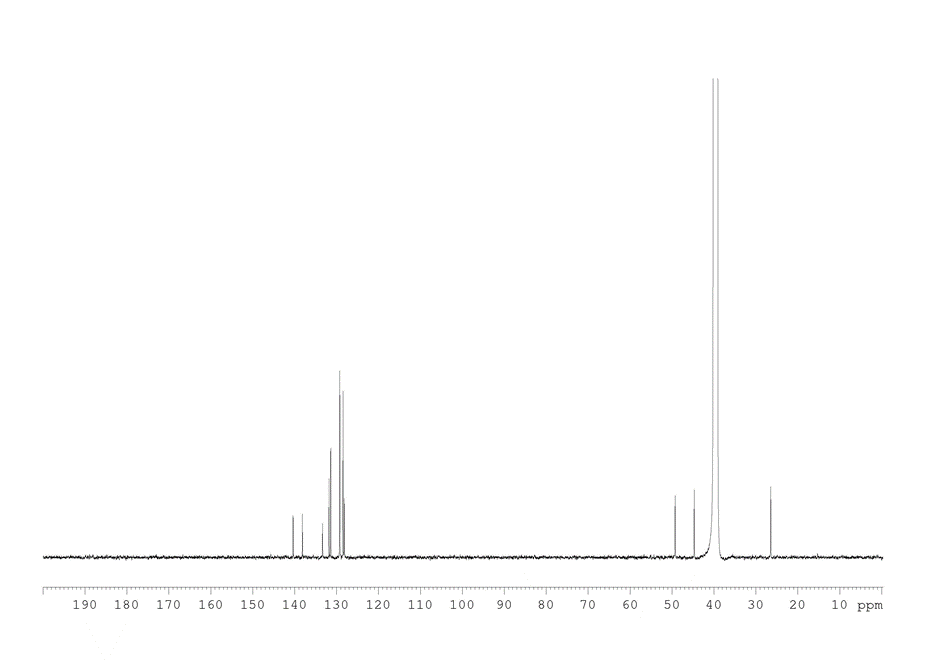


**18**


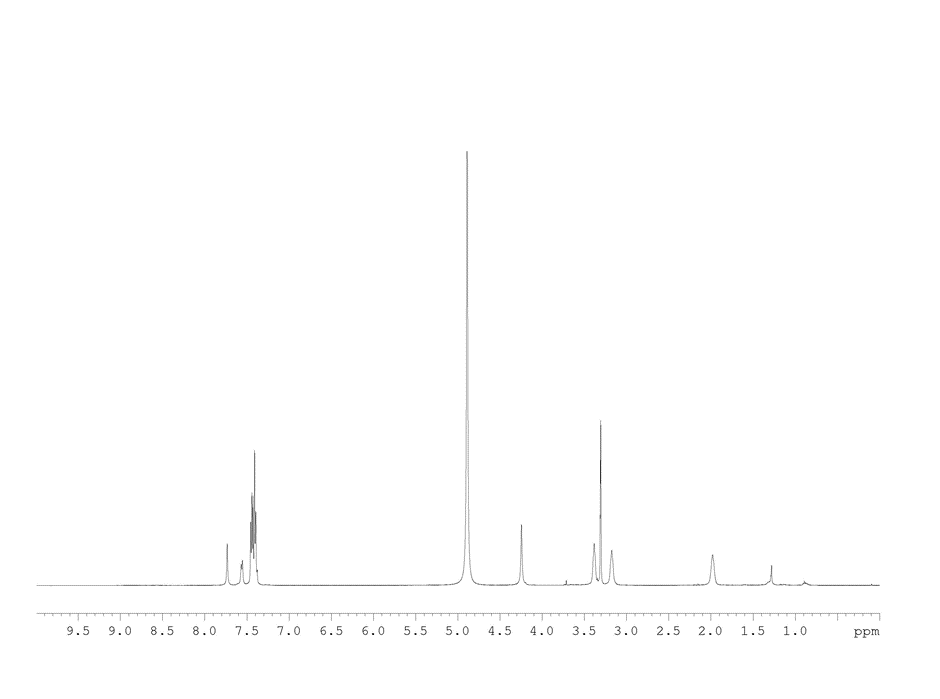

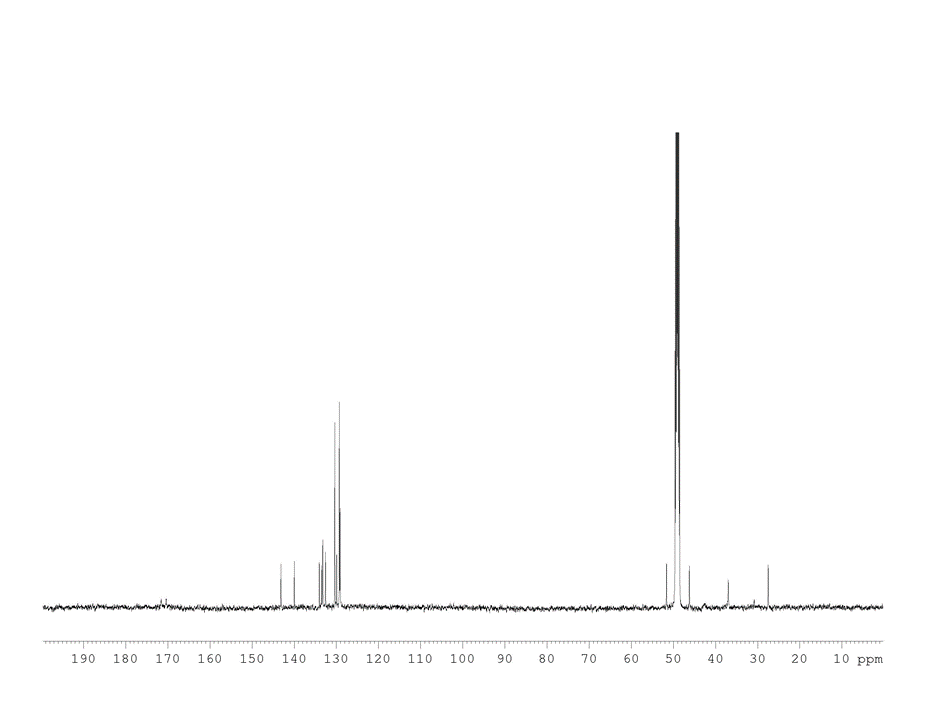


**20**


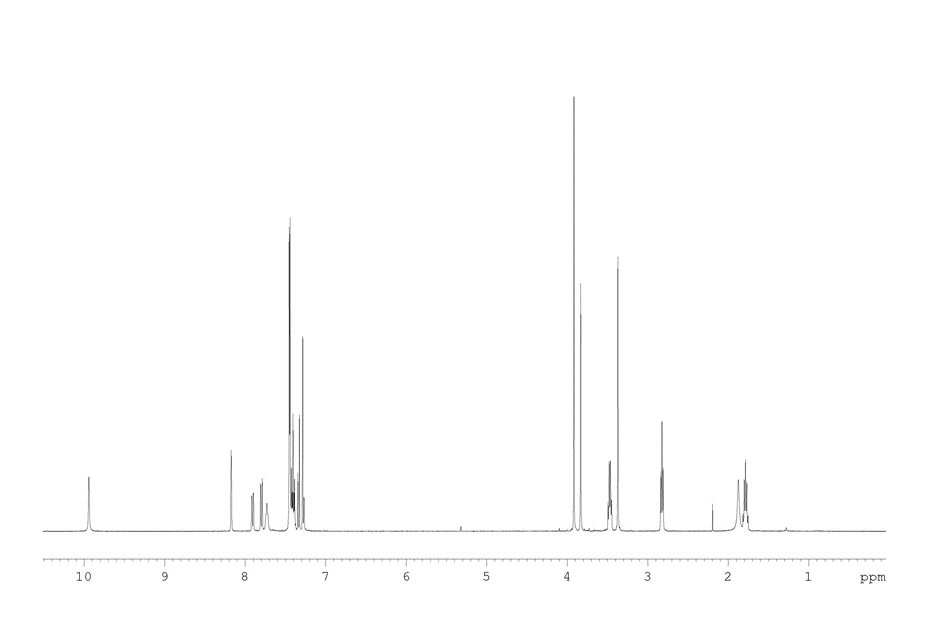

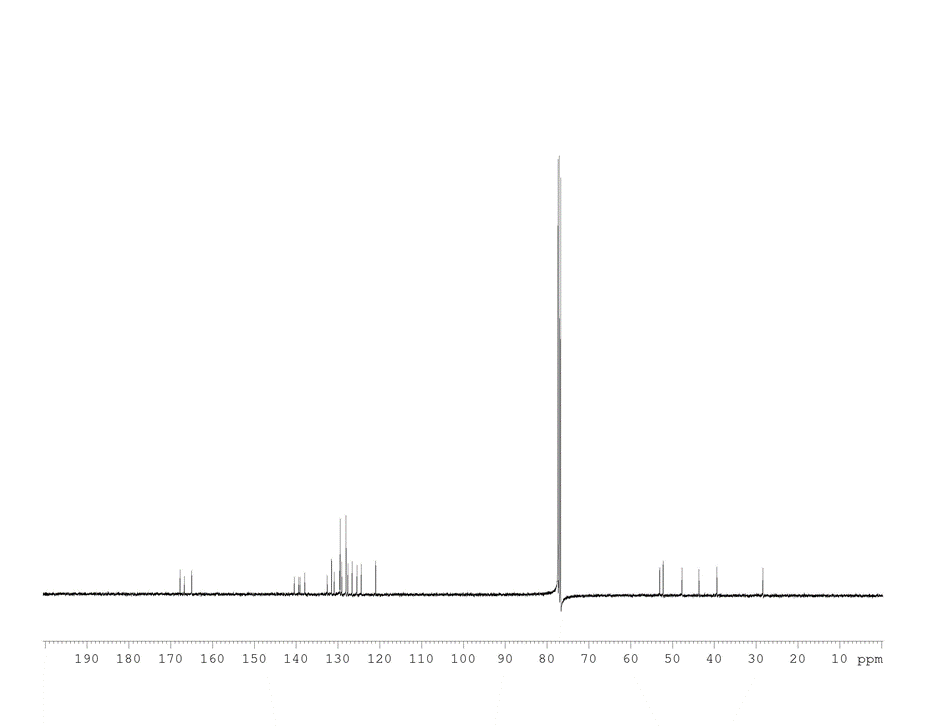


**21c**

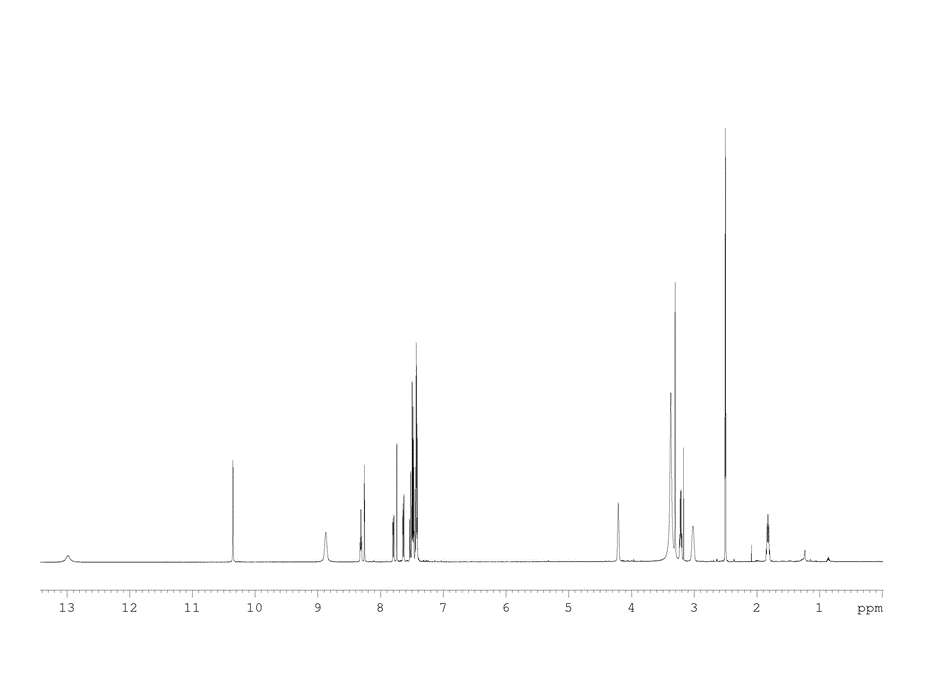

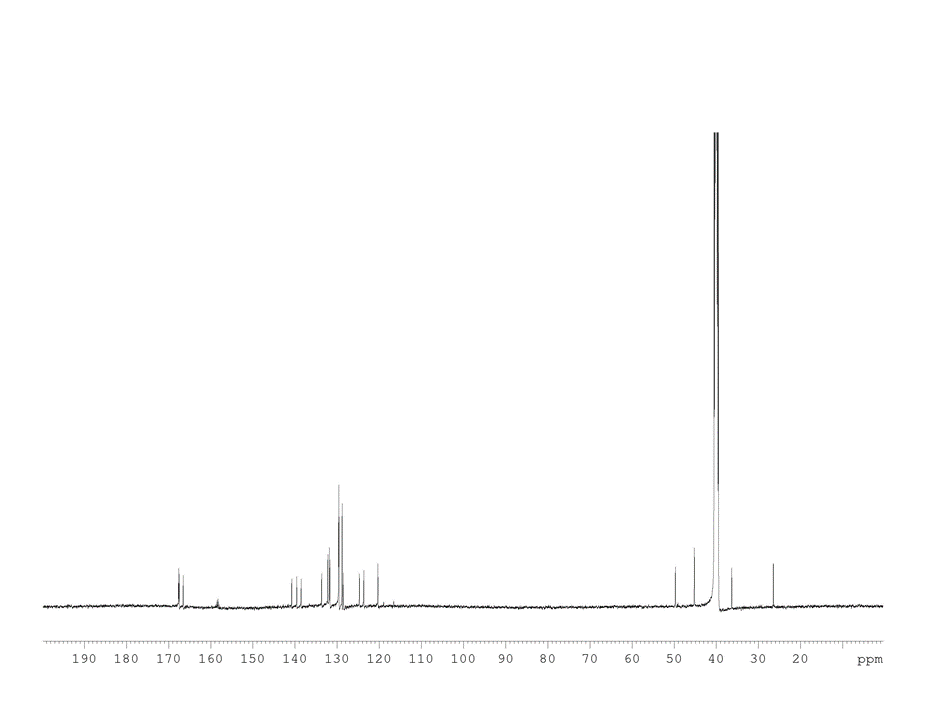


**21**

# **SI_5 Thermal shift results for 25 fragments**

**
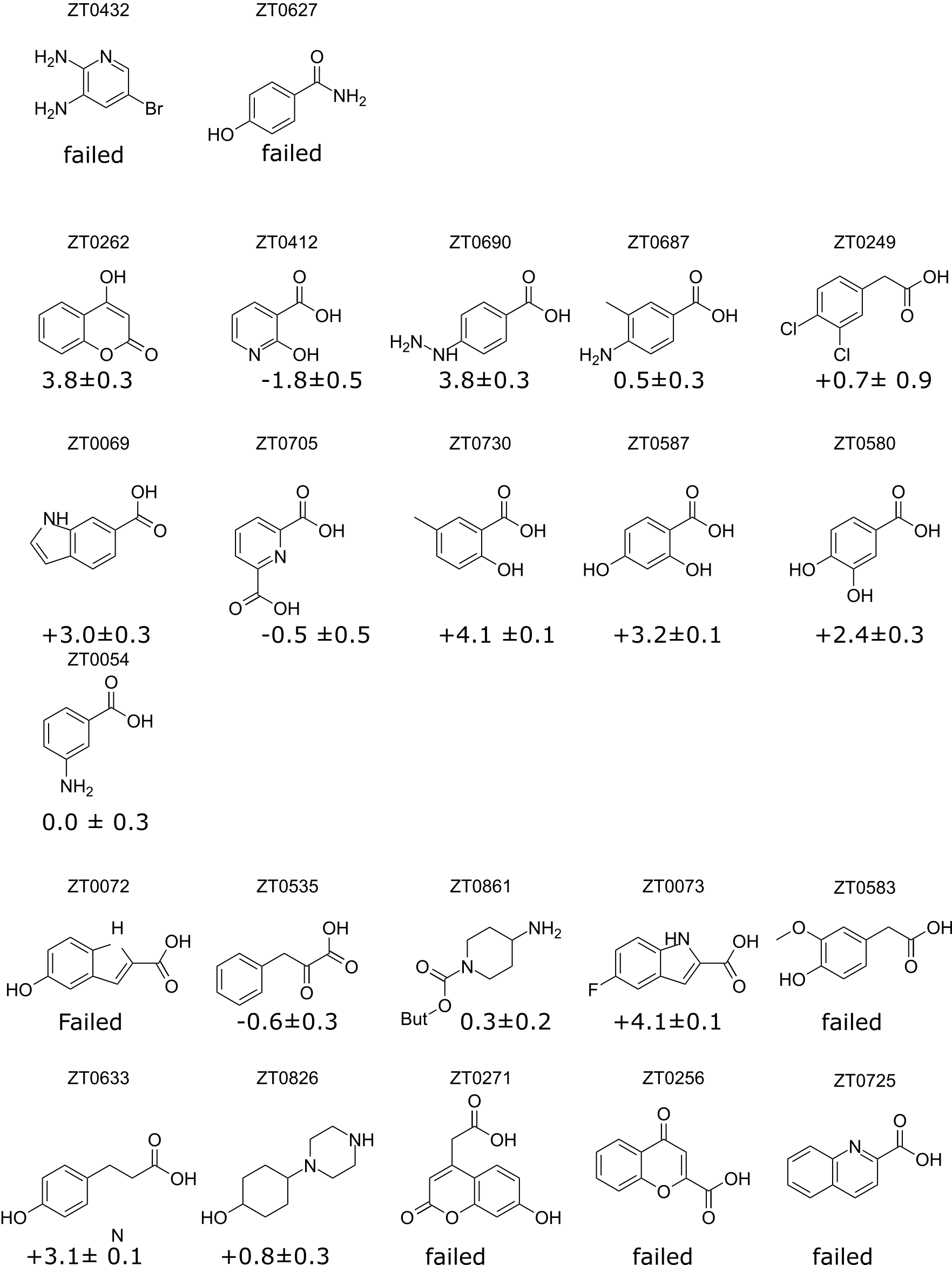
**

**
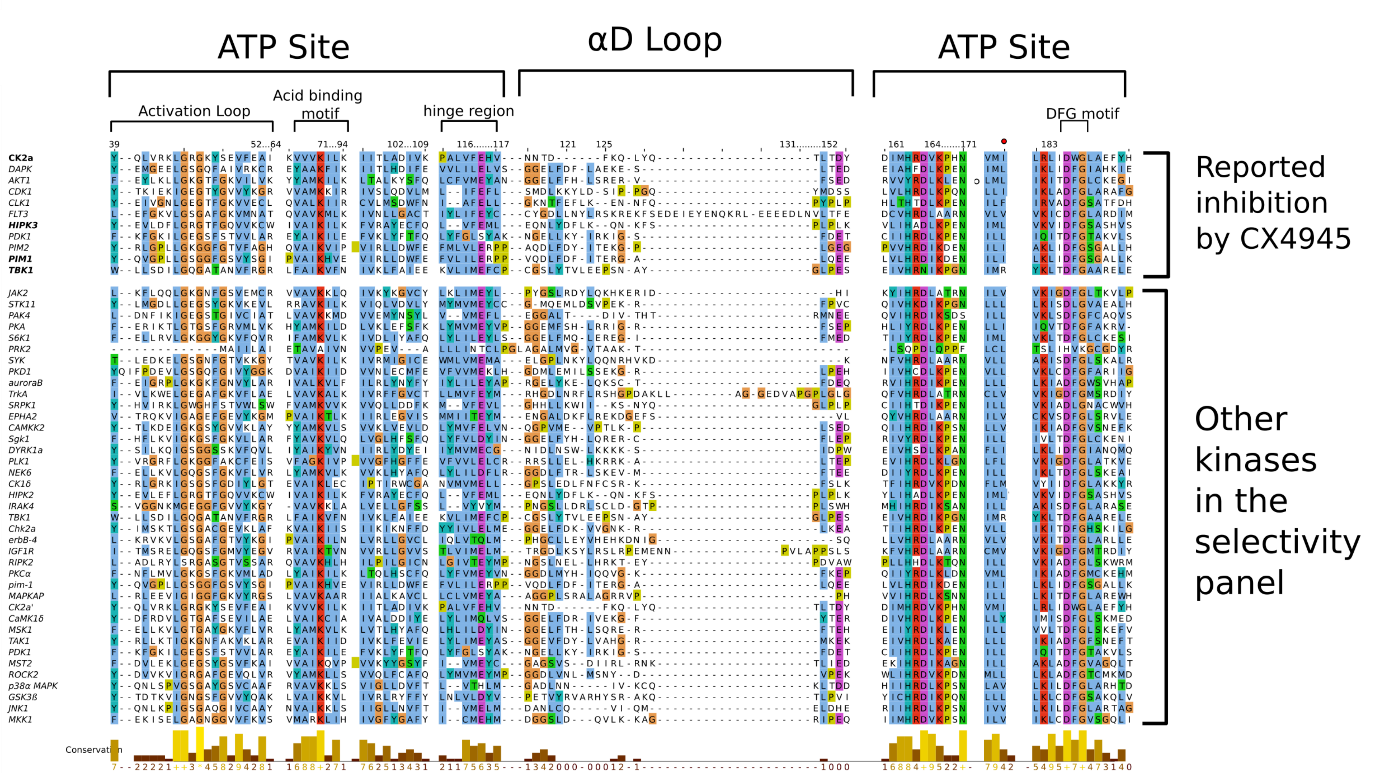
**

Figure SI_1

An alignment of the ATP site and the αD loop of CK2α and a selection of the kinases. The first group of kinases are inhibited by CX4945 with nM potencies. The second set of kinases are the kinases from the selectivity panel used to study CAM4066. These kinases were chosen to give an efficient sampling of the kinome. Alignment is coloured by conservation and the numbering on the top refers to human CK2α.

# **SI_6 Expression and purification of the CK2 holoenzyme**

pGEX-CK2β construct (1-198) obtained from Victor Bolanos-Garcia from Prof. Tom Blundell’s lab was introduced into *Escherichia coli* BL21 (DE3) for protein production. Single colonies of the cells were grown in 6x1 L of 2xTY with 100 μg/mL ampicillin at 37 °C. Isopropyl thio-β-Dgalactopyranoside

(IPTG) was added to a final concentration of 0.4 mM to induce expression when the optical density at 600 nm reached 0.6. The cells were incubated overnight at 25 °C then harvested by centrifugation at 4,000 *g* for 20 minutes. The cell pellets were suspended in 20 mM Tris, 500 mM NaCl, pH 8.5) and lysed using a high pressure homogenizer.

Protease inhibitor cocktail tablets (one tablet per 50 mL extract; Roche Diagnostics) and DNase I

were then added. The crude cell extract was then centrifuged at 10,000 *g* for 45 minutes, the

supernatant was filtered with a 0.22 μm filter. The soluble supernatant was applied on a Glutathione Sepharose column and washed with 5CV of loading buffer (20 mM Tris, 500 mM NaCl, pH 8.5) followed by washing with 10 CV of cleavage buffer (20 mM Tris 500 mM NaCl, 1mM DTT, 1mM EDTA, pH 8.5). 100µL of precission protease was loaded onto the column and incubated for 5 hours at 4°C and eluted in the cleavage buffer. The cleaved protein was further purified on

a Sepharose Q HP anion-exchange column (gradient 0-500mM NaCl) and the main peak fraction from this column was further purified by gel filtration on a Superdex 75 16/60 HiPrep column equilibrated with Tris 20 mM, pH 8.5, 500 mM NaCl. Pure protein was concentrated to 15 mg/mL and flash frozen in liquid nitrogen. The complex was then formed by incubating equal amounts of CK2α (8mg/mL) and CK2β followed by application to a Superdex 200 10/300 column equilibriated with 20mM Tris pH 8.0, 500mM NaCl. The single peak containing the CK2 holoenzyme was concentrated to 1 mg/mL and used in the phosphorylation assay.

# **SI_7 ITC**

All ITC experiments were performed at 25 °C using a MicroCal itc200 instrument (GE Healthcare).

CK2α_WT/CK2 holoenzyme (20 mg/mL, 20mM Tris pH 8.0, 500 mM NaCl) was diluted in Tris buffer (200 mM Tris, 300 mM NaCl, 10% DMSO) and concentrated to 5-10 μM. Ligand (eg CAM4066) in 100x stock solutions was diluted into the buffer ensuring that the DMSO concentrations were carefully matched. In a typical experiment CK2α_WT/CK2 holoenzyme (5 μM) was loaded into the sample cell and 100 μM of the ligand was titrated in nineteen 2 μL injections of 2 s duration at 150 s intervals, with injector speed of 750 rpm. Heats of dilution were determined in identical experiments, but without protein in the cell. The data fitting was performed with a single site binding model using the Origin software package.

# **SI_8 Kinase assays**

The kinase assay was performed using the ADP-Glo™ kinase assay kit (Promega). 50 nM CK2holoenzyme/CK2α_WT was incubated in the kinase reaction buffer (40 mM Tris pH7.5, 200 mM NaCl, 20 mM MgCl2, 25 μM ATP, 50 μM substrate peptide (RRRADDSDDDD, Enzo Life Sciences Inc.), 5% (v/v) DMSO) in the presence of different concentrations of small molecule at 25 °C for 40 min. 5 μL aliquots of the kinase reaction were quenched with 5 μL of ADP-glo™ solution. After another 40 min the kinase detection reagent was added and maintained at 25 °C for 30 minutes. The luminescence was recorded using a PHERAstar FS plate reader (BMG LABTECH) with an integration time of 1 s. Percentage inhibition was calculated relative to a DMSO control and a baseline measurement without ATP. All measurements were performed in triplicate. The IC_50_ curves were fitted using MatLab.

# **SI_9 Crystallography**

|  | |  | |  |  |
| --- | --- | --- | --- | --- | --- |
| PDB code | | 5MMF | | 5MMR | 5MO5 |
| Ligand | | **12** | | **14** | **18** |
| Ligand code in PDB | | **JMB** | | **H83** | **4IH** |
| Construct | | CK2A_FP10 | | CK2A_FP10 | CK2A_FP10 |
| Crystallisation conditions: | | Condition **A** | | Condition **A** | Condition **A** |
| Cryo-cooling conditions | | 50mM **12**, condition A | | 50mM **14**, condition A | 50mM **18**, condition A |
| Soaking conditions | | 50mM **12**, condition A | | 50mM **14**, condition A | 50mM **18**, condition A |
|  | |  | |  |  |
| **Data collection** | |  | |  |  |
| Wavelength (Å) | | 0.96 | | 0.9174 | 0.9763 |
| Resolution range (Å) | | 334.57 - 1.99 (2.00 - 1.99) | | 47.82 - 2.0 (2.10 - 2.0) | 111.50 - 2.04 (2.15 - 2.04) |
| Space group | | C 2 2 21 | | C 2 2 21 | C 2 2 21 |
| Unit cell a, b, c, (Å) α, β, γ (°) | | 64.41 68.63 334.57  90.0 90.0 90.0 | | 64.4 67.7 334.8  90.0 90.0 90.0 | 64.7 68.3 334.5  90.0 90.0 90.0 |
| Total reflections | | 336078 (3372) | | 319453 (44428) | 280162 (40107) |
| Unique reflections | | 51653 (498) | | 50014 (7226) | 47905 (6929) |
| Multiplicity | | 6.5 (6.8) | | 2.0 (2.0) | 5.8 (5.8) |
| Completeness (%) | | 100 (100) | | 0.99 (0.99) | 99.9 (100) |
| Mean I/sigma(I) | | 9.0 (2.2) | | 9.6 (2.8) | 11.7 (2.4) |
| Wilson B-factor | | 32.37 | | 26.56 | 37.35 |
| R-merge | | 0.144 (1.316) | | 0.149 (0.646) | 0.097 (0.792) |
| R-meas | | 0.165 (1.465) | | 0.176 (0.773) | 0.116 (0.959) |
| CC1/2 | | 0.996 (0.794) | | 0.988 (0.869) | 0.999 (0.783) |
|  | |  | |  |  |
| **Refinement** | |  | |  |  |
| Resolution range (High res) (Å) | | 167.29-1.99(2.04-1.99) | | 46.65-2.00(2.05-2.00) | 46.95-2.04(2.09-2.04) |
| Reflections used in refinement | | 51569 (2619) | | 49931 (3640) | 47823 (3268) |
| Reflections used for R-free | | 2619 (170) | | 2553 (188) | 2436 (182) |
| R-work | | 0.2021 (0.2298) | | 0.1958 (0.2122) | 0.1957 (0.2283) |
| R-free | | 0.2185 (0.2519) | | 0.2257 (0.2287) | 0.2127 (0.2421) |
| Number of molecules in the ASU: | | 2 | | 2 | 2 |
| Number of non-hydrogen atoms | | 5832 | | 5915 | 5750 |
| macromolecules | | 5541 | | 5552 | 5547 |
| Ligands | | 56 | | 81 | 46 |
| Protein residues | | 651 | | 651 | 651 |
| RMS (bonds) (Å) | | 0.014 | | 0.014 | 0.015 |
| RMS (angles) (°) | | 1.61 | | 1.61 | 1.68 |
| Ramachandran favored (%) | | 96 | | 97 | 96 |
| Ramachandran allowed (%) | | 3.7 | | 2.4 | 3.5 |
| Ramachandran outliers (%) | | 0 | | 0.15 | 0.15 |
| Clashscore | | 3.63 | | 2.89 | 2.18 |
| Average B-factor | | 50.96 | | 38.15 | 55.48 |
| macromolecules | | 51.22 | | 38.04 | 55.62 |
| ligands | | 50.58 | | 45.58 | 67.93 |
| solvent | | 45.09 | | 38.14 | 46.77 |
|  | |  | |  |  |
| Number of molecules in the ASU: | | 2 | | 2 | 2 |
| Matthews coefficient: | | 2.37 | | 2.34 | 2.37 |
| Solvent content | | 48.24 | | 47.53 | 48.17 |
| Collection date | | 11/10/2014 | | 22/11/2014 | 07/02/2015 |
| Synchotron/X-ray xource | | Diamond Light Source | | Diamond Light Source | Diamond Light Source |
| Beamline | | IO3 | | IO4-1 | IO3 |
| **Crystallisation and cryo conditions** | |  | |  |  |
| A: | | 107mM Mes pH 6.5, 29% glycerol ethoxylate, 1 M ammonium acetate | | | |
| B: | | 112.5mM Mes pH 6.5, 35% glycerol ethoxylate, 180 mM ammonium acetate | | | |
|  | |  | |  |  |
|  | |  | |  |  |
|  | |  | |  |  |
| 5MO6 | | 5MO7 | | 5MO8 | 5MOD |
| 20 | | 16 | | 21 | 3 |
| KXZ | | YRA | | C98 | 86L |
| CK2A_FP10 | | CK2A_FP10 | | CK2A_FP10 | CK2A_FP10 |
| Condition A | | Condition A | | Condition A | Condition A |
| 50mM 20, condition A | | 50mM 16, condition A | | 50mM 21, condition A | 50mM 3, condition A |
| 50mM 20, condition A | | 50mM 20, condition A | | 50mM 20, condition A | 50mM 20, condition A |
|  | |  | |  |  |
|  | |  | |  |  |
| 0.9795 | | 0.9686 | | 0.9763 | 0.9184 |
| 167.08 - 1.825 (1.92 - 1.82) | | 55.98 - 2.16 (2.22 - 2.16) | | 334.92 - 1.82(2.03 - 1.82) | 333.94 - 2.08 (2.40 - 2.08) |
| C 2 2 21 | | C 2 2 21 | | C 2 2 21 | C 2 2 21 |
| 64.5 68.7 334.2  90.0 90.0 90.0 | | 66.35 66.1 335.9  90.0 90.0 90.0 | | 64.6 67.5 334.9  90.0 90.0 90.0 | 64.4 68.7 333.9  90.0 90.0 90.0 |
| 398669 (57402) | | 229830 (15861) | | 439697 (125758) | 293555 (101961) |
| 66530 (9570) | | 40251 (3003) | | 66762 (18802) | 45385 (15673) |
| 6.0 (6.0) | | 5.7 (5.3) | | 6.6 (67) | 6.5 (6.5) |
| 99.8 (100) | | 99.8 (100) | | 100 (100) | 99.9 (100) |
| 10.82 (2.29) | | 9.7 (1.4) | | 16.1 (3.7) | 13.2 (4.0) |
| 29.75 | | 45.63 | | 30.87 | 33.43 |
| 0.06209 (0.2403) | | 0.093 (0.923) | | 0.075 (0.333) | 0.04227 (0.2557) |
| 0.0878 (0.3398) | | 0.101 (1.141) | | 0.089 (0.396) | 0.05977 (0.3616) |
| 0.957 (0.869) | | 0.997 (0.601) | | 0.999 (0.948) | 0.996(0.926) |
|  | |  | |  |  |
|  | |  | |  |  |
| 167.08-1.81(1.86-1.81) | | 55.98-2.15(2.21-2.15) | | 167.46-1.81(1.86-1.81) | 55.66-2.08(2.13-2.08) |
| 66438 (3845) | | 40186 (2583) | | 66669 (4574) | 44991 (2967) |
| 3345 (174) | | 2034 (155) | | 3370 (247) | 2283(150) |
| 0.2211 (0.2924) | | 0.1894 (0.2394) | | 0.2047 (0.2289) | 0.2057 (0.2437) |
| 0.2413 (0.2972) | | 0.2235 (0.2585) | | 0.2190 (0.2394) | 0.2242 (0.2747) |
| 2 | | 2 | | 2 | 2 |
| 5814 | | 5710 | | 5828 | 5790 |
| 5531 | | 5564 | | 5527 | 5562 |
| 43 | | 35 | | 90 | 29 |
| 649 | | 652 | | 649 | 651 |
| 0.014 | | 0.015 | | 0.014 | 0.014 |
| 1.67 | | 1.71 | | 1.59 | 1.61 |
| 96 | | 96 | | 98 | 97 |
| 3.8 | | 4.3 | | 1.8 | 3.1 |
| 0 | | 0 | | 0.31 | 0 |
| 2.82 | | 3.53 | | 2.9 | 1.81 |
| 48.91 | | 64.52 | | 53.61 | 55.02 |
| 49.21 | | 64.64 | | 53.87 | 55.49 |
| 52.95 | | 72.97 | | 59.83 | 52.99 |
| 41.17 | | 55.99 | | 44.06 | 42.3 |
|  | |  | |  |  |
| 2 | | 2 | | 2 | 2 |
| 2.38 | | 2.36 | | 2.34 | 2.37 |
| 48.3 | | 48 | | 47.58 | 48.23 |
| 16/02/2015 | | 12/05/2014 | | 17/09/2015 | 05/04/2014 |
| Diamond Light Source | | Diamond Light Source | | Diamond Light Source | Soleil |
| IO3 | | IO4 | | IO3 | PROXIMA 2 |
|  | |  | |  |  |
|  | |  | |  |  |
|  | |  | |  |  |
|  | |  | |  |  |
|  | |  | |  |  |
|  | |  | |  |  |
| 5MOE | | 5MOH | | 5MOT | 5MOV |
| 6 | | ZT0583 | | ZT0627 | ZT0633 |
| OQC | | YTX | |  |  |
| CK2A_FP10 | | CK2A_KA | | CK2A_KA | CK2A_KA |
| Condition A | | Condition B | | Condition B | Condition B |
| 50mM 6, condition A | | 10 mM ZT0583, condition A | | 10 mM ZT0627, condition A | 10 mM ZT0633, condition A |
| 50mM 20, condition A | | 10 mM ZT0583, condition A | | 10 mM ZT0627, condition A | 10 mM ZT0633, condition A |
|  | |  | |  |  |
|  | |  | |  |  |
| 0.9795 | | 0.96 | | 1.54 | 1.54 |
| 333.47 -1.89 (2.11 - 1.89) | | 59.31 - 1.38 (1.46 - 1.38) | | 35.96 - 2.09 (2.17-2.09) | 35.89 - 2.2 (2.3 - 2.2) |
| C 2 2 21 | | P 1 21 1 | | P 1 21 1 | P 1 21 1 |
| 64.6 68.2 333.5  90.0 90.0 90.0 | | 58.7 45.7 63.8  90.0 111.7 90.0 | | 58.0 45.4 63.4  90.0 111.4 90.0 | 58.1 45.4 63.2  90.0 111.7 90.0 |
| 394839 (112476) | | 176603 (10926) | | 18092(2197) | 15457 (1853) |
| 59855 (16761) | | 58088 (5164) | | 18080 (1705) | 15391 (1456) |
| 6.6 (6.7) | | 3.0 (2.1) | | 4.8(2.0) | 8.0 (4.23) |
| 99.9 (100) | | 99.8 (87.5) | | 98.1 (92.0) | 98.0 (94.7) |
| 20.7 (3.7) | | 13.9 (2.4) | | 27.3(5.9) | 10.59 (1.15) |
| 36.2 | | 17.2 | | 21.21 | 27.73 |
| 0.041 (0.460) | | 0.039 (0.405) | | 0.0812(0.2996) | 0.1446(0.7625) |
| 0.049 (0.544) | | 0.054 (0.555) | | 0.0701(0.4199) | 0.0901(1.0162) |
| 0.999 (0.952) | | 0.998 (0.875) | |  |  |
|  | |  | |  |  |
|  | |  | |  |  |
| 166..74-1.89(1.93-1.89) | | 59.31-1.38(1.41-1.38) | | 35.96-2.09(2.22-2.09) | 35.89-2.20(2.35-2.20) |
| 59769 (4067) | | 58072 (1642) | | 18081 (2587) | 15392 (1455) |
| 3021 (195) | | 2915 (79) | | 925 (136) | 768 (131) |
| 0.1968 (0.2271) | | 0.1966 (0.2233) | | 0.1878 (0.2020) | 0.2057 (0.2732) |
| 0.2084 (0.2551) | | 0.2026 (0.2154) | | 0.2269 (0.2454) | 0.2688 (0.3217) |
| 2 | | 1 | | 1 | 1 |
| 6006 | | 2995 | | 2988 | 2908 |
| 5557 | | 2810 | | 2767 | 2767 |
| 233 | | 21 | | 32 | 12 |
| 648 | | 326 | | 328 | 325 |
| 0.038 | | 0.015 | | 0.02 | 0.015 |
| 3.55 | | 1.64 | | 2.03 | 1.74 |
| 97 | | 96 | | 96 | 96 |
| 3.2 | | 3.3 | | 3 | 4 |
| 0 | | 0.3 | | 0.61 | 0 |
| 2.66 | | 2.15 | | 4.18 | 2.92 |
| 55.12 | | 25.22 | | 25.98 | 33.45 |
| 53.93 | | 25.06 | | 25.6 | 33.54 |
| 88.66 | | 23.83 | | 26.39 | 35.66 |
| 49.36 | | 28.22 | | 31.35 | 31.23 |
|  | |  | |  |  |
| 2 | | 1 | | 1 | 1 |
| 2.36 | | 2.04 | | 1.99 | 1.99 |
| 47.93 | | 39.77 | | 38.36 | 38.18 |
| 28/09/2014 | | 11/10/2014 | | 27/10/2014 | 14/08/2014 |
| Diamond Light Source | | Diamond Light Source | | In House | In House |
| IO3 | | IO3 | | n/a | n/a |
|  | |  | |  |  |
|  | |  | |  |  |
|  | |  | |  |  |
|  | |  | |  |  |
|  | |  | |  |  |
|  | |  | |  |  |
| 5MOW | | 5CU0 | | 5CU2 | 5CT0 |
| ZT0432 | | 9 + 15 | | 19 + 9 | 13 |
| BR9 | | 54R +54G | | 551+54G | 54P |
| CK2A_FP10 | | CK2A_FP10 | | CK2A_FP10 | CK2A_FP10 |
| Condition A | | Condition A | | Condition A | Condition A |
| 10 mM ZT0432, condition A | | 25 mM 8, 5 mM 5, Condition D | | 25 mM 9, 5 mM 5, Condition D | 25 mM 7, Condition D |
| 10 mM ZT0432, condition A | | 25 mM 8, 5 mM 5, Condition D | | 25 mM 9, 5 mM 5, Condition D | 25 mM 7, Condition D |
|  | |  | |  |  |
|  | |  | |  |  |
| 0.92 | | 0.9795 | | 0.9795 | 0.9174 |
| 67.04 - 1.86 (1.96 - 1.86) | | 167.3 - 2.18(2.19-2.1) | | 167.18 - 1.70 (1.71 - 1.70) | 46.19 - 2.01 (2.02 - 2.01) |
| C 2 2 21 | | C 2 2 21 | | C 2 2 21 |  |
| 64.4 68.7 335.2  90.0 90.0 90.0 | | 64.7, 68.3, 334.5  90.0 90.0 90.0 | | 64.8, 68.3, 334.4  90.0 90.0 90.0 | 64.6, 67.4 335.8  90.0 90.0 90.0 |
| 500407 (72587) | | 184707 (1458) | | 473805 (2341) | 316352 (3452) |
| 63101 (9176) | | 36255 (328) | | 73934 (405) | 49184 (508) |
| 7.9 (7.9) | | 5.1 (4.4) | | 6.4 (5.8) | 6.4 (6.8) |
| 99.8 (99.9) | | 92.70 (82.2) | | 90.8 (52.1) | 99.2 (99.8) |
| 16.60 (2.4) | | 7.3 (2.1) | | 15.9 (2.1) | 13.0 (2.1) |
| 31.62 | | 36.76 | | 25.11 | 34.82 |
| 0.061 (0.681) | | 0.142 (0.493) | | 0.069 (0.759) | 0.084 (0.93) |
| 0.069 (0.784) | | 0.157 (0.550) | | 0.076 (0.833) | 0.091 (1.008) |
| 0.999 (0.871) | | 0.990 (0.855) | | 0.998 (0.778) | 0.998 (0.780) |
|  | |  | |  |  |
|  | |  | |  |  |
| 55.87-1.85(1.90-1.85) | | 167.27-2.17 (2.24-2.17) | | 167.18- 1.70 (1.74-1.70) | 46.19-2.00 (2.06-2.00) |
| 63019 (4013) | |  | |  |  |
| 3197 (227) | |  | |  |  |
| 0.1899 (0.2240) | | 0.2044 (0.1992) | | 0.1827 (0.2556) | 0.1998 (0.2229) |
| 0.2037 (0.2341) | | 0.2360 (0.2291) | | 0.2042 (0.2874) | 0.2202 (0.2536) |
| 2 | | 2 | | 2 | 2 |
| 5881 | | 5816 | | 6069 | 5816 |
| 5553 | | 5556 | | 5571 | 5536 |
| 17 | | 70 | | 144 | 46 |
| 652 | | 652 | | 653 | 652 |
| 0.014 | | 0.014 | | 0.014 | 0.014 |
| 1.58 | | 1.76 | | 1.62 | 1.65 |
| 98 | | 96 | | 98 | 97 |
| 1.8 | | 4 | | 2 | 2 |
| 0 | | 0 | | 0 | 0.31 |
| 1.27 | | 2.62 | | 3.78 | 3.45 |
| 48.04 | | 50 | | 39 | 51.7 |
| 48.13 | | 50.2 | | 38.7 | 51.6 |
| 46.8 | | 52.8 | | 42.5 | 78.2 |
| 46.55 | | 44.5 | | 43.1 | 48.8 |
|  | |  | |  |  |
| 2 | | 2 | | 2 | 2 |
| 2.38 | | 2.23 | | 2.23 | 2.21 |
| 48.38 | | 44.89 | | 44.92 | 44.3 |
| 13/07/2014 | | 16/02/15 | | 16/02/15 | 01/02/15 |
| Diamond Light Source | | Diamond Light Source | | Diamond Light Source | Diamond Light Source |
| IO4-1 | | IO2 | | IO2 | IO4-1 |
|  | |  | |  |  |
|  | |  | |  |  |
|  | |  | |  |  |
|  | |  | |  |  |
|  | |  | |  |  |
|  |  | |  |  |  |
| 5CTP | 5CX9 | |  |  |  |
| **15** | **19** | |  |  |  |
| **54R** | **551** | |  |  |  |
| CK2A_FP10 | CK2A_FP10 | |  |  |  |
| Condition **A** | Condition **A** | |  |  |  |
| 25 mM **8**, Condition D | 25 mM **9**, Condition **D** | |  |  |  |
| 25 mM **8**, Condition D | 25 mM **9**, Condition **D** | |  |  |  |
|  |  | |  |  |  |
|  |  | |  |  |  |
| 0.9762 | 0.9174 | |  |  |  |
| 83.47 - 2.03 (2.04 - 2.03) | 46.46 - 1.73 (1.74-73) | |  |  |  |
| C 2 2 21 | C 2 2 21 | |  |  |  |
| 65.1, 68.9, 333.9  90.0 90.0 90.0 | 64.7 68.1, 334.5  90.0 90.0 90.0 | |  |  |  |
| 300842 (2918) | 512458 (5635) | |  |  |  |
| 48911 (469) | 77204 (816) | |  |  |  |
| 6.2 (6.2) | 6.6 (6.9) | |  |  |  |
| 100.0 (98.7) | 99.8 (100.0) | |  |  |  |
| 12.00 (2.3) | 18.8 (2.1) | |  |  |  |
| 37.16 | 25.98 | |  |  |  |
| 0.090 (0.737) | 0.053 (0.926) | |  |  |  |
| 0.099 (0.805) | 0.058 (1.001) | |  |  |  |
| 0.998 (0.744) | 0.999 (0.906) | |  |  |  |
|  |  | |  |  |  |
|  |  | |  |  |  |
| 83.47-2.02 (2.08-2.02) | 46.46-1.73 (1.77-1.73) | |  |  |  |
|  |  | |  |  |  |
|  |  | |  |  |  |
| 0.1848 (0.1997) | 0.1888 (0.2232) | |  |  |  |
| 0.1989 (0.2285) | 0.1982 (0.2363) | |  |  |  |
| 2 | 2 | |  |  |  |
| 5864 | 6096 | |  |  |  |
| 5553 | 5610 | |  |  |  |
| 87 | 152 | |  |  |  |
| 651 | 653 | |  |  |  |
| 0.014 | 0.015 | |  |  |  |
| 1.67 | 1.64 | |  |  |  |
| 98 | 98 | |  |  |  |
| 2 | 2 | |  |  |  |
| 0 | 0 | |  |  |  |
| 2.43 | 3.8 | |  |  |  |
| 56.3 | 44.6 | |  |  |  |
| 56.3 | 44.3 | |  |  |  |
| 70.8 | 54 | |  |  |  |
| 51.3 | 45 | |  |  |  |
|  |  | |  |  |  |
| 2 | 2 | |  |  |  |
| 2.26 | 2.23 | |  |  |  |
| 45.56 | 44.76 | |  |  |  |
| 07/02/15 | 01/02/15 | |  |  |  |
| Diamond Light Source | Diamond Light Source | |  |  |  |
| IO3 | IO4-1 | |  |  |  |
|  |  | |  |  |  |
|  |  | |  |  |  |
|  |  | |  |  |  |
|  |  | |  |  |  |
|  |  | |  |  |  |

1. S. Perez-Rodriguez, M. A. Ortiz, R. Pereira, F. Rodrigues-Barrios, A. R. de Lera and F. J. Piedrafita, Eur. J. Med. Chem., 2009, 44, 2434–2446. [↑](#footnote-ref-1)
2. M. I. Dawson, Z. Xia, G. Liu, J. A. Fontana, L. Farhana, B. B. Patel, S. Arumugarajah, M. Bhuiyan, X.-K. Zhang, Y.-H. Han, W. B. Stallcup, J. Fukushi, T. Mustelin, L. Tautz, Y. Su, D. L. Harris, N. Waleh, P. D. Hobbs, L. Jong, Wan-ru Chao, L. J. Schiff and B. P. Sani, J. Med. Chem., 2007, 50, 2622–2639. [↑](#footnote-ref-2)
3. Synthesis, patent: By Murata, Toshiki et al; From PCT Int. Appl., 2009123194, 08 Oct 2009. Preparation of N-(quinolin-7-yl)benzamide and N-(naphthalen-2-yl)benzamide derivatives as melanin-concentrating hormone receptor antagonists [↑](#footnote-ref-3)
4. Brear, P.; De Fusco, C.; Hadje Georgiou, K.; Francis-Newton, N. J.; Stubbs, C. J.; Sore, H.; Venkitaraman, A.; Abell, C.; Spring, D. R.; Hyvönen, M. *Chem. Sci.* **2016**. [↑](#footnote-ref-4)
